# From plants to nematodes: *Serratia grimesii* BXF1 genome reveals an adaptation to the modulation of multi-species interactions

**DOI:** 10.1099/mgen.0.000178

**Published:** 2018-05-21

**Authors:** Francisco Nascimento, Cláudia Vicente, Peter Cock, Maria Tavares, Márcio Rossi, Koichi Hasegawa, Manuel Mota

**Affiliations:** ^1^​ Nemalab/ICAAM – Instituto de Ciências Agrárias e Ambientais Mediterrânicas, Departamento de Biologia, Universidade de Évora, Núcleo da Mitra, Ap. 94, 7002-554 Évora, Portugal; ^2^​ Information and Computer Sciences, James Hutton Institute, Invergowrie, Dundee DD2 5DA, UK; ^3^​ Department of Environmental Biology, College of Bioscience and Biotechnology, Chubu University, 1200 Matsumoto, Kasugai, Aichi 487-8501, Japan; ^4^​ Departamento de Microbiologia, Laboratório de Bioprocessos, Universidade Federal de Santa Catarina, Florianópolis SC 88040-900, Brazil; ^5^​ Departamento Ciências da Vida, EPCV Universidade Lusófona de Humanidades e Tecnologias, C. Grande 376, Lisboa, 1749-024, Portugal

**Keywords:** *Serratia*, endophytes, nematodes, pine wilt disease, *Bursaphelenchus xylophilus*, plant-growth-promoting bacteria

## Abstract

*Serratia grimesii* BXF1 is a bacterium with the ability to modulate the development of several eukaryotic hosts. Strain BXF1 was isolated from the pinewood nematode, *Bursaphelenchus xylophilus*, the causative agent of pine wilt disease affecting pine forests worldwide. This bacterium potentiates *Bursaphelenchus xylophilus* reproduction, acts as a beneficial pine endophyte, and possesses fungal and bacterial antagonistic activities, further indicating a complex role in a wide range of trophic relationships. In this work, we describe and analyse the genome sequence of strain BXF1, and discuss several important aspects of its ecological role. Genome analysis indicates the presence of several genes related to the observed production of antagonistic traits, plant growth regulation and the modulation of nematode development. Moreover, most of the BXF1 genes are involved in environmental and genetic information processing, which is consistent with its ability to sense and colonize several niches. The results obtained in this study provide the basis to a better understanding of the role and evolution of strain BXF1 as a mediator of interactions between organisms involved in a complex disease system. These results may also bring new insights into general *Serratia* and *Enterobacteriaceae* evolution towards multitrophic interactions.

## Data Summary

The individual Roche Titanium 454 and MiSeq experiment data were deposited in the European Nucleotide Archive (ENA) with the accession numbers ERR2004553 and ERR2004554. The final genome sequence of *Serratia grimesii* BXF1 is available in the ENA under the accession number LT883155.

**Impact Statement**

The pinewood nematode (PWN), *Bursaphelenchus xylophilus*, is the causative agent of pine wilt disease (PWD), which affects pine forests worldwide and leads to huge economic losses. *Serratia* strains were found to be widely associated with the PWN and its insect vector, and can be considered a key component in the PWD complex. *Serratia grimesii* BXF1 was isolated from the PWN, and increased its reproduction. However, strain BXF1 can also act as a plant-growth promoting pine endophyte with fungal and bacterial antagonist activities, further indicating is opportunistic colonization abilities and complex interactions in a wide range of trophic relationships. In this work, we describe and thoroughly analyse the genome sequence of strain BXF1, and discuss several aspects of its ecological roles in soil, in association with plants, nematodes, and, ultimately, the complex PWD system. Additionally, we discuss the evolution and adaptation of *S. grimesii* BXF1 and related strains. Our results provide the basis to better understanding the role of bacteria as mediators of interactions between multiple organisms involved in a complex disease system.

## Introduction

*Serratia* species are ubiquitous to different habitats and show versatile niche occupation abilities. *Serratia* are competent soil and water colonizers, and can also be found associated with a wide range of different hosts, including plants, insects and nematodes, as well as other eukaryotic organisms [1, 2]. Moreover, species and strain-specific characteristics result in different impacts in the *Serratia* hosts.

Despite much knowledge of the ecology and pathogenesis mechanisms of *Serratia marcescens,* the most studied bacterium from the genus *Serratia* [3], not much is understood about the ecology, evolution and general impact of other *Serratia* species. Previous studies demonstrated that, in Portugal, *Serratia* spp. were one of the main bacterial species found associated with the pinewood nematode (PWN), *Bursaphelenchus xylophilus*, which is the causal agent of pine wilt disease (PWD) [4, 5], and, also to its insect-vector, *Monochamus galloprovinciallis* [6]; hence, suggesting a role for *Serratia* species in the PWD complex. In this sense, several reports indicated that some *Serratia* strains (*S. marcescens* and *Serratia liquefaciens*-like) could potentiate nematode infectivity and oxidative stress resistance [7, 8], while others (*S*. *marcescens* and *Serratia plymuthica*-like) were able to kill the PWN [9].

In a recent report, we demonstrated that *Serratia* strain BXF1 (*S. liquefaciens*-like) could promote PWN reproduction when the latter was cultivated in *Botrytis cinerea* [10]. The presence of the chitinase-producing strain BXF1 impacted PWN chitinase-encoding gene expression, suggesting that strain BXF1 facilitates nematode chitin acquisition and consequent nematode feeding. Nevertheless, strain BXF1 was unable to internally colonize the nematode, bind to its cuticle extensively or protect the nematode from xenobiotic stress, all of which have been found to be important processes in symbiotic nematode-bacteria associations [11]; hence, suggesting an opportunistic and transient interaction between BXF1 and the PWN [10]. Interestingly, strain BXF1 promotes pine and tomato plant growth, colonizes internal plant tissues and does not induce PWD symptoms; thus, acting like a plant-growth-promoting endophyte (PGPE). Moreover, strain BXF1 can produce the plant hormone indole-3-acetate (IAA), metabolize benzoate (BA) and phenylacetate (PAA), which are common metabolites found in wilting pine trees [12], and presents increased resistance to several plant defence compounds, such as terpenoids. Its antagonistic activities against fungi and bacteria isolated from wilting maritime pine (*Pinus pinaster*) and other PWN-associated bacteria were also observed [10]. Together with previous observations in studies of *Bursaphelenchus xylophilus*-associated bacteria (reviewed by Nascimento *et al*. [13]), these results indicated that the PWN acquires pine endophytic bacteria inside the pine tree, upon infection and consequent changes in the inner tree environment. Curiously, other eukaryotes, namely corn rootworms, can also acquire *Serratia grimesii* (*S. liquefaciens*-like) strains from plants, which seem to be a reservoir for these strains [14].

The plant microbiome has been shown to play an important role in modulating plant growth and development, as well as stress response and resistance [15]. Pine trees represent an enormous ecological niche for several micro- and macro-organisms, ranging from bacteria to nematodes. Hence, the pine microbiome and its properties may influence the interaction between several organisms, including those able to induce diseases, such as the PWN. Bacteria like strain BXF1, presenting the ability to internally colonize pine trees and possessing versatile colonization abilities, opportunistically bind to the PWN and, consequently, influence some aspects of the interaction between the plant, nematode, fungi and other organisms. Nevertheless, the mechanisms responsible for this bacterial transient opportunistic multi-species colonization and its consequent effects remains elusive.

In this study, we present and comprehensively analyse the genome sequence of strain BXF1. Understanding the genetic mechanisms governing BXF1 functions may bring new insights into the evolution and ecology of *Serratia,* as well as the role of bacterial endophytes in several aspects of complex disease systems involving several eukaryotes.

## Methods

### BXF1 genome sequencing

The BXF1 strain, isolated from a PWN isolate grown in *Botrytis cinerea* plates, was previously characterized [10]. Strain BXF1 genome sequencing was conducted following genomic DNA extraction from an overnight culture using a Qiagen genomic DNA purification kit. The obtained DNA was sequenced using the Roche Titanium 454 system with large-insert 3 kb paired-end libraries (Centre for Genomic Research, University of Liverpool, Liverpool, UK) and the Illumina MiSeq platform (James Hutton Institute, Invergowrie, UK). The Initial assemblies were performed with Roche ‘Newbler’ gsAssembler [16] and mira v4.0.2 [17]. The individual 454 and MiSeq experiment data were deposited in the European Nucleotide Archive (ENA) with the accession numbers ERR2004553 (314 302 reads, totalling 133 Mbp) and ERR2004554 (903 624 paired reads, totalling 231 Mbp), respectively. Ultimately, a final assembly was constructed by combining both 454 and MiSeq data using mira v4.0.2, which resulted in 18 contigs. Guided alignment to published complete genomes, primer walking and PCR reactions were performed to close gaps between the contigs and raise the quality of the genome. However, due to the very repetitive nature of these gap sequences (mostly 16S rRNA) only seven regions could be effectively closed. The final 5 090 820 bp genome sequence of BXF1 is a scaffold of 11 contigs (N50=3 705 947 bp), which in the final assembly were joined using 100 Ns in the repetitive gaps, based on mauve [18] progressive alignments against the complete genome sequence of *Serratia proteamaculans* 568, and as per National Center for Biotechnology Information (NCBI) and ENA submission guidelines. Genome comparisons to other completely sequenced *S. grimesii*-like strains showed the repetitive nature of these regions, further confirming the assembly. The final genome sequence of *S. grimesii* BXF1 is available in the ENA under the accession number LT883155.

### Genome analysis

BXF1 genome annotation was performed using prokka [19]. Functional genome annotation of strain BXF1 was performed using BlastKOALA [20]. Genomic islands (GIs) were predicted in IslandViewer 3 [21]; phage sequences were predicted using phast [22]; effector, secretion systems and secreted protein analysis were performed in EffectiveDB [23]; transcription factor analysis was performed using the P2RP server [24]; CAZymes analysis was executed using default pfam parameters in the BESC data and tools site [25]; protease analysis was performed using the merops peptidase database analysis [26]; antibiotic and secondary metabolite analysis were performed in antiSMASH [27].

All the sequences described in the manuscript and supporting materials were verified individually by blastp searches (default parameters) against the UniProt/SwissProt database (UniProt Consortium, 2017) using Geneious software v.9.1 (http://www.geneious.com [28]).

Comparative genomic analyses were performed using some other *S. grimesii*-like genome sequences (Table 1) available in the NCBI database. The average nucleotide identity (ANI) and average amino acid identity (AAI) between genomes were analysed with online tools available at http://enve-omics.ce.gatech.edu [29], and the two-way analysis scores were presented. Analysis and comparisons of ANI values with other completely sequenced *Serratia* strains were also conducted by using pyani v0.2.3 [30]. Genome circular views and comparisons were performed using brig v0.95 [31].

**Table 1.** Genome sequences from the SLC strains used in this study

Strain	Acession no.	No. of replicons	Genome size (Mbp)	CDS	G+C (mol%)	Isolation	Phenotype	Reference
*S. grimesii* BXF1	GCA_900186025	1	5.08	4696	52.8	*Bursaphelenchus xylophilus*	PGPB, endophyte, nematode-associated	This work
*S. grimesii* NBRC 13537^T^	GCA_001590905.1	1	5.07	4648	52.8	Cheddar cheese	na	NCBI database, unpublished
*S. grimesii* A2	GCA_000734885.1	1	5.13	4354	52.8	Actin buffer solution	na	Mardanova *et al*. [96]
*S. proteamaculans* 568	CP000826.1	2	5.45	4895	55.1	Poplar	PGPB, endophyte	Taghavi *et al*. [97]
*S. liquefaciens* ATCC 27592^T^	CP006252.1	2	5.24	4718	55.4	Milk	na	Nicholson *et al*. [98]

## Results and Discussion

### Strain BXF1 genome main features

The *S. grimesii* BXF1 genome main features are summarized in Table 2.

**Table 2.** *S. grimesii* BXF1 genome main features

**Genome**	**G+C (mol%)**	**CDS**	**tRNA**	**tmRNA**	**GI**	**Phage**	Secreted proteins*									
	52.8	4696	78	1	1	4	73									
**Functional annotation**	**EIF**	**GIF**	**CHM**	**AAM**	**CP**	**MCV**	**EM**	**NM**	**GBM**	**MOA**	**LM**	**XBM**	**MTP**	**BSM**	**OS**	**UNC**
	860	657	328	313	297	194	159	127	86	86	86	60	42	43	34	514
**CAZymes**	**GH**	**GT**	**CBM**	**CE**	**AA**	**PL**										
	378	361	74	60	55	0										
**merops protease**	**A**	**C**	**I**	**M**	**N**	**P**	**S**	**T**	**U**							
	3	28	11	66	2	2	68	6	7							
**Transcription factors**	**TF**	**TCS**	**ODP**													
	406	72	21													
**Secretion systems**	**SecTat**		**I (*prsDEF*)**		**II**		**III**		**IV**		**V**		**VI**			
	+		+		nf		nf		nf		nf		nf			
**antiSMASH**	**NRPS**		**PKS**		**Bacteriocin**		**Siderophore**		**Arylpolyene**		**other**					
	3		1		1		1		1		1					

GI, Genomic island; EIF, environmental information processing; GIF, genetic information processing; CHM, carbohydrate metabolism; AAM, amino acid metabolism; CP, cellular processes; MCV, metabolism of cofactors and vitamins; EM, energy metabolism; NM, nucleotide metabolism; GBM, glycan biosynthesis and metabolism; MOA, metabolism of other amino acids; LM, lipid metabolism; XBM, xenobiotic biodegradation and metabolism; MTP, metabolism of terpenoids and polyketides; BSM, biosynthesis of other secondary metabolites; OS, organismal systems; UNC, unclassified; GH, glycoside hydrolase; GT, glycosyl transferase; CBM, carbohydrate-binding module; CE, carbohydrate esterase; AA, auxiliary activity; PL, polysaccharide lyase; A, aspartic; C, cysteine; I, inhibitor; M, metallo; N, asparagine; P, mixed; S, serine; T, threonine; U, unknown; TF, transcription factor; TCS, two-component system; ODP, other DNA-binding protein; NRPS, non-ribosomal polyketide synthase; PKS, polyketide synthase; nf, not found.

*Predicted in EffectiveDB.

The strain BXF1 genome is constituted by a single circular chromosome with approximately 5.09 Mbp and a mean GC content of 52.8 mol%. A total of 4787 ORFs were predicted, in which 4696 correspond to putative protein coding sequences (CDSs). A total of 78 tRNA, 3 rRNA and 1 tmRNA were detected.

**Fig. 1.**
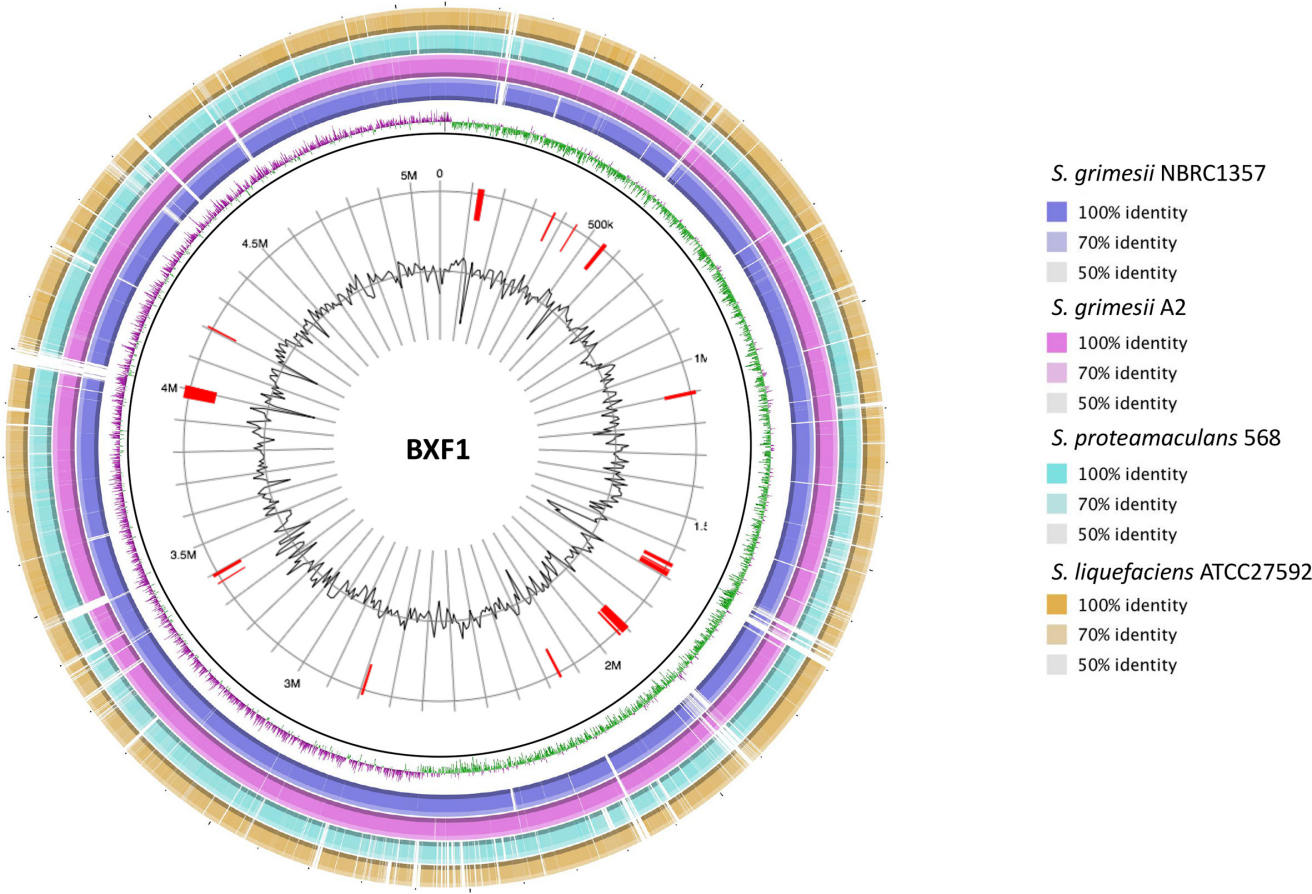
Circular genome representation of *S. grimesii* BXF1 and genome comparisons within the SLC. Predicted GIs in BXF1 genome are represented in red.

BlastKOALA analysis resulted in the functional annotation of 3032 from a total of 4696 CDSs (64.6 %). Environmental (860) and genetic (657) information processing functions were assigned for most of the annotated CDSs, followed by carbohydrate (328), amino acid (313), co-factor and vitamins (194), energy (159), nucleotide (127) and lipid (86) metabolism (Table 2). IslandViewer analysis indicated the presence of several GIs (Table S1, available with the online version of this article) in strain BXF1 genome (Fig. 1). Some of these GIs correspond to phage sequences as found by phast analysis. A total of four phage sequence clusters (three complete and one incomplete phage) were found in BXF1 genome (Table S1).

CAZymes analysis identified 929 proteins (Table S2) belonging to the families of structurally-related catalytic and carbohydrate-binding modules (or functional domains) of enzymes that degrade, modify or create glycosidic bonds (Table 2). A total of 378 proteins were predicted as belonging to the glycoside hydrolase (GH) family, 361 to glycosyl transferases (GTs), 74 to carbohydrate-binding modules (CBMs), 60 to carbohydrate esterases (CEs) and 55 to auxiliary activities (AAs). Proteins belonging to the polysaccharide lyase (PL) family were absent in the BXF1 genome.

Searches in the merops database indicated the presence of 193 proteins (Table S3) with proteolytic or protease inhibition activity in the BXF1 genome (Table 2). The most abundant classes of proteases corresponded to metalloproteases (66, M) and serine proteases (68, S).

Transcription factor analysis indicated the presence of 406 CDSs with this function, 72 CDSs belonged to the two-component systems (TCSs) and 21 were annotated as other DNA-binding proteins (ODPs) (Table 2). The complete elements for *sec* and *tat* secretion systems have been identified. The complete gene set for the protease transport system (*prsDEF*) is also present in BXF1 genome (Table S4). No other elements related to secretion systems were found in BXF1 genome.

### Strain BXF1 phylogeny

Based on its 16S rRNA sequence, strain BXF1 was previously designated as *Serratia quinivorans* [10]. *S. quinivorans,* also known as *S. proteamaculans* subsp. *quinovora,* belongs to the *S. liquefaciens* complex (SLC) [32], a group of bacteria (*S. liquefaciens*, *S. proteamaculans*, *S. quinivorans* and *S. grimesii*) possessing very similar phenotypic and genetic characteristics [33]. Consequently, comparative analysis shows that strain BXF1 genome presents great similarity and synteny with the available genomes of other *Serratia* species belonging to the SLC (Fig. 1), isolated from several different sources (Table 1). In this sense, the strain BXF1 genome presents higher similarity to *S. grimesii* NBRC13537^T^ (ANI 99.01 %, AAI 99.08 %), *S. grimesii* A2 (ANI 99.04 %, AAI 98.94 %), followed by *S. proteamaculans* 568 (ANI 86.97 %, AAI 93.88 %) and *S. liquefaciens* ATCC27592^T^ (ANI 85.53 %, AAI 93.34 %) genomes (Figs S1 and S2). Phenotypic analysis of *S. quinivorans* and *S. grimesii* type strains indicated that only *S. grimesii* can use BA as a sole carbon source [32]. Strain BXF1 uses BA as sole carbon source [10] and possesses the BA degradation genes in its genome (described below). Similarly, BA degradation genes are found in *S. grimesii* NBRC13537 and *S. grimesii* A2, but not in other SLC strains. Interestingly, strain BXF1 and two *S. grimesii* strains do not possess plasmid sequences, while other SLC contain one plasmid. Moreover, the mean genome G+C content is similar between strain BXF1 and *S. grimesii* strains (52.8 mol%) and higher (approx. 55 mol%) in other SLC strains (Table 1). Based on the high genome similarity and phenotypic characterizations, strain BXF1 will be further designated as *S. grimesii* BXF1.

### *S. grimesii* BXF1 genomic traits related to soil and plant colonization

Strain BXF1 can colonize plant internal tissues after soil inoculation. Furthermore, strain BXF1 resisted high concentrations (up to 10 mM) of several metals and aromatic compounds (including xenobiotics like toluene and xylene) commonly found in soils [10]. Multiple genes involved in copper, silver, zinc, cadmium, mercury, cobalt, manganese, nickel, magnesium, molybdate, arsenate, chromate and tellurium transport and tolerance (Table S5) are present in BXF1 genome, which is consistent with its metal resistance/tolerance and its ability to survive in otherwise stressing environments.

Bacteria fiercely compete for iron acquisition in the soil, as it is a very important component for bacterial fitness [34]. Hence, competitive bacteria possess several mechanisms related to iron transport and acquisition, including siderophore production. Strain BXF1 produces siderophores [10]; hence, the presence of aerobactin and enterobactin production operons and related transport and uptake genes, and also several iron transport systems (Table S6). Additionally, a ferrisiderophore reductase gene homologue, which accounts for iron release from siderophores is also present.

Despite the absence of nitrogen fixation genes (*nif*), genes responsible for the assimilatory and dissimilatory nitrate reduction pathways are found in BXF1 genome (Table S7). The *amtB* gene and its regulator which are responsible for ammonia uptake and transport, and other genetic elements for ammonia assimilation are also found. In addition, genes involved in urea degradation through allophanate are found in the BXF1 genome (Table S7).

Strain BXF1 possesses the complete set of genes responsible for the assimilatory sulfate reduction pathway and the sulfate transport system; however, genes necessary for the dissimilatory sulfate reduction pathway are not found. The tetrathionate reduction genes are present in an operon. Strain BXF1 also contains an arylsulfatase and alkyl/arylsulfatase homologues (Table S7), which have been implicated in the degradation of several arylsulfate esters (including xenobiotics like SDS or 4-nitrocathecol) [35].

The phosphate and phosphonate transport systems are found in the genome of BXF1. Furthermore, an operon containing the organophosphonate degradation genes is also present (Table S7). This operon is related to several strains ability to degrade glyphosate [36], a common herbicide found in soils.

Interestingly, the genome of strain BXF1 harbours multiple genes related to aromatic compound degradation (Table S8). The complete BA and catechol degradation pathways are found clustered in a region identified as a GI. The genes responsible for the degradation of protocathecuate, homoprotocatechuate, 4-hydroxybenzoate, PAA and 3- and 4-hydroxyphenylacetate (HPA), which are common key intermediate metabolites in the microbial catabolic pathways of various aromatic compounds, are also present in the genome. The hydroxylated aromatic carboxylic acid exporters genes *aaeAB* may account for this strain ability to deal with stress caused by aromatic compounds.

#### Competition: resistance and antagonistic activities

To compete for ecological niches, bacteria have developed mechanisms to limit the proliferation of competing microbes, such as antibiotic and bacteriocin production [37, 38]. Moreover, some bacteria have developed resistance mechanisms to these compounds. Several antibiotic-resistance genes, antimicrobial peptide (CAMP) resistance genes and multiple multidrug efflux systems can be found in the genome of strain BXF1 (Table S9), which is consistent with its ability to resist several antibiotics [10], and possibly to increase its competitiveness in several environments. Strain BXF1 has been shown to limit the growth of several bacteria and fungi [10]. antiSMASH analysis revealed the presence of several genomic regions encoding secondary metabolites, including a bacteriocin/lantipeptide gene cluster, three non-ribosomal peptide synthase (NRPS) clusters and a type I polyketide synthase (T1PKS) gene (Table S10). Bacteriocins can play a role in mediating the bacterial response to competitors, potentiating its colonization abilities and possibly its ability to sense environmental changes [39]. Interestingly, blast searches indicate that homologues presenting high identity to BXF1 bacteriocin/lantibiotic dehydratase gene are uniquely found in *S. grimesii* strains; thus, indicating that this is a species-specific trait.

One of the NRPS genes identified in BXF1 genome presents high similarity to *S. marcescens* serrawetin W1 production gene, *srwW* [40]. Serrawettins can act like wetting agents on various surfaces; hence, increasing bacterial flagellum dependent and independent movement and swarming motility [41]. Serrawetin (previously described as serratamolide) can also act as an antibiotic [42]. Moreover, the genome of BXF1 also contains the *pswP* gene homologue involved in serrawetin W1 production [43].

Additionally, the genome of BXF1 contains several other genes related to fungal antagonism. These include four chitinase genes, a chitin and *N*-acetylglucosamine-binding protein and a chitobiase (Table S10). Other features that may account for optimal nutrient usage from chitin degradation are present, like chitoporin, the *nagBACD* operon responsible for *N*-acetylglucosamine metabolism, a *N*-acetylglucosamine transmembrane transporter and a chitooligosaccharide deacetylase. Genes encoding pyrrolnitrin and hydrogen cyanide (HCN) production are also present in BXF1 genome. Pyrrolnitrin is a secondary metabolite with known antifungal properties [44], and HCN production is an important trait in the biological control abilities of various bacterial strains [45]. The gene cluster *prnABCD*, encoding pyrrolnitrin, is found in a region classified as a GI. This gene cluster shows high similarity to the *S. plymuthica* (approx. 95–96 %) functional *prnABCD* cluster. Curiously, both pyrrolnitrin and HCN production genes are only found in *S. grimesii* and not in other sequenced SLC bacteria, indicating that in the SLC these traits are specific to *S. grimesii* strains.

#### Root colonization

**Fig. 2.**
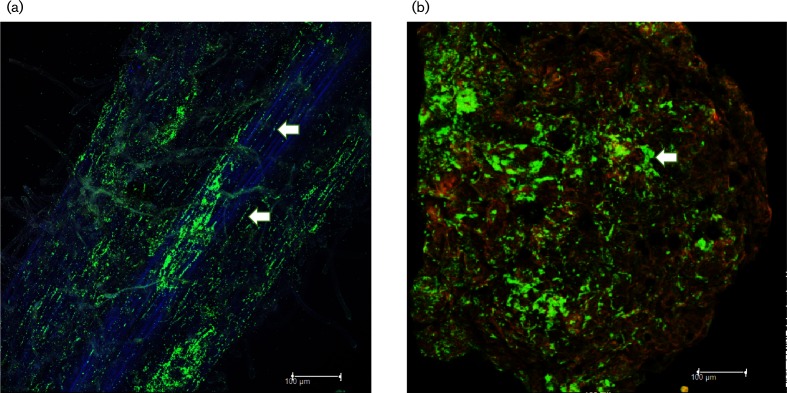
Colonization of common bean roots, 20 days after inoculation by *S. grimesii* BXF1 pn519gfp (a), and common bean root nodules (b). Arrows indicate the presence of bacteria in intracellular spaces and root nodule surface, respectively. Images were acquired by confocal microscopy (Leica Microsystems).

Most bacterial endophytes effectively bind to plant tissues trough the action of adhesins and other elements [46]. Strain BXF1 is motile, and a competent colonizer of the rhizosphere and endosphere [10, 47] (Fig. 2a) and leguminous root nodules (Fig. 2b), which is consistent with the presence of several genes related to motility, chemotaxis and attachment in its genome, including flagella, serrawetin, fimbria, lipopolysaccharide (LPS), exopolysaccharide (EPS), cellulose synthesis and quorum-sensing genes (Table S11). Furthermore, the genome of strain BXF1 also harbours multiple genes responsible for the metabolism of the main root exudates. Root exudates are composed mainly by sugars like glucose, fructose, xylose, arabinose, ribose and maltose, and organic acids such as citrate, malate, fumarate and tartarate, which serve as important carbon sources for rhizospheric and root-associated bacteria [48–50]. Root exudates also contain amino acids, proteins, phenylpropanoids and flavonoids that may modulate plant–microbe interactions [48].

The genome of strain BXF1 harbours multiple genes responsible for the metabolism of the main root exudate sugars (Table S12), such as fructose, xylose, arabinose, ribose and maltose degradation genes. Genes involved in mannose, trehalose, galactose, sucrose, lactose, melibiose, cellobiose, palatinose, maltulose, trehalulose, turanose, leucrose, as well as mannitol, sorbitol, galactitol, maltitol, d-galactosamine and *N*-acetyl-d-galactosamine are also present, further indicating a wide range of sugar utilization by *S. grimesii* BXF1 [10]. Moreover, strain BXF1 contains several genes related to sugar transport systems, which participate in sugar acquisition (Table S13).

Intriguingly, we found homologues of *Sinorhizobium meliloti moc* genes in the BXF1 genome. These are responsible for the transport of rhizopines [51]. The myo-inositol degradation genes, necessary for rhizopine degradation [52], are also present in BXF1 genome (Table S14). Rhizopines are opine-like compounds produced by rhizobial bacteroids in the root nodule. Degradation of rhizopines favours the growth of free-living rhizobia; therefore, increasing their colonization abilities [53].

Strain BXF1 harbours the genetic elements of the TCA and glyoxylate cycle responsible for the degradation/transformation of major organic acids present in root exudates, such as citrate, succinate, malate, fumarate and tartrate. Moreover, the genome also contains the acetate, gluconate, oxalate and formate degradation genes, as well as other genes involved in sugar acid degradation (Table S15). Similarly, several amino acids metabolic pathways (Table S16) and transport genes (Table S17) are also present in the *S. grimesii* BXF1 genome.

Some plant-associated bacteria degrade plant flavonoids, which are known modulators of plant–microbe interactions [54]. These compounds are commonly degraded in a series of dihydroxylation, hydrolysis and oxidation reactions leading to the formation of several compounds, such as protocatechuate, *p*-hydroxybenzoate, *p*-coumarate, PAA and caffeate (B-ring products); and phloroglucinol, phloroglucinol carboxylate, resorcinol and oxaloacetate (A-ring products) [54, 55]. The genome of strain BXF1 contains three quercetin dioxygenase homologues (Table S18), showing high homology to *Escherichia coli* quercetin dioxygenase that converts quercetin (a major flavonoid) into 2-protocatechuoylphloroglucinolcarboxylate [56]. As previously indicated, the genome of BXF1 also contains the protocatechuate, *p*-hydroxybenzoate, PAA and oxaloacetate degradation genes (Tables S8 and S15). In addition, homologues of *nodD* genes, encoding the NodD protein known to bind to flavonoids, are found in BXF1 genome (Table S18).

Major phenylpropanoid degradation pathways [3-phenylpropionate and 3-(3-hydroxyphenyl) propionate] seem to be absent in BXF1 genome. However, a ferulate decarboxylase gene homologue is found (Table S18). Furuya and Kino [57] showed that the HPA monooxygenase enzyme responsible for HPA degradation also demonstrates hydroxylation activity toward tyrosol and various cinnamate derivatives, catalysing the hydroxylation of *p*-coumarate, caffeate, ferulate and coniferaldehyde. Moreover, HPA monooxygenase can also oxidize phenol to catechol, and hydroxylate other phenol derivatives [58]. Hence, the presence of the HPA degradation genes in BXF1 may play a role in the degradation of phenylpropanoids and other phenolic compounds, which are linked to several aspects of plant immunity [59].

A gene encoding a NADPH-dependent curcumin reductase is also found (Table S18). Curcumin reductase is involved in the degradation of the phenolic compound curcumin [60]. Many of these compounds have been shown to possess antimicrobial activity; hence, its degradation by BXF1 may lead to increased bacterial resistance and root colonization abilities.

### Establishment inside the plant and modulation of plant growth

#### Entrance, resistance and maintenance

The passage from the rhizosphere into the endosphere is a process that may naturally occur, where bacteria (opportunistic or true endophytes) can enter to internal plant tissues via root cracks [61]. Strain BXF1 can rapidly colonize internal root tissues (intercellular spaces) upon seed germination and radicle protrusion [10]; however, this colonization is more preeminent in the root differentiation zone, where root crack formation is more common. This is consistent with the absence of major cell-wall-degrading enzymes in the BXF1 genome, as well as typical secretion systems involved in effector delivery into plant cells.

Genes involved in motility, chemotaxis and attachment have been suggested to play an important role in endophytic bacterial colonization [46]. Flagella, fimbria, LPS, EPS, cellulose synthesis and quorum-sensing genes (Table S11) are abundant in BXF1 genome. The genome of BXF1 also contains a gene homologue (SGBXF1_03349) to the *ndvB* gene of *Sinorhizobium meliloti* involved in the production of 1,2-β-glucan. *Sinorhizobium meliloti ndvB* mutants were impaired in nodule invasion and bacteroid development [62]; thus, suggesting a role for this gene in endophytic colonization.

The genome of BXF1 is rich in lytic enzymes (lipases, phospholipases, esterases, proteases, amylases, glucosidases, nucleases) that participate in the modulation of plant cell development and its organization and, consequently, facilitate bacterial entrance, colonization and maintenance. Genes encoding lipolytic enzymes (i.e. lecithinase and other extracellular lipases) (Table S19), proteases (i.e. serralysin and grymelysin) (Table S20), amylases and glucosidases (Table S12) are also found in the genome of BXF1 and are consistent with its degradative abilities [10]. Interestingly, in a search for enzymes with the ability to modulate pine metabolites, we found that the BXF1 *bglC* gene showed homology to *Pinus contorta* coniferin β-glucosidase (35.8 % identity) and to *Arabidopsis thaliana* β-glucosidase 46 (38 % identity) possessing activity against monolignol glucosides like salicin, *p*-coumaryl alcohol glucoside, phenyl-β-d-glucoside, coniferin, syringin and arbutin [63]. Curiously, *S. grimesii* strains were shown to be able to degrade salicin and esculin [32].

#### Resistance against plant defences

Plant defence responses include the production of reactive oxygen species (ROS), reactive nitrogen species, such as nitric oxide (NO), alterations in the plant cell wall and induction of antimicrobial compounds (e.g. secondary metabolites like terpenoids) [64]. In this sense, to colonize internal plant tissues, bacterial endophytes need to be able to cope with these stressful conditions. *S. grimesii* BXF1 genome encodes various enzymes related to ROS detoxification (Table S21), including three superoxide dismutases, two catalases, an alkyl peroxidase, one thyol peroxidase and a hybrid peroxiredoxin. Organic hydroperoxide resistance protein *ohrB* and its regulator *ohrR* are also present. In addition, five glutathione *S*-transferase genes, the glutathione ABC transporter operon, a glutathione peroxidase, glutathione synthetase, glutathione reductase and four glutaredoxin genes are found in BXF1 genome (Table S21). A nitric oxide dioxygenase is also present and may account to the strain ability to deal with nitrosative stress. In addition, antiSMASH analysis revealed the presence of an arylpolyene gene cluster in the genome of strain BXF1 (Table S21). Arylpolyenes may play a role in protecting bacterial cells from exogenous oxidative stress [65].

*S. grimesii* BXF1 grows in the presence of high concentrations of several toxic terpenoids; however, it is unable to use these compounds as sole carbon sources [10]. Not surprisingly, limonene, pinene, geraniol and carvacrol degradation pathways are absent or incomplete in the BXF1 genome. This observation suggests that strain BXF1 must employ different strategies to overcome the toxic effects of terpenoids. This probably occurs trough membrane integrity protection and efficient multidrug efflux systems. For instance, in *Pseudomonas aeruginosa* the *mexAB*–*oprM* efflux system is not only responsible for antibiotic efflux but also for terpenoid efflux [66]. Multidrug efflux systems, including *mexAB–oprM*, are abundant in the BXF1 genome (Table S9).

#### Modulation of plant growth

One of the most studied effects of PGPB relates to their ability to modulate phytohormone levels. In this sense, several studies have pointed to the importance of bacterial production and/or modulation of growth-inducing phytohormones like auxins (commonly IAA) [67] and cytokinins (CKs) [68], as well as in the modulation of phytohormones related to plant defence and stress responses, such as salicylate (SA) [69] and ethylene [70]. Strain BXF1 contains multiple genetic elements involved in phytohormone production, degradation and modulation (Table S22).

Strain BXF1 produces the phytohormone IAA; however, in low levels (approx. 5 µg ml^−1^) [10]. In the strain BXF1 genome an indole-3-pyruvate (IPA) decarboxylase gene is present; hence, suggesting that the demonstrated IAA production in this strain occurs via the IPA pathway. Curiously, a gene showing high similarity to *Pantoea agglomerans* IAA-aspartate hydrolase was also found. The *iaaasp* gene is responsible for the degradation of IAA-aspartate, a common plant IAA conjugate [71]. Moreover, the degradation IAA conjugates modulates free IAA levels necessary to impact plant growth [72]. PAA is also an auxin commonly found in plants [73]. Strain BXF1 possesses the PAA degradation operon.

Genes related to CK production and transformation are abundant in BXF1 genome. The *miaA* gene encoding tRNA isopentenylpyrophosphate transferase is present in BXF1. Großkinsky *et al*. [74] showed that *Pseudomonas fluorescens* G20-18 *miaA* gene is involved in bacterial CK production. Moreover, the authors showed that CK production by *Pseudomonas fluorescens* G20-18 determines biocontrol activity against *Pseudomonas syringae* in *Arabidopsis.* Moreover, the genome of BXF1 also harbours the *miaB* and *miaE* genes that encode enzymes responsible for the production of 2-methylthio-N6-(dimethylallyl)adenosine and 2-methylthio-cis-ribozeatin, respectively. Strain BXF1 also possesses two LOG family proteins, one of them (SGBXF1_02759) presenting high similarity to the LOG of *Corynebacterium glutamicum* (Cg2612) responsible for the production of CK [75]. Additionally, two xanthine dehydrogenase genes showing high similarity to *S. proteamaculans xdhA* and *xdhB* gene, linked to CK biotransformation [76], were also found.

SA plays an important role in plant defence, responses to abiotic stresses and in general plant growth and development [77]. SA can also have important roles in plant–microbe interactions, and bacterial SA production has been previously described in several bacteria [69], including *Serratia* [78]. Moreover, the production of SA has been suggested to play a role in the biocontrol abilities of several bacterial strains [69]. Two isochorismate synthase gene homologues, involved in the convertion of chorismate into isochorismate (the building block for SA synthesis), were found in strain BXF1. An isochorismate-pyruvate lyase gene homologue is also present; hence, suggesting the existence of the complete pathway for SA production in strain BXF1.

Polyamines like putrescine, spermidine and cadaverine have been shown to play a significant role in bacterial plant-growth promotion, and modulation of ethylene biosynthesis *in planta* [79]. The complete pathways to produce putrescine, spermidine and cadaverine were identified in BXF1 genome, as well as the genes responsible for 1,3-diaminopropane synthesis (Table S22). Additionally, two copies of *speG* genes, responsible for spermidine acetylation and protection against polyamine toxicity, and several spermidine export protein genes are also present. Curiously, the polyamine degradation genes found in BXF1 are involved in the formation of the proteinogenic amino acid 4-aminobutyrate (GABA), which plays an important role in plant stress response [80]. While genes responsible for GABA production are found, the GABA permease gene responsible for GABA secretion is absent from the genome of BXF1. However, strain BXF1 possesses the genes involved in GABA degradation through its transformation to succinate, which is consistent to its ability to use GABA as sole carbon source.

*Serratia* spp. are known to produce several volatile organic compounds (VOCs) that have a role in plant–microbe interactions [81]. For instance, *S. proteamaculans* 568 can produce at least 21 VOCs and 16 of these were also found in other *Serratia* strains [82]. Most volatiles are produced as by-products of bacteria metabolism, such as fermentation, sulfur metabolism, amino acid degradation and fatty acid biosynthesis [82, 83], which are abundant pathways in BXF1 genome. Strain BXF1 can ferment several sugars and produces acetoin [10]. For instance, strain BXF1 contains all elements necessary for the mixed acid fermentation pathways (Table S23) that lead to the production of several VOCs, such as ethanol, acetate, lactate and glyoxalate. The genome of BXF1 also harbours the acetolactate synthase (*budA*) and α-acetolactate decarboxylase (*budB*) genes, which are involved in acetoin and 2,3-butanediol production, both VOCs containing plant growth-promoting activities [84]. A gene encoding a pyruvate decarboxylase (*poxB*), which is involved in acetoin production, is also found. Several sulfur modulation pathways are also present in the BXF1 genome (Tables S7 and S23), and may account for its ability to produce sulfur-based volatiles, like dimethylsulfide and H_2_S. In addition, methionine and cysteine metabolism genes are widely present in BXF1 (Table S16) which are linked to the production of sulfur volatiles [82]. The genome of BXF1 harbours several elements that are involved in fatty acid biosynthesis and modification (Table S24), which can play a role in the production of volatile alcohols (e.g. 1-decanol) and ketones (e.g. 2-undecanone), compounds widely produced by *Serratia* species [81]. Furthermore, the genome of strain BXF1 contains several gene homologues to those of the Ehrlich pathway (Table S24), including several aminotransferases and alcohol dehydrogenases, playing a role in the degradation of amino acids and subsequent production of alcohols [85] (e.g. 3-methyl-1-butanol, known to be produced by *Serratia*).

### What features may contribute to an association with the PWN?

**Fig. 3.**
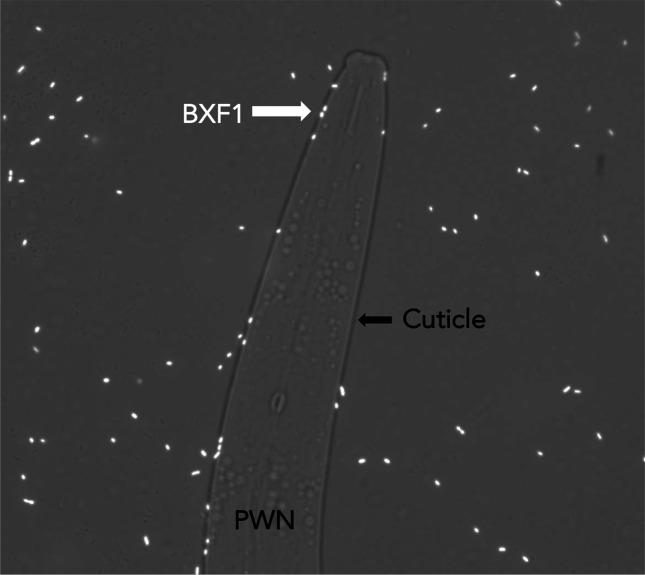
*S. grimesii* BXF1 pn519gfp attachment to the PWN cuticle. Images were acquired by fluorescence microscopy (Leica Microsystems). Magnification=1000x.

Strain BXF1 can bind to the PWN cuticle [10] (Fig. 3). UDP-galactose and UDP-*N*-acetylglucosamine present in nematode surface coat proteins play an important role in mediating the interaction between bacteria and nematode [86]. The genome of strain BXF1 contains several genes related to galactose and *N*-acetylglucosamine degradation, a GlNac-binding protein (SGBXF1_03542) and several *N*-acetylglucosamine and galactose transporters (Table S13). Moreover, the production of LPS, EPS and fimbria (Table S11) (which modulate biofilm production) play a role in strain BXF1 ability to bind to the PWN cuticle. From this ability to bind to the nematode cuticle, it is possible that strain BXF1 gains the advantage of being transported throughout the plant to other nematode feeding sites and other environments. In fact, nematodes may serve as important vectors for bacteria [87].

The PWN feeds on pine cells through the production of a cocktail of several extracellular pectate lyases and cellulases, which lead to plant cell disruption. The PWN genome contains 11 pectate lyase and 11 cellulase genes [88]. Strain BXF1 does not produce any of these enzymes; however, it contains several genes encoding enzymes responsible for the degradation of compounds resulting from plant cell-wall and membrane degradation, such as phospholipids, proteins, cellobiose and glucoronate, as well as cellular contents like DNA, stored sugars and other proteins (Tables S12, S16 S19 and S20). Later in the course of PWD, the host trees become extremely colonized by fungi, which are a major feeding source for the mycophagous PWN [89]. Like the PWN, strain BXF1 presents chitinase activity [10] and possesses several genetic elements responsible for chitin degradation (Table S10).

*S. grimesii* BXF1 has been found to increase PWN reproduction; however, the precise mechanism(s) responsible for this effect is (are) not completely understood. Previous results suggested that strain BXF1 indirectly potentiates nematode feeding by helping the nematode to degrade fungal chitin [10]. However, there is also the possibility that strain BXF1 directly impacts nematode reproduction by the production or degradation of other compounds that regulate nematode development. The PWN suffers several transformations during its life cycle, where hatching and moulting processes occur. In the hatching process, the nematode secretes enzymes (lipases, chitinase, proteases) to digest the egg membranes, which facilitates rupture and consequent nematode escape. After hatching, the nematode grows until becoming limited by the cuticle size. When this occurs, the moulting process initiates, which consists of the synthesis of a new cuticle and the shedding of the older cuticle. The moulting process is assisted by several proteases [90, 91]. Strain BXF1 possesses a wide range of extracellular lytic enzymes, including protease and chitinase [10]. Interestingly, BXF1 lipase 1 (Table S19) shows high homology to *Photorhabdus* lipase, which is induced in the bacterial phase 1 (isolated from infective-stage nematodes), but not in phase 2 (bacterial free-living growth) [92]. The serralysin genes (SGBXF1_00223, SGBXF1_02407) are similar to *S. marcescens* S15 serralysin, which has been implicated in this strain ability to dissolve moths cocoon thus, allowing its better development [93]. Moreover, serralysins degrade gelatin [94], a compound containing collagen (the main component of nematode’s cuticle). Strain BXF1 also encodes grimelysin, an extracellular protease able to degrade filamentous actin [95] and possibly other related compounds that also modulate nematode’s cuticle.

### Did *S. grimesii* BXF1 evolve as a multi-niche colonizer and a multi-interaction mediator?

Overall, genomic data indicates that *S. grimesii* BXF1 evolved as a multi-niche colonizer and a multi-interaction mediator. Its genome is rich in environmental and genetic information processing pathways, clearly indicating an adaptation to several lifestyles and colonization strategies. By being a versatile colonizer, BXF1 can cope with several stresses resulting from this ecological adaptation. Hence, BXF1 contains multiple genes involved in resource acquisition, stress protection and competition, making it a very resilient colonizer and competitor. The carbohydrate, amino acid and lipid metabolism, allied with high chitinolytic, proteolytic and lipolytic activities of *S. grimesii* BXF1, seem to mediate a wide range of interactions with several organisms. One key factor for the neutral or beneficial nature of these interactions may be the absence of typical pathogen secretion systems, which leads to a non-pathogenic phenotype and results in an overall tolerance from its eukaryotic hosts. This is consistent with previous results which indicated that strain BXF1 is unable to kill the PWN, its insect vector, the pine and other plants, even when present in very high concentrations. Contrary to other *Enterobacteriaceae* and *Serratia* strains that contain a wide range of secretion systems, consequently, using ‘brute’ force to colonize its hosts (normally inducing disease), it seems that *S. grimesii* BXF1 evolved as a tolerable bacterium colonizing its hosts in a more ‘friendly’ manner.

Ultimately, the genomic information obtained in this study is essential for the better understanding of the specific contribution of *S. grimesii* BXF1 and related strains in mediating the interactions between multiple organisms involved in a complex disease system; therefore, opening new important research avenues to be explored in the future.

## Data bibliography

1. Nascimento F, Vicente C, Cock P, Tavares M, Rossi M *et al*. Individual Roche Titanium 454 andMiSeq experiment data; European Nucleotide Archive; ERR2004553 and ERR2004554.
2. Nascimento F, Vicente C, Cock P, Tavares M, Rossi M *et al*. Final genome sequence of Serratiagrimesii BXF1; European Nucleotide Archive; LT883155.

## Supplementary Data

Supplementary File 1
