# Supplementary material for: From plants to nematodes: Serratia grimesii BXF1 genome reveals an adaptation to the modulation of multi-species interactions
Source: Microb Genom. 2018 May 21;4(7):e000178. doi: 10.1099/mgen.0.000178 (PMC6113876; doi:10.1099/mgen.0.000178)
Supplement: Supplementary File 1 [file mgen-4-178-s001.pdf]

Supplementary information

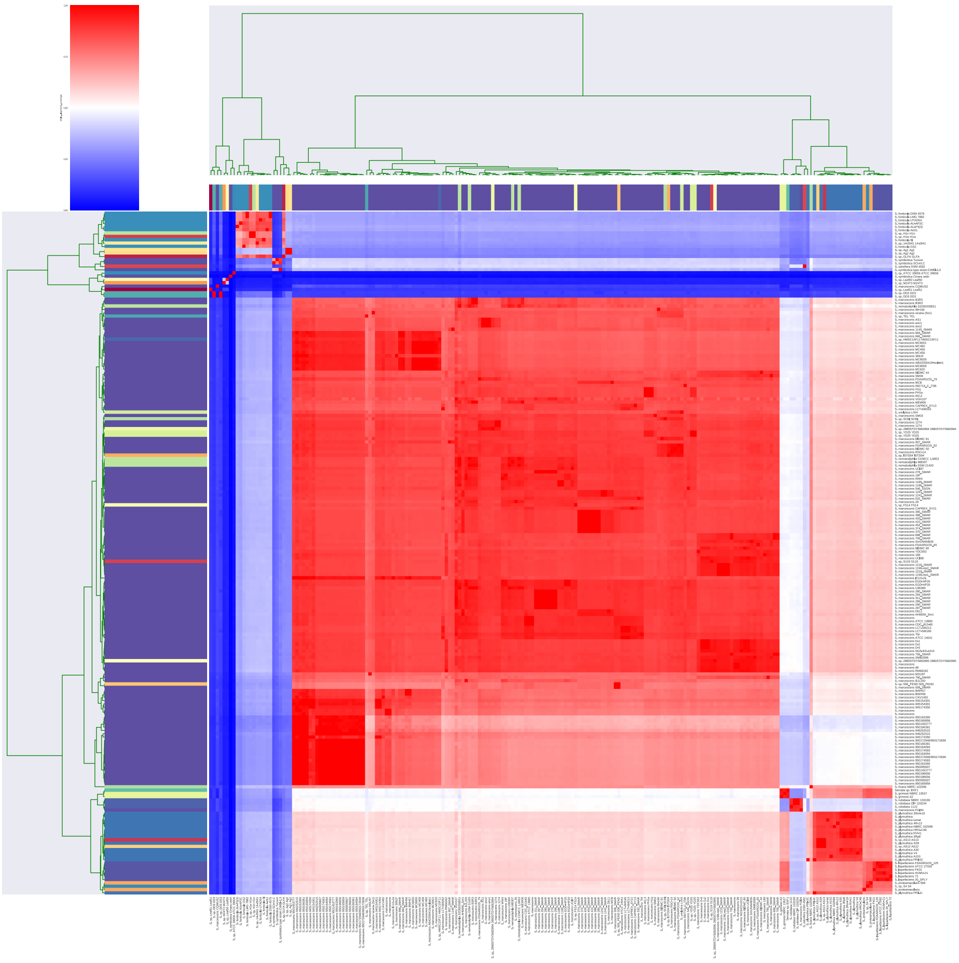

Figure S1- Results obtained from pyani analysis in the comparison of Genome alignment coverage average from over 200 *Serratia* species genomes available in the NCBI database. For purposes of easier identification, strain BXF1 is the only one where the labels list it as *Serratia*, while others are annotated as *S.*

Red colouring indicates a higher alignment coverage and percentage identity between genomes. Blue colouring indicates a lower alignment coverage and percentage identity between genomes.

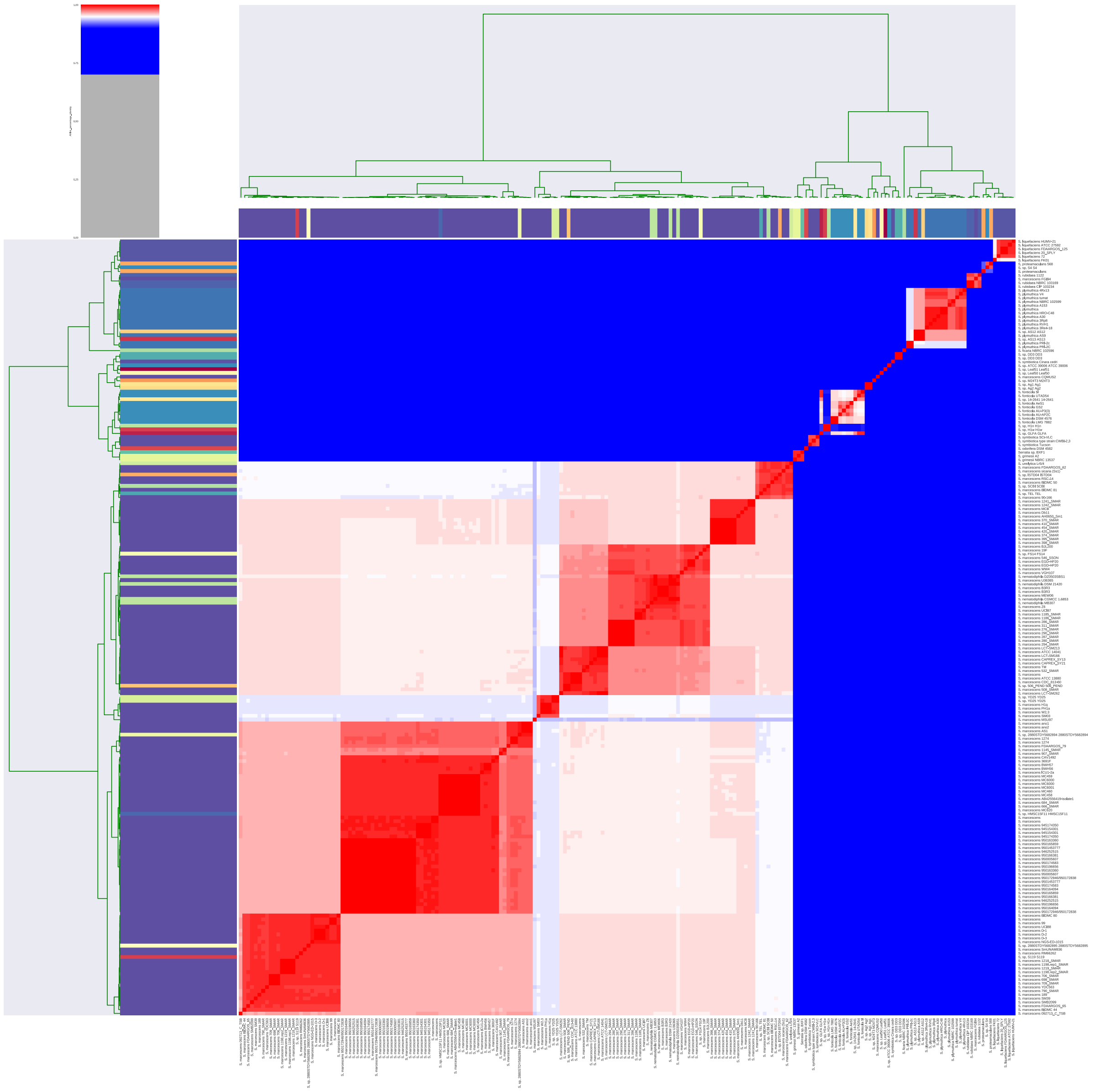

Figure S2- Results obtained from pyani analysis in the comparison of average nucleotide identity (ANI) values from over 200 *Serratia* species genomes available in the NCBI database. For purposes of easier identification, strain BXF1 is the only one where the labels list it as *Serratia*, while others are annotated as *S.*

Red colouring indicates a higher alignment coverage and percentage identity between genomes. Blue colouring indicates a lower alignment coverage and percentage identity between genomes.

Table S1- Genomic islands predicted by Island Viewer and phage sequences predicted by Phast.

Locus	Gene start	Gene end	Strand	Product	External Annotations	
SGBXF1_00106	118994	120193	1	putative transporter YycB	Phage	GI1
SGBXF1_00107	120270	121616	1	hypothetical protein		
SGBXF1_00109	121987	123171	1	Putative prophage CPS-53 integrase		
SGBXF1_00110	123168	123998	1	hypothetical protein		
SGBXF1_00111	124119	124415	1	Prophage CP4-57 regulatory protein (AlpA)		
SGBXF1_00112	124762	124938	1	hypothetical protein		
SGBXF1_00113	124931	125284	1	hypothetical protein		
SGBXF1_00114	125329	125610	1	hypothetical protein		
SGBXF1_00115	125607	125957	1	hypothetical protein		
SGBXF1_00116	125967	128648	1	DNA primase TraC		
SGBXF1_00117	129069	129818	1	hypothetical protein		
SGBXF1_00118	129821	130057	1	DNA-binding transcriptional regulator		
SGBXF1_00119	130359	131639	1	Reverse transcriptase (RNA-dependent DNA polymerase)		
SGBXF1_00120	131630	133651	1	Reverse transcriptase (RNA-dependent DNA polymerase)		
SGBXF1_00121	134184	135638	-1	putative HTH-type transcriptional regulator YdcR		
SGBXF1_00122	135911	136483	1	hypothetical protein		
SGBXF1_00123	136644	136967	1	Inner membrane protein YiaW		
SGBXF1_00124	136973	138109	1	Inner membrane protein YibH		
SGBXF1_00125	138674	139405	1	Transcriptional activator protein EsaR		
SGBXF1_00332	366000	366248	1	hypothetical protein	Pyrrolnitrin	GI2
SGBXF1_00333	366911	368521	1	Flavin-dependent tryptophan halogenase PrnA		
SGBXF1_00334	368521	369603	1	Monodechloroaminopyrrolnitrin synthase PrnB		
SGBXF1_00335	369648	371351	1	Monomeric sarcosine oxidase		
SGBXF1_00336	371373	372467	1	Aminopyrrolnitrin oxygenase PrnD		
SGBXF1_00337	372467	372637	1	FMN reductase (NADH) RutF		
SGBXF1_00338	372634	373878	1	High-affinity Na(+)/H(+) antiporter NhaS3		
SGBXF1_00410	444559	445029	-1	Arginine repressor	-	GI3
SGBXF1_00411	445495	446433	1	Malate dehydrogenase		
SGBXF1_00412	446502	446762	-1	DNA-binding transcriptional regulator Nlp		
SGBXF1_00413	446944	447294	1	Mu DNA-binding domain protein		
SGBXF1_00414	447343	448314	-1	Octaprenyl-diphosphate synthase		
SGBXF1_00415	448584	448895	1	50S ribosomal protein L21		
SGBXF1_00508	547614	548066	-1	DNA polymerase III subunit chi	-	GI4
SGBXF1_00509	548262	549773	-1	Cytosol aminopeptidase		
SGBXF1_00510	550054	551148	1	Lipopolysaccharide export system permease protein LptF		
SGBXF1_00511	551148	552218	1	Lipopolysaccharide export system permease protein LptG		
SGBXF1_00513	552789	554051	1	Prophage CP4-57 integrase		
SGBXF1_00514	554150	556588	1	hypothetical protein		
SGBXF1_00515	557324	557593	1	hypothetical protein		
SGBXF1_00516	557705	558037	-1	hypothetical protein		
SGBXF1_00517	558058	558291	-1	hypothetical protein		
SGBXF1_00518	558767	559672	1	hypothetical protein		
SGBXF1_00519	560008	560643	1	Carbonic anhydrase 1		
SGBXF1_00514	554150	556588	1	hypothetical protein		
SGBXF1_00515	557324	557593	1	hypothetical protein		
SGBXF1_00516	557705	558037	-1	hypothetical protein		
SGBXF1_00517	558058	558291	-1	hypothetical protein		
SGBXF1_00518	558767	559672	1	hypothetical protein		
SGBXF1_01012	1096443	1096757	1	transcriptional regulator BolA	-	GI5
SGBXF1_01013	1097124	1098428	1	Trigger factor		
SGBXF1_01014	1098992	1099615	1	ATP-dependent Clp protease proteolytic subunit precursor		
SGBXF1_01015	1099779	1101050	1	ATP-dependent Clp protease ATP-binding subunit ClpX		
SGBXF1_01016	1101245	1103599	1	Lon protease		
SGBXF1_01017	1103816	1104088	1	DNA-binding protein HU-beta		
SGBXF1_01018	1104271	1106157	1	Peptidyl-prolyl cis-trans isomerase D		
SGBXF1_01019	1106297	1106683	1	ComE operon protein 1		
SGBXF1_01020	1106840	1107256	1	Long-chain acyl-CoA thioesterase FadM		
SGBXF1_01021	1107371	1108069	-1	7-cyano-7-deazaguanine synthase		

SGBXF1_01549	1653087	1654127	1	Polysaccharide biosynthesis/export protein	O-antigen	**GI6**
SGBXF1_01550	1654132	1654566	1	Low molecular weight protein-tyrosine-phosphatase wzb		
SGBXF1_01551	1654579	1656750	1	Tyrosine-protein kinase wzc		
SGBXF1_01552	1656914	1658035	1	Glycosyl transferases group 1		
SGBXF1_01553	1658113	1659531	1	hypothetical protein		
SGBXF1_01554	1659528	1660586	1	D-inositol 3-phosphate glycosyltransferase		
SGBXF1_01555	1660644	1661375	1	hypothetical protein		
SGBXF1_01556	1661952	1663385	1	UDP-glucose:undecaprenyl-phosphate glucose-1-phosphate transferase		
SGBXF1_01557	1663567	1665174	1	hypothetical protein		
SGBXF1_01562	1674316	1675551	1	Putative O-antigen transporter		
SGBXF1_01563	1675551	1676648	1	UDP-galactopyranose mutase		
SGBXF1_01564	1676657	1677640	1	hypothetical protein		
SGBXF1_01565	1677826	1679250	1	Mannose-1-phosphate guanylyltransferase 1		
SGBXF1_01567	1680798	1681811	1	UDP-glucose 4-epimerase		
SGBXF1_01568	1681954	1683018	1	dTDP-glucose 4,6-dehydratase 2		
SGBXF1_01569	1683039	1683908	1	Glucose-1-phosphate thymidylyltransferase 2		
SGBXF1_01570	1683910	1684443	1	dTDP-4-dehydrorhamnose 3,5-epimerase		
SGBXF1_01571	1684443	1685306	1	dTDP-4-dehydrorhamnose reductase		
SGBXF1_01572	1685435	1686298	1	hypothetical protein		
SGBXF1_01573	1686861	1687394	1	dTDP-4-dehydrorhamnose 3,5-epimerase		
SGBXF1_01574	1687502	1688377	1	dTDP-4-dehydrorhamnose reductase		
SGBXF1_01575	1688399	1689187	1	Teichoic acid translocation permease protein TagG		
SGBXF1_01576	1689177	1690568	1	Teichoic acids export ATP-binding protein TagH		
SGBXF1_01577	1690561	1694577	1	?-D-glucose-1-phosphatase		
SGBXF1_01578	1694640	1695566	1	putative glycosyl transferase		
SGBXF1_01579	1695581	1696399	1	N-acetylglucosaminyl-diphospho-decaprenol L-rhamnosyltransferase		
SGBXF1_01580	1696469	1696612	1	hypothetical protein		
SGBXF1_01758	1892516	1893679	-1	Tyrosine recombinase XerD	Phage	**GI7**
SGBXF1_01759	1894061	1894729	-1	hypothetical protein		
SGBXF1_01760	1894726	1894908	-1	hypothetical protein		
SGBXF1_01761	1894924	1895313	-1	hypothetical protein		
SGBXF1_01762	1895772	1896029	-1	hypothetical protein		
SGBXF1_01763	1896088	1896453	-1	hypothetical protein		
SGBXF1_01764	1896450	1896629	-1	hypothetical protein		
SGBXF1_01765	1896632	1897036	-1	hypothetical protein		
SGBXF1_01766	1897036	1897245	-1	hypothetical protein		
SGBXF1_01767	1897242	1897670	-1	HNH endonuclease		
SGBXF1_01768	1897663	1898178	-1	hypothetical protein		
SGBXF1_01769	1898165	1898788	-1	hypothetical protein		
SGBXF1_01770	1898785	1899279	-1	hypothetical protein		
SGBXF1_01760	1894726	1894908	-1	hypothetical protein		
SGBXF1_01761	1894924	1895313	-1	hypothetical protein		
SGBXF1_01762	1895772	1896029	-1	hypothetical protein		
SGBXF1_01763	1896088	1896453	-1	hypothetical protein		
SGBXF1_01764	1896450	1896629	-1	hypothetical protein		
SGBXF1_01765	1896632	1897036	-1	hypothetical protein		
SGBXF1_01766	1897036	1897245	-1	hypothetical protein		
SGBXF1_01767	1897242	1897670	-1	HNH endonuclease		
SGBXF1_01768	1897663	1898178	-1	hypothetical protein		
SGBXF1_01769	1898165	1898788	-1	hypothetical protein		
SGBXF1_01770	1898785	1899279	-1	hypothetical protein		
SGBXF1_01771	1899282	1899425	-1	hypothetical protein		
SGBXF1_01772	1899419	1899637	-1	hypothetical protein		
SGBXF1_01773	1899918	1900016	-1	hypothetical protein		
SGBXF1_01774	1900019	1900195	-1	hypothetical protein		
SGBXF1_01775	1900232	1900570	-1	hypothetical protein		
SGBXF1_01776	1901061	1901204	1	hypothetical protein		
SGBXF1_01777	1901245	1901643	-1	hypothetical protein		
SGBXF1_01778	1901640	1902305	-1	hypothetical protein		
SGBXF1_01779	1902716	1903360	-1	putative HTH-type transcriptional regulator		

SGBXF1_01780	1903452	1903679	1	hypothetical protein		
SGBXF1_01781	1903695	1904021	1	Bacteriophage CII protein		
SGBXF1_01782	1904305	1905069	1	Phage antirepressor protein KilAC domain protein		
SGBXF1_01783	1905072	1905248	1	hypothetical protein		
SGBXF1_01784	1905245	1906267	1	hypothetical protein		
SGBXF1_01785	1906264	1907235	1	hypothetical protein		
SGBXF1_01786	1907586	1907984	1	Phage antitermination protein Q		
SGBXF1_01787	1908238	1908420	1	hypothetical protein		
SGBXF1_01788	1908464	1909096	1	hypothetical protein		
SGBXF1_01789	1909361	1909708	1	hypothetical protein		
SGBXF1_01790	1909979	1910332	1	hypothetical protein		
SGBXF1_01791	1910424	1910660	1	Lysis protein S		
SGBXF1_01792	1910663	1911148	1	Lysozyme RrrD		
SGBXF1_01793	1911145	1911519	1	hypothetical protein		
SGBXF1_01794	1912112	1912366	1	DNA polymerase III subunit theta		
SGBXF1_01795	1912330	1912446	-1	hypothetical protein		
SGBXF1_01796	1912519	1912638	1	hypothetical protein		
SGBXF1_01797	1912719	1913129	1	hypothetical protein		
SGBXF1_01798	1913229	1913906	1	hypothetical protein		
SGBXF1_01799	1913844	1914146	-1	hypothetical protein		
SGBXF1_01800	1914291	1914506	-1	hypothetical protein		
SGBXF1_01801	1914533	1915063	1	Terminase small subunit		
SGBXF1_01802	1915060	1916319	1	Phage terminase large subunit		
SGBXF1_01803	1916375	1916569	1	hypothetical protein		
SGBXF1_01804	1916625	1917953	1	hypothetical protein		
SGBXF1_01805	1917937	1918863	1	Phage Mu protein F like protein		
SGBXF1_01806	1918867	1920132	1	hypothetical protein		
SGBXF1_01816	1925504	1925713	1	hypothetical protein	-	**GI8**
SGBXF1_01817	1925717	1926547	1	hypothetical protein		
SGBXF1_01818	1926695	1926844	1	hypothetical protein		
SGBXF1_01819	1926916	1927821	-1	hypothetical protein		
SGBXF1_01820	1927982	1928266	1	Arc-like DNA binding domain protein		
SGBXF1_01821	1928376	1928657	1	hypothetical protein		
SGBXF1_01822	1928726	1931908	1	hypothetical protein		
SGBXF1_01823	1931911	1932534	1	hypothetical protein		
SGBXF1_02051	2151504	2152403	1	hypothetical protein	-	**GI9**
SGBXF1_02052	2152657	2154159	1	hypothetical protein		
SGBXF1_02053	2154528	2154959	1	hypothetical protein		
SGBXF1_02054	2155014	2155643	1	hypothetical protein		
SGBXF1_02055	2155809	2156933	-1	hypothetical protein		
SGBXF1_02056	2156914	2157159	-1	Excisionase-like protein		
SGBXF1_02057	2157159	2157656	-1	hypothetical protein		
SGBXF1_02058	2158036	2158524	1	hypothetical protein		
SGBXF1_02059	2158970	2159260	-1	hypothetical protein		
SGBXF1_02060	2159543	2159827	1	Acetyltransferase (GNAT) family protein		
SGBXF1_02639	2787422	2788144	1	ChaC-like protein	-	**G10**
SGBXF1_02640	2788248	2788712	1	SnoaL-like polyketide cyclase		
SGBXF1_02641	2788921	2789502	1	Bifunctional transcriptional activator/DNA repair enzyme Ada		
SGBXF1_02642	2789499	2790218	1	hypothetical protein		
SGBXF1_02643	2790221	2790871	1	Alpha-ketoglutarate-dependent dioxygenase AlkB		
SGBXF1_02644	2790947	2791141	1	hypothetical protein		
SGBXF1_02645	2791187	2791837	1	DNA-3-methyladenine glycosylase		
SGBXF1_02646	2791916	2792986	1	Bifunctional transcriptional activator/DNA repair enzyme Ada		
SGBXF1_02647	2793001	2793693	1	hypothetical protein		
SGBXF1_02648	2793710	2794015	1	hypothetical protein		
SGBXF1_02649	2794012	2794773	1	hypothetical protein		
SGBXF1_02650	2794989	2795411	1	LexA repressor		
SGBXF1_03207	3364807	3365022	1	hypothetical protein		
SGBXF1_03208	3365307	3366809	-1	Chaperone protein DnaJ		
SGBXF1_03209	3367295	3367891	-1	hypothetical protein		
SGBXF1_03210	3367977	3368654	1	HTH domain protein		
SGBXF1_03211	3368714	3369160	-1	universal stress protein UspC		
SGBXF1_03229	3389783	3390325	1	hypothetical protein	-	**G11**
SGBXF1_03230	3390383	3391300	-1	HTH-type transcriptional regulator DmlR		
SGBXF1_03231	3391405	3392613	1	4-hydroxybenzoate transporter PcaK		
SGBXF1_03232	3392797	3394392	-1	RNA polymerase sigma factor RpoD		
SGBXF1_03233	3394994	3395920	1	hypothetical protein		
SGBXF1_03234	3396082	3396825	-1	Fatty acyl-CoA reductase		

SGBXF1_03235	3397021	3397482	-1	hypothetical protein		
SGBXF1_03236	3397878	3398270	-1	LexA repressor		
SGBXF1_03237	3398403	3398819	-1	hypothetical protein		
SGBXF1_03238	3398865	3400619	-1	Lipid A export ATP-binding/permease protein MsbA		
SGBXF1_03753	3970473	3970811	1	Outer membrane protein assembly factor BamE precursor	Phage	**G12**
SGBXF1_03754	3970926	3971210	-1	Persistence and stress-resistance antitoxin PasI		
SGBXF1_03755	3971191	3971637	-1	Ribosome association toxin RatA		
SGBXF1_03756	3971799	3972281	1	SsrA-binding protein		
SGBXF1_03758	3972777	3973016	-1	hypothetical protein		
SGBXF1_03759	3973723	3975378	-1	hypothetical protein		
SGBXF1_03760	3975375	3975935	-1	hypothetical protein		
SGBXF1_03761	3975910	3976632	-1	hypothetical protein		
SGBXF1_03762	3976622	3977170	-1	Caudovirales tail fiber assembly protein		
SGBXF1_03763	3977174	3980290	-1	Tail fiber protein		
SGBXF1_03764	3980296	3980901	-1	hypothetical protein		
SGBXF1_03765	3980894	3982078	-1	Baseplate J-like protein		
SGBXF1_03766	3982056	3982403	-1	hypothetical protein		
SGBXF1_03767	3982403	3984934	-1	Phage-related minor tail protein		
SGBXF1_03768	3985122	3985391	-1	hypothetical protein		
SGBXF1_03769	3985539	3985883	-1	hypothetical protein		
SGBXF1_03770	3985883	3986224	-1	hypothetical protein		
SGBXF1_03771	3986211	3986513	-1	Phage holin family 2		
SGBXF1_03772	3986523	3986978	-1	hypothetical protein		
SGBXF1_03773	3986975	3988099	-1	hypothetical protein		
SGBXF1_03774	3988096	3988806	-1	Phage virion morphogenesis family protein		
SGBXF1_03775	3988803	3989306	-1	P2 phage tail completion protein R (GpR)		
SGBXF1_03776	3989303	3989755	-1	Phage head completion protein (GPL)		
SGBXF1_03777	3989855	3990559	-1	Phage small terminase subunit		
SGBXF1_03778	3990566	3991582	-1	Phage major capsid protein, P2 family		
SGBXF1_03779	3991631	3992470	-1	Phage capsid scaffolding protein (GPO) serine peptidase		
SGBXF1_03780	3992780	3994417	1	Terminase-like family protein		
SGBXF1_03781	3994414	3995463	1	Phage portal protein		
SGBXF1_03782	3995514	3995786	1	Ogr/Delta-like zinc finger		
SGBXF1_03783	3995757	3995975	-1	hypothetical protein		
SGBXF1_03784	3996066	3998075	-1	Bacteriophage replication gene A protein (GPA)		
SGBXF1_03785	3998069	3998338	-1	hypothetical protein		
SGBXF1_03786	3998347	3998454	-1	hypothetical protein		
SGBXF1_03787	3998436	3998675	-1	hypothetical protein		
SGBXF1_03788	3998753	3999160	-1	hypothetical protein		
SGBXF1_03789	3999163	3999582	-1	hypothetical protein		
SGBXF1_03790	3999585	3999788	-1	hypothetical protein		
SGBXF1_03791	3999798	4000307	-1	Phage regulatory protein CII (CP76)		
SGBXF1_03792	4000340	4000600	-1	hypothetical protein		
SGBXF1_03793	4000757	4001320	1	Bacteriophage CI repressor helix-turn-helix domain protein		
SGBXF1_03794	4001324	4002391	1	Tyrosine recombinase XerD		
SGBXF1_03795	4002463	4002678	-1	hypothetical protein		
SGBXF1_03796	4002843	4003565	1	Prophage CP4-57 integrase		
SGBXF1_03797	4003935	4006655	1	hypothetical protein		
SGBXF1_03798	4006788	4006994	1	hypothetical protein		
SGBXF1_03799	4007347	4007694	-1	hypothetical protein		
SGBXF1_03800	4007887	4008021	-1	hypothetical protein		
SGBXF1_03801	4008067	4008762	1	hypothetical protein		
SGBXF1_03802	4009221	4009871	1	Metal-binding protein ZinT precursor		
SGBXF1_03792	4000340	4000600	-1	hypothetical protein	-	**GI13**
SGBXF1_03793	4000757	4001320	1	Bacteriophage CI repressor helix-turn-helix domain protein		
SGBXF1_03794	4001324	4002391	1	Tyrosine recombinase XerD		
SGBXF1_03795	4002463	4002678	-1	hypothetical protein		
SGBXF1_03796	4002843	4003565	1	Prophage CP4-57 integrase		
SGBXF1_03797	4003935	4006655	1	hypothetical protein		
SGBXF1_03798	4006788	4006994	1	hypothetical protein		
SGBXF1_03799	4007347	4007694	-1	hypothetical protein		
SGBXF1_03800	4007887	4008021	-1	hypothetical protein		
SGBXF1_03801	4008067	4008762	1	hypothetical protein		

SGBXF1_03802	4009221	4009871	1	Metal-binding protein ZinT precursor		
SGBXF1_03986	4202935	4203747	-1	Histidinol-phosphatase		
SGBXF1_03987	4204062	4204604	-1	NUDIX domain protein		
SGBXF1_03988	4204760	4205983	-1	2'-deamino-2'-hydroxyneamine transaminase		
SGBXF1_03989	4206002	4207042	-1	L-asparagine oxygenase		
SGBXF1_03990	4207749	4208918	1	Multidrug resistance protein MdtL		

Table S2- CAZymes families predicted

Sequence ID	CAZy Families
SGBXF1_04203 Cell division protein FtsP precursor	AA1
SGBXF1_04098 Blue copper oxidase CueO precursor	AA1
SGBXF1_03554 GlcNAc-binding protein A precursor	AA10
SGBXF1_03212 Catalase-peroxidase	AA2
SGBXF1_01943 NADH dehydrogenase	AA3
SGBXF1_04535 Nitrite reductase [NAD(P)H]	AA3
SGBXF1_02157 Gluconate 2-dehydrogenase flavoprotein precursor	AA3
SGBXF1_00215 Anaerobic glycerol-3-phosphate dehydrogenase subunit A	AA3
SGBXF1_02793 D-amino acid dehydrogenase small subunit	AA3
SGBXF1_02368 Fructose dehydrogenase large subunit	AA3
SGBXF1_02080 Gamma-glutamylputrescine oxidoreductase	AA3
SGBXF1_03432 tRNA 5-methylaminomethyl-2-thiouridine biosynthesis bifunctional protein MnmC	AA3
SGBXF1_03592 NADH dehydrogenase-like protein/MT1860	AA3
SGBXF1_01987 L-2-hydroxyglutarate oxidase LhgO	AA3
SGBXF1_04290 Glutamate synthase [NADPH] small chain	AA3
SGBXF1_04107 Dihydrolipoyl dehydrogenase	AA3
SGBXF1_04680 Soluble pyridine nucleotide transhydrogenase	AA3
SGBXF1_04061 Rhodocoxin reductase	AA3
SGBXF1_01737 Fructose dehydrogenase large subunit	AA3
SGBXF1_02474 4-methylaminobutanoate oxidase (formaldehyde-forming)	AA3
SGBXF1_04153 Oxygen-dependent choline dehydrogenase	AA3
SGBXF1_01906 N-methyl-L-tryptophan oxidase	AA3
SGBXF1_01848 Hydrogen cyanide synthase subunit HcnB	AA3
SGBXF1_01482 Oxygen-dependent choline dehydrogenase	AA3
SGBXF1_00504 Alcohol dehydrogenase [acceptor]	AA3
SGBXF1_04578 Aerobic glycerol-3-phosphate dehydrogenase	AA3
SGBXF1_00547 Gamma-glutamylputrescine oxidoreductase	AA3
SGBXF1_04683 Dihydrolipoyl dehydrogenase	AA3
SGBXF1_04644 Glutathione reductase	AA3
SGBXF1_01846 Hydrogen cyanide synthase subunit HcnC precursor	AA3
SGBXF1_01656 Thioredoxin reductase	AA3/CE10
SGBXF1_04258 2,4-dienoyl-CoA reductase [NADPH]	AA3/CE10
SGBXF1_02198 putative FAD-linked oxidoreductase	AA4/AA7
SGBXF1_01719 Outer membrane protein A precursor	AA5
SGBXF1_00611 Peptidoglycan-binding protein ArfA	AA5
SGBXF1_03020 Motility protein B	AA5
SGBXF1_01213 Peptidoglycan-associated lipoprotein precursor	AA5
SGBXF1_00067 putative lipoprotein YiaD precursor	AA5
SGBXF1_03905 Sulfite reductase [NADPH] flavoprotein alpha-component	AA6
SGBXF1_00039 FMN-dependent NADPH-azoreductase	AA6
SGBXF1_03180 Putative NAD(P)H-dependent FMN-containing oxidoreductase YwqN	AA6
SGBXF1_00493 Enamine/imine deaminase	AA6
SGBXF1_00014 Sulfite reductase [NADPH] flavoprotein alpha-component	AA6
SGBXF1_01961 Enamine/imine deaminase	AA6
SGBXF1_03997 Flavodoxin	AA6
SGBXF1_02256 Putative reactive intermediate deaminase TdcF	AA6
SGBXF1_02812 Putative aminoacrylate peracid reductase RutC	AA6
SGBXF1_00484 Enamine/imine deaminase	AA6
SGBXF1_01171 Flavodoxin	AA6
SGBXF1_03107 Putative reactive intermediate deaminase TdcF	AA6
SGBXF1_01704 FMN reductase (NADPH)	AA6
SGBXF1_01103 p-benzoquinone reductase	AA6
SGBXF1_01751 Putative aminoacrylate peracid reductase RutC	AA6
SGBXF1_00742 Sulfite reductase [NADPH] flavoprotein alpha-component	AA6/GH130
SGBXF1_00285 UDP-N-acetylenolpyruvoylglucosamine reductase	AA7
SGBXF1_03048 Catechol 1,2-dioxygenase	CBM12
SGBXF1_02520 Protocatechuate 3,4-dioxygenase alpha chain	CBM12
SGBXF1_02519 Protocatechuate 3,4-dioxygenase beta chain	CBM12
SGBXF1_00631 Chaperone protein DnaK	CBM13
SGBXF1_04357 Rod shape-determining protein MreB	CBM13
SGBXF1_03616 Chaperone protein DnaK	CBM13
SGBXF1_03691 Chaperone protein HscA	CBM13
SGBXF1_01901 Protein YceI	CBM2

SGBXF1_01912 Ribonuclease E	CBM20
SGBXF1_04353 Ribonuclease G	CBM20
SGBXF1_03660 Spermidine N(1)-acetyltransferase	CBM26
SGBXF1_03617 Acetyltransferase (GNAT) family protein	CBM26
SGBXF1_01872 Putative ribosomal N-acetyltransferase YdaF	CBM26
SGBXF1_01495 Putative ribosomal N-acetyltransferase YdaF	CBM26
SGBXF1_02324 hypothetical protein	CBM26
SGBXF1_02783 hypothetical protein	CBM26
SGBXF1_01454 Acetyltransferase (GNAT) family protein	CBM26
SGBXF1_00932 Acetyltransferase (GNAT) family protein	CBM26
SGBXF1_03470 Putative ribosomal N-acetyltransferase YdaF	CBM26
SGBXF1_02844 Putative ribosomal N-acetyltransferase YdaF	CBM26
SGBXF1_04161 Non-hemolytic phospholipase C precursor	CBM32
SGBXF1_01380 Non-hemolytic phospholipase C precursor	CBM32
SGBXF1_00974 Maltodextrin glucosidase	CBM34/GH13
SGBXF1_02944 putative kinase inhibitor	CBM35
SGBXF1_01247 putative kinase inhibitor protein	CBM35
SGBXF1_02134 Tail-specific protease precursor	CBM48
SGBXF1_03976 Curved DNA-binding protein	CBM48
SGBXF1_04582 Glycogen debranching enzyme	CBM48
SGBXF1_00632 Chaperone protein DnaJ	CBM48
SGBXF1_03692 Co-chaperone protein HscB	CBM48
SGBXF1_04237 CYTH domain protein	CBM48
SGBXF1_04583 1,4-alpha-glucan branching enzyme GlgB	CBM48/GH13
SGBXF1_01367 Viral enhancin protein	CBM5
SGBXF1_01620 Putrescine-binding periplasmic protein precursor	CBM50
SGBXF1_04143 2',3'-cyclic-nucleotide 2'-phosphodiesterase/3'-nucleotidase precursor	CBM50
SGBXF1_01417 Lipoprotein E precursor	CBM50
SGBXF1_02561 Fe(3+)-binding periplasmic protein precursor	CBM50
SGBXF1_00532 putative L,D-transpeptidase YbiS precursor	CBM50
SGBXF1_00593 Osmotically-inducible protein Y precursor	CBM50
SGBXF1_04418 Maltose-binding periplasmic protein precursor	CBM50
SGBXF1_02207 putative L,D-transpeptidase YcfS precursor	CBM50
SGBXF1_03145 Phosphoglycerate transport regulatory protein PgtC precursor	CBM50
SGBXF1_01447 DNA protection during starvation protein	CBM50
SGBXF1_00250 sn-glycerol-3-phosphate-binding periplasmic protein UgpB precursor	CBM50
SGBXF1_04635 Trifunctional nucleotide phosphoesterase protein YfkN precursor	CBM50
SGBXF1_01325 molybdate ABC transporter periplasmic molybdate-binding protein	CBM50
SGBXF1_00028 Phosphate-binding protein PstS precursor	CBM50
SGBXF1_03924 N-acetylmuramoyl-L-alanine amidase AmiC precursor	CBM50
SGBXF1_00760 Murein hydrolase activator NlpD precursor	CBM50
SGBXF1_01242 Molybdate-binding periplasmic protein precursor	CBM50
SGBXF1_02661 murein peptide amidase A	CBM50
SGBXF1_02327 fec operon regulator FecR	CBM50
SGBXF1_04491 Bacterioferritin	CBM50
SGBXF1_02896 putative arabinose-binding protein precursor	CBM50
SGBXF1_02821 Murein DD-endopeptidase MepM	CBM50
SGBXF1_01066 Trifunctional nucleotide phosphoesterase protein YfkN precursor	CBM50
SGBXF1_03533 Thiosulfate-binding protein precursor	CBM50
SGBXF1_04713 Sulfate-binding protein precursor	CBM50
SGBXF1_01603 D-alanyl-D-alanine carboxypeptidase DacC precursor	CBM50
SGBXF1_02735 DNA protection during starvation protein	CBM50
SGBXF1_00969 Phosphate-binding protein PstS precursor	CBM50
SGBXF1_01964 Ferritin-1	CBM50
SGBXF1_02599 Putative ABC transporter substrate-binding protein YesO	CBM50
SGBXF1_04728 Murein hydrolase activator EnvC precursor	CBM50
SGBXF1_00395 2',3'-cyclic-nucleotide 2'-phosphodiesterase/3'-nucleotidase precursor	CBM50
SGBXF1_00261 Tyrosine-protein phosphatase precursor	CBM50
SGBXF1_02333 putative ABC transporter-binding protein precursor	CBM50
SGBXF1_02426 sn-glycerol-3-phosphate-binding periplasmic protein UgpB precursor	CBM50
SGBXF1_00370 N-acetylmuramoyl-L-alanine amidase AmiB precursor	CBM50
SGBXF1_00330 Murein hydrolase activator NlpD precursor	CBM50
SGBXF1_00481 hypothetical protein	CBM54
SGBXF1_02511 Transglutaminase-like superfamily protein	CBM54
SGBXF1_04433 Type I phosphodiesterase / nucleotide pyrophosphatase	CBM6
SGBXF1_01126 Rare lipoprotein A precursor	CBM63
SGBXF1_03578 putative hydrolase	CE1

SGBXF1_01033 Metallo-beta-lactamase superfamily protein	CE1
SGBXF1_01693 putative metallo-hydrolase	CE1
SGBXF1_03836 putative quorum-quenching lactonase YtnP	CE1
SGBXF1_04154 Ferri-bacillibactin esterase BesA	CE1
SGBXF1_00042 Metallo-beta-lactamase superfamily protein	CE1
SGBXF1_00833 Hydroxyacylglutathione hydrolase	CE1
SGBXF1_03505 Enterochelin esterase	CE1
SGBXF1_01520 S-formylglutathione hydrolase YeiG	CE1
SGBXF1_01676 Hydroxyacylglutathione hydrolase	CE1
SGBXF1_03953 Metallo-beta-lactamase superfamily protein	CE1
SGBXF1_02568 Carboxylesterase NlhH	CE10
SGBXF1_01212 Protein TolB	CE10
SGBXF1_00843 L-lysine N6-monooxygenase	CE10
SGBXF1_02903 Carboxylesterase NlhH	CE10
SGBXF1_02394 Carboxylesterase NlhH	CE10
SGBXF1_03889 3-hydroxyacyl-[acyl-carrier-protein] dehydratase FabZ	CE11
SGBXF1_00663 RNA polymerase-associated protein RapA	CE11
SGBXF1_00439 ATP-dependent RNA helicase DeaD	CE11
SGBXF1_04765 ATP-dependent DNA helicase RecG	CE11
SGBXF1_01258 UvrABC system protein B	CE11
SGBXF1_01273 ATP-dependent RNA helicase RhlE	CE11
SGBXF1_00700 UDP-3-O-[3-hydroxymyristoyl] N-acetylglucosamine deacetylase	CE11
SGBXF1_02627 ATP-dependent RNA helicase HrpB	CE11
SGBXF1_02853 ATP-dependent RNA helicase DbpA	CE11
SGBXF1_00208 ATP-dependent DNA helicase RecQ	CE11
SGBXF1_04696 Primosomal protein N'	CE11
SGBXF1_00169 ATP-dependent RNA helicase RhlB	CE11
SGBXF1_01716 3-hydroxydecanoyl-[acyl-carrier-protein] dehydratase	CE11
SGBXF1_02405 3-hydroxyacyl-[acyl-carrier-protein] dehydratase FabZ	CE11
SGBXF1_03293 UvrABC system protein B	CE11
SGBXF1_02028 Transcription-repair-coupling factor	CE11
SGBXF1_03744 ATP-dependent RNA helicase SrmB	CE11
SGBXF1_01435 Flavin reductase like domain protein	CE14
SGBXF1_01749 FMN reductase (NADH) RutF	CE14
SGBXF1_00572 4-hydroxyphenylacetate 3-monooxygenase reductase component	CE14
SGBXF1_00903 Flavin reductase like domain protein	CE14
SGBXF1_01372 Acetyl-/propionyl-coenzyme A carboxylase alpha chain	CE4
SGBXF1_02854 Phosphoribosylglycinamide formyltransferase 2	CE4
SGBXF1_01317 D-alanine--D-alanine ligase A	CE4
SGBXF1_00647 Carbamoyl-phosphate synthase large chain	CE4
SGBXF1_00316 Phosphoribosylamine--glycine ligase	CE4
SGBXF1_04093 Poly-beta-1,6-N-acetyl-D-glucosamine N-deacetylase precursor	CE4
SGBXF1_01618 Ribosomal protein S6 modification protein	CE4
SGBXF1_03001 Basal-body rod modification protein FlgD	CE4
SGBXF1_00696 D-alanine--D-alanine ligase	CE4
SGBXF1_00860 Uric acid degradation bifunctional protein	CE4
SGBXF1_02335 Guanine deaminase	CE9
SGBXF1_00492 D-aminoacylase	CE9
SGBXF1_04322 Allantoinase	CE9
SGBXF1_02095 Imidazolonepropionase	CE9
SGBXF1_01910 Dihydroorotase	CE9
SGBXF1_00099 N-isopropylammelide isopropyl amidohydrolase	CE9
SGBXF1_01160 N-acetylglucosamine-6-phosphate deacetylase	CE9
SGBXF1_01035 Adenine deaminase 2	CE9
SGBXF1_04534 Cytosine deaminase	CE9
SGBXF1_01398 N-substituted formamide deformylase precursor	CE9
SGBXF1_00460 Alpha-D-ribose 1-methylphosphonate 5-triphosphate diphosphatase	CE9
SGBXF1_01161 Glucosamine-6-phosphate deaminase	CE9
SGBXF1_02110 Allantoinase	CE9
SGBXF1_01635 3 beta-hydroxysteroid dehydrogenase/Delta 5-->4-isomerase	GH1
SGBXF1_01567 UDP-glucose 4-epimerase	GH1
SGBXF1_01082 UDP-glucose 4-epimerase	GH1
SGBXF1_01568 dTDP-glucose 4,6-dehydratase 2	GH1
SGBXF1_01168 UDP-glucose 4-epimerase	GH1
SGBXF1_01574 dTDP-4-dehydrorhamnose reductase	GH1
SGBXF1_02748 UDP-glucose 4-epimerase	GH1
SGBXF1_02359 Aryl-phospho-beta-D-glucosidase BglC	GH1
SGBXF1_02517 6-phospho-beta-glucosidase BglA	GH1
SGBXF1_00134 3 beta-hydroxysteroid dehydrogenase/Delta 5-->4-isomerase	GH1

SGBXF1_04188 N,N'-diacetylchitobiose-specific phosphotransferase enzyme IIA component	GH1
SGBXF1_00138 6-phospho-beta-glucosidase BglB	GH1
SGBXF1_00771 6-phospho-beta-glucosidase GmuD	GH1
SGBXF1_04187 Aryl-phospho-beta-D-glucosidase BglC	GH1
SGBXF1_00176 dTDP-glucose 4,6-dehydratase 2	GH1
SGBXF1_02396 3 beta-hydroxysteroid dehydrogenase/Delta 5-->4-isomerase	GH1
SGBXF1_01571 dTDP-4-dehydrorhamnose reductase	GH1
SGBXF1_01869 N,N'-diacetylchitobiose-specific phosphotransferase enzyme IIA component	GH1
SGBXF1_02937 GDP-6-deoxy-D-talose 4-dehydrogenase	GH1
SGBXF1_02919 3 beta-hydroxysteroid dehydrogenase/Delta 5-->4-isomerase	GH1
SGBXF1_02308 Mannose-6-phosphate isomerase	GH1
SGBXF1_03476 Glucokinase	GH1
SGBXF1_04530 N,N'-diacetylchitobiose-specific phosphotransferase enzyme IIA component	GH1
SGBXF1_02231 hypothetical protein	GH10
SGBXF1_03172 NAD(P)H azoreductase	GH10
SGBXF1_04119 Protein SprT	GH10/CBM 1
SGBXF1_03920 Membrane-bound lytic murein transglycosylase A precursor	GH102
SGBXF1_01229 Aldose 1-epimerase	GH103
SGBXF1_02802 Membrane-bound lytic murein transglycosylase B precursor	GH103
SGBXF1_02774 Putative glucose-6-phosphate 1-epimerase	GH103
SGBXF1_03463 Membrane-bound lytic murein transglycosylase B precursor	GH103
SGBXF1_02842 Putative oxidoreductase YceM	GH109
SGBXF1_02282 putative oxidoreductase YdgJ	GH109
SGBXF1_02603 Putative oxidoreductase YteT precursor	GH109
SGBXF1_04594 Inositol 2-dehydrogenase/D-chiro-inositol 3-dehydrogenase	GH109
SGBXF1_01397 Glucose--fructose oxidoreductase precursor	GH109
SGBXF1_03415 Thiol:disulfide interchange protein DsbD precursor	GH109
SGBXF1_03220 putative oxidoreductase YdgJ	GH109
SGBXF1_02409 Inositol 2-dehydrogenase	GH109
SGBXF1_00341 Thiol:disulfide interchange protein DsbD precursor	GH109
SGBXF1_02069 Glucose-6-phosphate 3-dehydrogenase	GH109
SGBXF1_04261 1,5-anhydro-D-fructose reductase	GH109
SGBXF1_03928 Protease 3 precursor	GH12
SGBXF1_02598 hypothetical protein	GH123
SGBXF1_03064 hypothetical protein	GH129
SGBXF1_00061 Alpha-amylase precursor	GH13
SGBXF1_02538 Oligo-1,6-glucosidase	GH13
SGBXF1_00991 Riboflavin biosynthesis protein RibD	GH13
SGBXF1_03721 tRNA-specific adenosine deaminase	GH13
SGBXF1_01337 Alpha-amylase precursor	GH13
SGBXF1_03544 transport protein TonB	GH13
SGBXF1_02729 Filamentous hemagglutinin	GH13
SGBXF1_00474 Trehalose-6-phosphate hydrolase	GH13
SGBXF1_01534 Cytidine deaminase	GH13
SGBXF1_03051 Benzoate 1,2-dioxygenase electron transfer component	GH130
SGBXF1_00879 Na(+)-translocating NADH-quinone reductase subunit F	GH130
SGBXF1_04709 Ferredoxin--NADP reductase	GH130
SGBXF1_01640 NADH oxidoreductase hcr	GH130
SGBXF1_00277 NAD(P)H-flavin reductase	GH130
SGBXF1_03094 1,2-phenylacetyl-CoA epoxidase, subunit E	GH130
SGBXF1_03704 Flavohemoprotein	GH130
SGBXF1_01178 KDP operon transcriptional regulatory protein KdpE	GH16
SGBXF1_02620 Transcriptional regulatory protein RstA	GH16
SGBXF1_02040 Transcriptional regulatory protein PhoP	GH16
SGBXF1_00967 Phosphate regulon transcriptional regulatory protein PhoB	GH16
SGBXF1_04010 Swarming motility regulation protein RssB	GH16
SGBXF1_04717 Alkaline phosphatase synthesis transcriptional regulatory protein PhoP	GH16
SGBXF1_03878 Transcriptional activator CadC	GH16
SGBXF1_03405 Transcriptional regulatory protein tctD	GH16
SGBXF1_04089 Transcriptional regulatory protein QseB	GH16
SGBXF1_02938 Heme response regulator HssR	GH16
SGBXF1_01466 DNA-binding transcriptional activator CadC	GH16
SGBXF1_01464 Transcriptional activator protein CzcR	GH16
SGBXF1_03127 DNA-binding transcriptional activator CadC	GH16
SGBXF1_04561 Transcriptional regulatory protein OmpR	GH16
SGBXF1_01474 Transcriptional regulatory protein YycF	GH16
SGBXF1_03526 Transcriptional regulatory protein OmpR	GH16
SGBXF1_03625 Transcriptional regulatory protein BaeR	GH16

SGBXF1_00620 Aerobic respiration control protein ArcA	GH16
SGBXF1_02559 Transcriptional regulatory protein QseB	GH16
SGBXF1_04000 Transcriptional regulatory protein CreB	GH16
SGBXF1_00420 Transcriptional regulatory protein BasR	GH16
SGBXF1_00962 Transcriptional regulatory protein OmpR	GH16
SGBXF1_02400 putative symporter YjmB	GH17
SGBXF1_04682 Hybrid peroxiredoxin hyPrx5	GH18
SGBXF1_01157 Chitinase D precursor	GH18
SGBXF1_00507 Valine--tRNA ligase	GH18
SGBXF1_00637 Isoleucine--tRNA ligase	GH18
SGBXF1_00976 putative peroxiredoxin	GH18
SGBXF1_04072 Thiol-disulfide oxidoreductase YkuV	GH18
SGBXF1_02770 Chitinase A1 precursor	GH18
SGBXF1_03552 Chitinase B precursor	GH18
SGBXF1_01134 Leucine--tRNA ligase	GH18
SGBXF1_03448 Thiol:disulfide interchange protein DsbE	GH18
SGBXF1_01539 Methionine--tRNA ligase	GH18
SGBXF1_02663 Thiol peroxidase	GH18
SGBXF1_03585 Putative peroxiredoxin bcp	GH18
SGBXF1_00148 Chitinase A precursor	GH18
SGBXF1_04307 Inner membrane protein YrbG	GH18
SGBXF1_03550 hypothetical protein	GH19
SGBXF1_03205 Beta-galactosidase	GH2
SGBXF1_01999 Evolved beta-galactosidase subunit alpha	GH2
SGBXF1_02721 Tryptophan synthase alpha chain	GH2
SGBXF1_03268 Endonuclease 4	GH20
SGBXF1_01166 Chitobiase precursor	GH20
SGBXF1_04601 Xylose isomerase-like TIM barrel	GH20
SGBXF1_02570 Inosose dehydratase	GH20
SGBXF1_04592 Inosose dehydratase	GH20
SGBXF1_04593 Xylose isomerase-like TIM barrel	GH20
SGBXF1_02410 Putative hydroxypyruvate isomerase YgbM	GH20
SGBXF1_01456 Putative hydroxypyruvate isomerase YgbM	GH20
SGBXF1_04038 L-ribulose-5-phosphate 3-epimerase UlaE	GH20
SGBXF1_03648 Xylose isomerase-like TIM barrel	GH20
SGBXF1_03219 Xylose isomerase-like TIM barrel	GH20
SGBXF1_02068 Inosose dehydratase	GH20
SGBXF1_00479 Beta-hexosaminidase	GH20
SGBXF1_01936 Cystine-binding periplasmic protein precursor	GH23
SGBXF1_04191 Endo-type membrane-bound lytic murein transglycosylase A precursor	GH23
SGBXF1_01141 Glutamate/aspartate periplasmic-binding protein precursor	GH23
SGBXF1_01318 Lysine-arginine-ornithine-binding periplasmic protein precursor	GH23
SGBXF1_00033 ABC transporter glutamine-binding protein GlnH precursor	GH23
SGBXF1_02967 Cystine-binding periplasmic protein precursor	GH23
SGBXF1_02155 Endo-type membrane-bound lytic murein transglycosylase A precursor	GH23
SGBXF1_04383 Membrane-bound lytic murein transglycosylase F precursor	GH23
SGBXF1_03720 Membrane-bound lytic murein transglycosylase F precursor	GH23
SGBXF1_02418 Lysine-arginine-ornithine-binding periplasmic protein precursor	GH23
SGBXF1_03391 Lysine-arginine-ornithine-binding periplasmic protein precursor	GH23
SGBXF1_03767 Phage-related minor tail protein	GH23
SGBXF1_01802 Phage terminase large subunit	GH23
SGBXF1_02433 Cystine-binding periplasmic protein precursor	GH23
SGBXF1_01703 Putative aliphatic sulfonates-binding protein precursor	GH23
SGBXF1_01629 Putative ABC transporter arginine-binding protein 2 precursor	GH23
SGBXF1_00832 Membrane-bound lytic murein transglycosylase D precursor	GH23
SGBXF1_00614 Soluble lytic murein transglycosylase precursor	GH23
SGBXF1_03244 Lysine-arginine-ornithine-binding periplasmic protein precursor	GH23
SGBXF1_01626 ABC transporter arginine-binding protein 1 precursor	GH23
SGBXF1_04394 Cyclohexadienyl dehydratase precursor	GH23
SGBXF1_01444 Glutamine-binding periplasmic protein precursor	GH23
SGBXF1_00854 Cystine-binding periplasmic protein precursor	GH23
SGBXF1_04138 Membrane-bound lytic murein transglycosylase C precursor	GH23
SGBXF1_00753 Glutamine-binding periplasmic protein precursor	GH23
SGBXF1_01224 Autoinducer 2 sensor kinase/phosphatase LuxQ	GH23/GT2
SGBXF1_01792 Lysozyme RrrD	GH24
SGBXF1_03316 Lysozyme RrrD	GH24
SGBXF1_01692 Peptidase M15	GH24
SGBXF1_04197 Lysozyme RrrD	GH24
SGBXF1_04704 Glycerol uptake facilitator protein	GH27

SGBXF1_00936 Aquaporin Z	GH27
SGBXF1_02342 Beta-lactamase	GH3
SGBXF1_01321 Periplasmic beta-glucosidase precursor	GH3
SGBXF1_03730 Holo-[acyl-carrier-protein] synthase	GH3
SGBXF1_02302 Beta-lactamase	GH3
SGBXF1_01941 Beta-hexosaminidase	GH3
SGBXF1_00573 D-alanyl-D-alanine-carboxypeptidase/endopeptidase AmpH precursor	GH3
SGBXF1_00945 Shikimate kinase 2	GH3
SGBXF1_02381 D-alanyl-D-alanine dipeptidase	GH3
SGBXF1_04545 Shikimate kinase 1	GH3
SGBXF1_02306 Esterase EstB	GH3
SGBXF1_03195 4'-phosphopantetheinyl transferase Npt	GH3
SGBXF1_02399 Thermostable beta-glucosidase B	GH3
SGBXF1_02093 D-aminopeptidase	GH3
SGBXF1_03340 Tyrosine-specific transport protein	GH31
SGBXF1_04253 Tyrosine-specific transport protein	GH31
SGBXF1_03277 Tryptophan-specific transport protein	GH31
SGBXF1_03666 Alpha-xylosidase	GH31
SGBXF1_01069 TraB family protein	GH31
SGBXF1_01871 Lichenan permease IIC component	GH32
SGBXF1_02002 Pseudouridine kinase	GH32
SGBXF1_03725 PTS system EIIBC component	GH32
SGBXF1_01928 PTS system glucose-specific EIICB component	GH32
SGBXF1_02330 putative sugar kinase YdjH	GH32
SGBXF1_00475 PTS system trehalose-specific EIIBC component	GH32
SGBXF1_04175 Sucrose-6-phosphate hydrolase	GH32
SGBXF1_01063 Inosine-guanosine kinase	GH32
SGBXF1_04193 pfkB family carbohydrate kinase	GH32
SGBXF1_02245 Pyridoxamine kinase	GH32
SGBXF1_01162 PTS system glucose-specific EIICBA component	GH32
SGBXF1_00004 Ribokinase	GH32
SGBXF1_00770 N,N'-diacetylchitobiose permease IIC component	GH32
SGBXF1_04172 2-dehydro-3-deoxygluconokinase	GH32
SGBXF1_00137 PTS system beta-glucoside-specific EIIBCA component	GH32
SGBXF1_04596 5-dehydro-2-deoxygluconokinase	GH32
SGBXF1_00064 2-dehydro-3-deoxygluconokinase	GH32
SGBXF1_00961 putative sugar kinase YdjH	GH32
SGBXF1_00075 PTS system mannitol-specific EIICBA component	GH32
SGBXF1_03270 Tagatose-6-phosphate kinase	GH32
SGBXF1_02358 PTS system beta-glucoside-specific EIIBCA component	GH32
SGBXF1_04189 Lichenan permease IIC component	GH32
SGBXF1_02299 PTS system maltose- and glucose-specific EIICB component	GH32
SGBXF1_04174 Negative regulator of SacY activity	GH32
SGBXF1_02100 Sucrose-6-phosphate hydrolase	GH32
SGBXF1_04661 2-dehydro-3-deoxygluconokinase	GH32
SGBXF1_04235 Bifunctional protein HldE	GH32/GT9
SGBXF1_01843 4-hydroxy-tetrahydrodipicolinate synthase	GH33
SGBXF1_02247 4-hydroxy-tetrahydrodipicolinate synthase	GH33
SGBXF1_03583 4-hydroxy-tetrahydrodipicolinate synthase	GH33
SGBXF1_03279 Mannonate dehydratase	GH35
SGBXF1_00531 Right origin-binding protein	GH39
SGBXF1_02566 HTH-type transcriptional activator Btr	GH39
SGBXF1_02412 Regulatory protein PchR	GH39
SGBXF1_01844 HTH-type transcriptional regulator ChbR	GH39
SGBXF1_03150 HTH-type transcriptional regulator CdhR	GH39
SGBXF1_01948 Right origin-binding protein	GH39
SGBXF1_02975 Urease operon transcriptional activator	GH39
SGBXF1_04332 HTH-type transcriptional repressor of iron proteins A	GH39
SGBXF1_03402 HTH-type transcriptional repressor of iron proteins A	GH39
SGBXF1_02281 Arabinose operon regulatory protein	GH39
SGBXF1_02271 Right origin-binding protein	GH39
SGBXF1_02398 HTH-type transcriptional activator Btr	GH39
SGBXF1_02625 HTH-type transcriptional repressor of iron proteins A	GH39
SGBXF1_03615 DNA-3-methyladenine glycosylase 2	GH39
SGBXF1_00618 Right origin-binding protein	GH39
SGBXF1_02372 Transposon Tn10 TetD protein	GH39
SGBXF1_02882 Regulatory protein SoxS	GH39
SGBXF1_00909 Transcriptional activator FeaR	GH39
SGBXF1_03818 HTH-type transcriptional activator RhaR	GH39

SGBXF1_01890 HTH-type transcriptional regulator GadX	GH39
SGBXF1_00570 HTH-type transcriptional activator Btr	GH39
SGBXF1_02261 Transcriptional activator FeaR	GH39
SGBXF1_00946 HTH-type transcriptional repressor of iron proteins A	GH39
SGBXF1_04184 HTH-type transcriptional activator RhaS	GH39
SGBXF1_01334 HTH-type transcriptional activator RhaR	GH39
SGBXF1_01491 Right origin-binding protein	GH39
SGBXF1_03981 HTH-type transcriptional activator RhaS	GH39
SGBXF1_00772 HTH-type transcriptional regulator ChbR	GH39
SGBXF1_03133 Bifunctional transcriptional activator/DNA repair enzyme AdaA	GH39
SGBXF1_02646 Bifunctional transcriptional activator/DNA repair enzyme Ada	GH39
SGBXF1_00529 HTH-type transcriptional repressor of iron proteins A	GH39
SGBXF1_01298 HTH-type transcriptional regulator CdhR	GH39
SGBXF1_04117 Right origin-binding protein	GH39
SGBXF1_02540 Alpha-glucosidase	GH4
SGBXF1_03132 Alpha-galactosidase	GH4
SGBXF1_01870 putative 6-phospho-beta-glucosidase	GH4
SGBXF1_04531 putative 6-phospho-beta-glucosidase	GH4
SGBXF1_04774 Glutamine synthetase	GH5
SGBXF1_00526 Gamma-glutamylputrescine synthetase PuuA	GH5
SGBXF1_03255 Gamma-glutamylputrescine synthetase PuuA	GH5
SGBXF1_02084 Gamma-glutamylputrescine synthetase PuuA	GH5
SGBXF1_02027 Diguanylate cyclase DosC	GH53
SGBXF1_02411 D-galactose-binding periplasmic protein precursor	GH53
SGBXF1_03946 HTH-type transcriptional regulator GalR	GH53
SGBXF1_00129 HTH-type transcriptional regulator DegA	GH53
SGBXF1_00477 HTH-type transcriptional regulator TreR	GH53
SGBXF1_02901 putative HTH-type transcriptional repressor ExuR	GH53
SGBXF1_04697 HTH-type transcriptional repressor CytR	GH53
SGBXF1_01528 HTH-type transcriptional regulator GalS	GH53
SGBXF1_02067 Catabolite control protein A	GH53
SGBXF1_04588 HTH-type transcriptional regulator GntR	GH53
SGBXF1_03668 Ribose operon repressor	GH53
SGBXF1_00003 Ribose operon repressor	GH53
SGBXF1_01538 Periplasmic binding proteins and sugar binding domain of LacI family protein	GH53
SGBXF1_03948 HTH-type transcriptional regulator AscG	GH53
SGBXF1_01529 D-galactose-binding periplasmic protein precursor	GH53
SGBXF1_01986 D-ribose-binding periplasmic protein precursor	GH53
SGBXF1_04358 RNase E specificity factor CsrD	GH53
SGBXF1_04229 Maltose regulon regulatory protein MalI	GH53
SGBXF1_01104 putative diguanylate cyclase YdaM	GH53
SGBXF1_04031 hypothetical protein	GH53
SGBXF1_00582 putative diguanylate cyclase YcdT	GH53
SGBXF1_03221 HTH-type transcriptional repressor PurR	GH53
SGBXF1_00958 D-ribose-binding periplasmic protein precursor	GH53
SGBXF1_04176 Catabolite repressor/activator	GH53
SGBXF1_01119 putative diguanylate cyclase AdrA	GH53
SGBXF1_01698 Response regulator PleD	GH53
SGBXF1_04629 Autoinducer 2-binding protein LsrB precursor	GH53
SGBXF1_00612 putative diguanylate cyclase YfiN	GH53
SGBXF1_03353 HTH-type transcriptional regulator GntR	GH53
SGBXF1_02099 HTH-type transcriptional repressor CytR	GH53
SGBXF1_00005 D-ribose-binding periplasmic protein precursor	GH53
SGBXF1_03144 HTH-type transcriptional repressor CytR	GH53
SGBXF1_02533 putative diguanylate cyclase YcdT	GH53
SGBXF1_03264 Diguanylate cyclase DosC	GH53
SGBXF1_00683 Catabolite repressor/activator	GH53
SGBXF1_00163 Phytochrome-like protein cph2	GH53
SGBXF1_03613 putative diguanylate cyclase YegE	GH53
SGBXF1_01998 HTH-type transcriptional regulator LacR	GH53
SGBXF1_01983 D-galactose-binding periplasmic protein precursor	GH53
SGBXF1_04532 HTH-type transcriptional regulator GalS	GH53
SGBXF1_04412 Catabolite control protein A	GH53
SGBXF1_04139 Phytochrome-like protein cph2	GH53
SGBXF1_02300 Maltose regulon regulatory protein MalI	GH53
SGBXF1_02220 HTH-type transcriptional repressor PurR	GH53
SGBXF1_03086 Phytochrome-like protein cph2	GH53
SGBXF1_00066 HTH-type transcriptional regulator KdgR	GH53
SGBXF1_03684 HTH-type transcriptional regulator GalS	GH53

SGBXF1_02278 L-arabinose-binding periplasmic protein precursor	GH53
SGBXF1_04653 Cyclic di-GMP phosphodiesterase Gmr	GH53
SGBXF1_02436 Cyclic di-GMP phosphodiesterase Gmr	GH53
SGBXF1_00104 Xylose operon regulatory protein	GH53/GH39
SGBXF1_02445 putative diguanylate cyclase YegE	GH53/GT5
SGBXF1_03037 Cupin superfamily protein	GH55
SGBXF1_02144 2-deoxyglucose-6-phosphate phosphatase	GH65
SGBXF1_04772 Alpha-D-glucose-1-phosphate phosphatase YihX	GH65
SGBXF1_00036 6-phosphogluconate phosphatase	GH65
SGBXF1_04540 Phosphoglycolate phosphatase	GH65
SGBXF1_00604 Phosphoserine phosphatase	GH65
SGBXF1_00200 Flavin mononucleotide phosphatase YigB	GH65
SGBXF1_03378 Sugar phosphatase YfbT	GH65
SGBXF1_01070 Copper-exporting P-type ATPase A	GH65
SGBXF1_04554 GMP/IMP nucleotidase YrfG	GH65
SGBXF1_01158 Ribonucleotide monophosphatase NagD	GH65
SGBXF1_01577 ?-D-glucose-1-phosphatase	GH65
SGBXF1_00478 Magnesium-transporting ATPase, P-type 1	GH65
SGBXF1_03062 Magnesium-transporting ATPase, P-type 1	GH65
SGBXF1_00866 Enolase-phosphatase E1	GH65
SGBXF1_02141 Phosphonoacetaldehyde hydrolase	GH65
SGBXF1_00784 Fructose-1-phosphate phosphatase YqaB	GH65
SGBXF1_01181 Potassium-transporting ATPase B chain	GH65
SGBXF1_00222 Lead, cadmium, zinc and mercury-transporting ATPase	GH65
SGBXF1_04565 Ferrous iron transport protein B	GH72
SGBXF1_03673 GTPase Der	GH72
SGBXF1_00043 tRNA modification GTPase MnmE	GH72
SGBXF1_00374 GTPase HflX	GH72
SGBXF1_03733 GTPase Era	GH72
SGBXF1_02011 Ribosome-binding ATPase YchF	GH72
SGBXF1_00418 GTPase ObgE/CgtA	GH72
SGBXF1_04780 putative GTP-binding protein EngB	GH72
SGBXF1_01924 putative aminodeoxychorismate lyase	GH73
SGBXF1_02995 Peptidoglycan hydrolase FlgJ	GH73
SGBXF1_03630 putative zinc-type alcohol dehydrogenase-like protein YjmD	GH76
SGBXF1_04397 Quinone oxidoreductase 1	GH76
SGBXF1_01350 Quinone oxidoreductase 1	GH76
SGBXF1_04388 Aldehyde reductase YahK	GH76
SGBXF1_03351 L-idonate 5-dehydrogenase (NAD(P)(+))	GH76
SGBXF1_01978 Aryl-alcohol dehydrogenase	GH76
SGBXF1_04183 Alcohol dehydrogenase	GH76
SGBXF1_02660 Zinc-type alcohol dehydrogenase-like protein	GH76
SGBXF1_02428 Alcohol dehydrogenase 1	GH76
SGBXF1_04730 L-threonine 3-dehydrogenase	GH76
SGBXF1_01521 S-(hydroxymethyl)glutathione dehydrogenase	GH76
SGBXF1_03026 NADPH-dependent curcumin reductase	GH76
SGBXF1_03075 Phthiocerol synthesis polyketide synthase type I PpsC	GH76
SGBXF1_04571 4-alpha-glucanotransferase	GH77
SGBXF1_00162 Cellulose synthase operon protein C precursor	GH8
SGBXF1_00161 Endoglucanase precursor	GH8
SGBXF1_03039 putative peptidase	GH84
SGBXF1_03901 Methionine aminopeptidase	GH84
SGBXF1_00447 Methionine aminopeptidase 1, mitochondrial	GH84
SGBXF1_01315 Methionine aminopeptidase	GH84
SGBXF1_00281 Xaa-Pro dipeptidase	GH84
SGBXF1_04019 Xaa-Pro aminopeptidase	GH84
SGBXF1_02026 hypothetical protein	GH9
SGBXF1_02509 hypothetical protein	GH9
SGBXF1_00557 D-beta-hydroxybutyrate dehydrogenase	GH92
SGBXF1_02079 Cyclopentanol dehydrogenase	GH92
SGBXF1_01410 3-oxoacyl-[acyl-carrier-protein] reductase FabG	GH92
SGBXF1_02323 NADP-dependent 3-hydroxy acid dehydrogenase YdfG	GH92
SGBXF1_01393 3-oxoacyl-[acyl-carrier-protein] reductase FabG	GH92
SGBXF1_04382 Cyclic-di-GMP-binding biofilm dispersal mediator protein	GH92
SGBXF1_00826 3-oxoacyl-[acyl-carrier-protein] reductase FabG	GH92
SGBXF1_03638 Sorbitol-6-phosphate 2-dehydrogenase	GH92
SGBXF1_01355 Acetoacetyl-CoA reductase	GH92
SGBXF1_03494 2,3-dihydro-2,3-dihydroxybenzoate dehydrogenase	GH92

SGBXF1_03052 2-(R)-hydroxypropyl-CoM dehydrogenase	GH92
SGBXF1_00579 2,5-dichloro-2,5-cyclohexadiene-1,4-diol dehydrogenase	GH92
SGBXF1_00767 Glucose 1-dehydrogenase 1	GH92
SGBXF1_00136 3-oxoacyl-[acyl-carrier-protein] reductase FabG	GH92
SGBXF1_03834 3-oxoacyl-[acyl-carrier-protein] reductase FabG	GH92
SGBXF1_02710 putative oxidoreductase YciK	GH92
SGBXF1_04057 7-alpha-hydroxysteroid dehydrogenase	GH92
SGBXF1_00559 (S)-1-Phenylethanol dehydrogenase	GH92
SGBXF1_03083 Glucose 1-dehydrogenase	GH92
SGBXF1_02440 C-factor	GH92
SGBXF1_00575 2-keto-3-deoxy-L-fuconate dehydrogenase	GH92
SGBXF1_03352 Gluconate 5-dehydrogenase	GH92
SGBXF1_01934 3-oxoacyl-[acyl-carrier-protein] reductase FabG	GH92
SGBXF1_01920 3-oxoacyl-[acyl-carrier-protein] reductase FabG	GH92
SGBXF1_04064 Pyridoxal 4-dehydrogenase	GH92
SGBXF1_04007 3-oxoacyl-[acyl-carrier-protein] reductase FabG	GH92
SGBXF1_04437 Acetate operon repressor	GH93
SGBXF1_02138 Pectin degradation repressor protein KdgR	GH93
SGBXF1_03041 Pca regulon regulatory protein	GH93
SGBXF1_02528 Pca regulon regulatory protein	GH93
SGBXF1_00949 Transcriptional regulator KdgR	GH93
SGBXF1_01276 HTH-type transcriptional regulator SrpS	GH93
SGBXF1_04063 Pca regulon regulatory protein	GH93
SGBXF1_04028 HTH-type transcriptional regulator YiaJ	GH93
SGBXF1_04761 Guanylate kinase	GH99
SGBXF1_03887 Lipid-A-disaccharide synthase	GT19
SGBXF1_04088 Sensor protein QseC	GT2
SGBXF1_00955 Blue-light-activated protein	GT2
SGBXF1_04287 Aerobic respiration control sensor protein ArcB	GT2
SGBXF1_01057 Chaperone protein HtpG	GT2
SGBXF1_03440 Phosphohistidine phosphatase SixA	GT2
SGBXF1_02039 Virulence sensor histidine kinase PhoQ	GT2
SGBXF1_01714 3-oxoacyl-[acyl-carrier-protein] synthase 2	GT2
SGBXF1_00819 Undecaprenyl-phosphate mannosyltransferase	GT2
SGBXF1_04524 Acetylornithine/succinyldiaminopimelate aminotransferase	GT2
SGBXF1_00160 Cyclic di-GMP-binding protein precursor	GT2
SGBXF1_02558 Sensor protein QseC	GT2
SGBXF1_03525 Sensor protein CpxA	GT2
SGBXF1_03431 3-oxoacyl-[acyl-carrier-protein] synthase 1	GT2
SGBXF1_03718 Sensor histidine kinase GlrK	GT2
SGBXF1_00824 3-oxoacyl-[acyl-carrier-protein] synthase 2	GT2
SGBXF1_01922 3-oxoacyl-[acyl-carrier-protein] synthase 2	GT2
SGBXF1_01289 hypothetical protein	GT2
SGBXF1_00827 3-oxoacyl-[acyl-carrier-protein] synthase 2	GT2
SGBXF1_00159 Cellulose synthase catalytic subunit [UDP-forming]	GT2
SGBXF1_02856 putative glycosyltransferase EpsJ	GT2
SGBXF1_03838 Sensor histidine kinase CitA	GT2
SGBXF1_01087 Cysteine--tRNA ligase	GT2
SGBXF1_02176 Undecaprenyl-phosphate 4-deoxy-4-formamido-L-arabinose transferase	GT2
SGBXF1_01251 Adenosylmethionine-8-amino-7-oxononanoate aminotransferase	GT2
SGBXF1_01227 2,3-bisphosphoglycerate-dependent phosphoglycerate mutase	GT2
SGBXF1_03657 Sensor protein TorS	GT2
SGBXF1_01465 Sensor protein CzcS precursor	GT2
SGBXF1_01562 Putative O-antigen transporter	GT2
SGBXF1_03019 Chemotaxis protein CheA	GT2
SGBXF1_00419 Sensor protein BasS	GT2
SGBXF1_02536 hypothetical protein	GT2
SGBXF1_04775 Nitrogen regulation protein NR(II)	GT2
SGBXF1_04718 Sensor protein CpxA	GT2
SGBXF1_04255 4-aminobutyrate aminotransferase PuuE	GT2
SGBXF1_04737 putative glycosyltransferase EpsJ	GT2
SGBXF1_00985 Tetrathionate sensor histidine kinase TtrS	GT2
SGBXF1_00715 Glutamate-1-semialdehyde 2,1-aminomutase	GT2
SGBXF1_03472 Sensor histidine kinase YpdA	GT2
SGBXF1_04011 Swarming motility regulation sensor protein RssA	GT2
SGBXF1_03988 2'-deamino-2'-hydroxyneamine transaminase	GT2
SGBXF1_01179 Sensor protein KdpD	GT2
SGBXF1_00542 Omega-amino acid--pyruvate aminotransferase	GT2
SGBXF1_02927 putative glycosyltransferase EpsJ	GT2

SGBXF1_03331 Phosphotransferase RcsD	GT2
SGBXF1_00051 DNA gyrase subunit B	GT2
SGBXF1_02444 Diaminobutyrate--2-oxoglutarate aminotransferase	GT2
SGBXF1_03999 Sensor protein CreC	GT2
SGBXF1_00263 Alpha-ribazole phosphatase	GT2
SGBXF1_00968 Phosphate regulon sensor protein PhoR	GT2
SGBXF1_00724 Signal transduction histidine-protein kinase BarA	GT2
SGBXF1_02621 Sensor protein RstB	GT2
SGBXF1_04370 Lipid A biosynthesis lauroyl acyltransferase	GT2
SGBXF1_01919 Malonyl CoA-acyl carrier protein transacylase	GT2
SGBXF1_01578 putative glycosyl transferase	GT2
SGBXF1_02926 putative glycosyltransferase EpsJ	GT2
SGBXF1_04560 Osmolarity sensor protein EnvZ	GT2
SGBXF1_03624 Signal transduction histidine-protein kinase BaeS	GT2
SGBXF1_02940 Nitrate/nitrite sensor protein NarX	GT2
SGBXF1_02820 Lipid A biosynthesis (KDO)2-(lauroyl)-lipid IVA acyltransferase	GT2
SGBXF1_03404 Sensor protein QseC	GT2
SGBXF1_04646 Autoinducer 2 sensor kinase/phosphatase LuxQ	GT2
SGBXF1_02933 colanic acid exporter	GT2
SGBXF1_02929 Putative teichuronic acid biosynthesis glycosyltransferase TuaG	GT2
SGBXF1_03564 Nitrate/nitrite sensor protein NarX	GT2
SGBXF1_03333 Sensor histidine kinase RcsC	GT2
SGBXF1_04745 putative glycosyl transferase	GT2
SGBXF1_02932 hypothetical protein	GT2
SGBXF1_01473 putative sensor histidine kinase TcrY	GT2
SGBXF1_02749 UDP-glucose 6-dehydrogenase TuaD	GT2
SGBXF1_00175 UDP-N-acetyl-D-glucosamine 6-dehydrogenase	GT2
SGBXF1_02931 Undecaprenyl-phosphate 4-deoxy-4-formamido-L-arabinose transferase	GT2
SGBXF1_01898 Lipid A biosynthesis lauroyl acyltransferase	GT2
SGBXF1_02895 Succinylornithine transaminase	GT2
SGBXF1_00617 Phosphoserine phosphatase 1	GT2
SGBXF1_01981 Sensor histidine kinase DcuS	GT2
SGBXF1_01579 N-acetylglucosaminyl-diphospho-decaprenol L-rhamnosyltransferase	GT2
SGBXF1_04209 DNA topoisomerase 4 subunit B	GT2
SGBXF1_01885 Glucans biosynthesis glucosyltransferase H	GT2
SGBXF1_02064 Phthioceranic/hydroxyphthioceranic acid synthase	GT2/GH76/ GT4
SGBXF1_02217 Cyclopropane-fatty-acyl-phospholipid synthase	GT2/GT4
SGBXF1_01075 Cyclopropane-fatty-acyl-phospholipid synthase	GT2/GT4
SGBXF1_00525 Alpha,alpha-trehalose-phosphate synthase [UDP-forming]	GT20
SGBXF1_02726 putative sulfate transporter/MT1781	GT26
SGBXF1_00183 UDP-N-acetyl-D-mannosaminuronic acid transferase	GT26
SGBXF1_04302 putative phospholipid ABC transporter-binding protein MlaB	GT26
SGBXF1_00689 UDP-N-acetylmuramoyl-L-alanyl-D-glutamate--2,6-diaminopimelate ligase	GT28
SGBXF1_00690 UDP-N-acetylmuramoyl-tripeptide--D-alanyl-D-alanine ligase	GT28
SGBXF1_01127 Rod shape-determining protein RodA	GT28
SGBXF1_00407 UDP-N-acetylmuramate:L-alanyl-gamma-D-glutamyl-meso-diaminopimelate ligase	GT28
SGBXF1_00692 UDP-N-acetylmuramoylalanine--D-glutamate ligase	GT28
SGBXF1_00694 UDP-N-acetylglucosamine--N-acetylmuramyl-(pentapeptide) pyrophosphoryl-undecaprenol N-acetylglucosamine transferase	GT28
SGBXF1_00693 Lipid II flippase FtsW	GT28
SGBXF1_00695 UDP-N-acetylmuramate--L-alanine ligase	GT28
SGBXF1_00174 UDP-N-acetylglucosamine 2-epimerase	GT28
SGBXF1_04135 tRNA (guanine-N(7)-)-methyltransferase	GT30
SGBXF1_01581 6-phosphogluconate dehydrogenase, decarboxylating	GT30
SGBXF1_01678 Tetraacyldisaccharide 4'-kinase	GT30
SGBXF1_04744 3-deoxy-D-manno-octulosonic acid transferase	GT30
SGBXF1_00980 Queuine tRNA-ribosyltransferase	GT34
SGBXF1_04579 Maltodextrin phosphorylase	GT35
SGBXF1_04572 Maltodextrin phosphorylase	GT35
SGBXF1_03674 Outer membrane protein assembly factor BamB precursor	GT39
SGBXF1_00528 Outer membrane protein assembly factor BamB	GT39
SGBXF1_02817 putative HTH-type transcriptional regulator YbbH	GT4
SGBXF1_03166 Inner membrane transport permease YbhS	GT4
SGBXF1_00026 Bifunctional protein GlmU	GT4
SGBXF1_04581 Glucose-1-phosphate adenylyltransferase	GT4
SGBXF1_00813 2-acyl-glycerophospho-ethanolamine acyltransferase	GT4
SGBXF1_03706 hypothetical protein	GT4
SGBXF1_02597 2-acyl-glycerophospho-ethanolamine acyltransferase	GT4

SGBXF1_01548 UTP--glucose-1-phosphate uridylyltransferase	GT4
SGBXF1_04372 Tyrocidine synthase 3	GT4
SGBXF1_04094 Inner membrane transport permease YadH	GT4
SGBXF1_04407 Diacylglycerol kinase	GT4
SGBXF1_01552 Glycosyl transferases group 1	GT4
SGBXF1_00081 putative methyltransferase YcgJ	GT4
SGBXF1_00777 Regulatory protein RecX	GT4
SGBXF1_04283 DnaA initiator-associating protein DiaA	GT4
SGBXF1_00266 Ubiquinone/menaquinone biosynthesis C-methyltransferase UbiE	GT4
SGBXF1_00211 Phospholipase YtpA	GT4
SGBXF1_01268 Inner membrane transport permease YbhR	GT4
SGBXF1_03807 Malonyl-[acyl-carrier protein] O-methyltransferase	GT4
SGBXF1_03045 3-oxoadipate enol-lactonase 2	GT4
SGBXF1_04741 Lipopolysaccharide core biosynthesis protein RfaG	GT4
SGBXF1_00871 Phosphoheptose isomerase	GT4
SGBXF1_00935 putative methyltransferase YcgJ	GT4
SGBXF1_01750 Putative aminoacrylate hydrolase RutD	GT4
SGBXF1_01569 Glucose-1-phosphate thymidylyltransferase 2	GT4
SGBXF1_01269 Inner membrane transport permease YbhS	GT4
SGBXF1_01962 Proline iminopeptidase	GT4
SGBXF1_02346 N-formylmaleamate deformylase	GT4
SGBXF1_00177 Glucose-1-phosphate thymidylyltransferase 2	GT4
SGBXF1_04568 Pimeloyl-[acyl-carrier protein] methyl ester esterase	GT4
SGBXF1_01554 D-inositol 3-phosphate glycosyltransferase	GT4
SGBXF1_01953 putative phosphatase YcdX	GT4
SGBXF1_02930 GalNAc-alpha-(1->4)-GalNAc-alpha-(1->3)-diNAcBac-PP-undecaprenol alpha-1,4-N-acetyl-D-galactosaminyltransferase	GT4
SGBXF1_04742 N-acetylgalactosamine-N,N'-diacetylbacillosaminyl-diphospho-undecaprenol 4-alpha-N-acetylgalactosaminyltransferase	GT4
SGBXF1_03885 DNA polymerase III subunit alpha	GT4
SGBXF1_02750 UTP--glucose-1-phosphate uridylyltransferase	GT4
SGBXF1_04515 putative hydrolase	GT4
SGBXF1_03165 Inner membrane transport permease YbhR	GT4
SGBXF1_04408 Glycerol-3-phosphate acyltransferase	GT4
SGBXF1_00403 Gamma-glutamylcyclotransferase family protein YtfP	GT4
SGBXF1_01575 Teichoic acid translocation permease protein TagG	GT4
SGBXF1_02367 Haloalkane dehalogenase	GT4
SGBXF1_01173 Esterase YbfF	GT4
SGBXF1_03503 Enterobactin synthase component F	GT4
SGBXF1_00549 Phospholipase YtpA	GT4
SGBXF1_02835 tRNA (mo5U34)-methyltransferase	GT4
SGBXF1_00916 Soluble epoxide hydrolase	GT4
SGBXF1_04204 1-acyl-sn-glycerol-3-phosphate acyltransferase	GT4
SGBXF1_01565 Mannose-1-phosphate guanylyltransferase 1	GT4
SGBXF1_02569 Soluble epoxide hydrolase	GT4
SGBXF1_03944 Bifunctional protein Aas	GT4
SGBXF1_01563 UDP-galactopyranose mutase	GT4
SGBXF1_01547 UTP--glucose-1-phosphate uridylyltransferase	GT4
SGBXF1_02074 Pentapeptide repeats (8 copies)	GT4
SGBXF1_03175 N-acyltransferase YncA	GT41
SGBXF1_03743 tRNA1(Val) (adenine(37)-N6)-methyltransferase	GT41
SGBXF1_00591 Mycothiol acetyltransferase	GT41
SGBXF1_00068 putative N-acetyltransferase YjaB	GT41
SGBXF1_01418 Mycothiol acetyltransferase	GT41
SGBXF1_01595 putative acetyltransferase	GT41
SGBXF1_00703 Protein translocase subunit SecA	GT41
SGBXF1_03855 Acetyltransferase (GNAT) family protein	GT41
SGBXF1_01310 TDP-fucosamine acetyltransferase	GT41
SGBXF1_02492 Acetyltransferase (GNAT) family protein	GT41
SGBXF1_03646 putative acetyltransferase	GT41
SGBXF1_00589 Ribosomal RNA small subunit methyltransferase C	GT41
SGBXF1_00178 dTDP-fucosamine acetyltransferase	GT41
SGBXF1_03808 Acetyltransferase (GNAT) family protein	GT41
SGBXF1_03983 Spermine/spermidine acetyltransferase	GT41
SGBXF1_00913 putative N-acetyltransferase YycN	GT41
SGBXF1_01381 Protease synthase and sporulation negative regulatory protein PAI 1	GT41
SGBXF1_04574 putative acetyltransferase	GT41
SGBXF1_02042 Acetyltransferase (GNAT) family protein	GT41
SGBXF1_01304 Phosphinothricin N-acetyltransferase	GT41

SGBXF1_00941 Acetyltransferase (GNAT) family protein	GT41
SGBXF1_01341 MarR family protein	GT41
SGBXF1_04259 Ribosomal RNA large subunit methyltransferase G	GT41
SGBXF1_02534 Acetyltransferase (GNAT) family protein	GT41
SGBXF1_00506 Acetyltransferase (GNAT) family protein	GT41
SGBXF1_03538 Acetyltransferase YpeA	GT41
SGBXF1_04155 putative acetyltransferase	GT41
SGBXF1_01439 Mycothiol acetyltransferase	GT41
SGBXF1_03438 50S ribosomal protein L3 glutamine methyltransferase	GT41
SGBXF1_00301 Acetyltransferase (GNAT) family protein	GT41
SGBXF1_01416 Acetyltransferase	GT41
SGBXF1_04769 N-acetylglutamate synthase	GT41
SGBXF1_01968 Acetyltransferase (GNAT) family protein	GT41/CBM 26
SGBXF1_03604 Spermidine N(1)-acetyltransferase	GT41/CBM 26
SGBXF1_00378 Adenylosuccinate synthetase	GT47
SGBXF1_01837 Purine efflux pump PbuE	GT48
SGBXF1_04042 4-hydroxybenzoate transporter PcaK	GT48
SGBXF1_03253 Low-affinity putrescine importer PlaP	GT48
SGBXF1_04519 putative galactarate transporter	GT48
SGBXF1_04267 Hexuronate transporter	GT48
SGBXF1_00736 Proline-specific permease ProY	GT48
SGBXF1_02845 Multidrug resistance protein MdtH	GT48
SGBXF1_02734 Methyl viologen resistance protein SmvA	GT48
SGBXF1_02001 Inner membrane transporter YgjI	GT48
SGBXF1_00739 4-hydroxybenzoate transporter PcaK	GT48
SGBXF1_03858 Alpha-ketoglutarate permease	GT48
SGBXF1_01841 Aspartate-proton symporter	GT48
SGBXF1_00002 putative transport protein HsrA	GT48
SGBXF1_04656 Inner membrane metabolite transport protein YhjE	GT48
SGBXF1_02314 Inner membrane transport protein YnfM	GT48
SGBXF1_02494 D-serine/D-alanine/glycine transporter	GT48
SGBXF1_02791 Multidrug resistance protein MdtL	GT48
SGBXF1_04060 putative sulfoacetate transporter SauU	GT48
SGBXF1_02303 Inner membrane transport protein YdhP	GT48
SGBXF1_01537 Multidrug resistance protein MdtH	GT48
SGBXF1_02086 Putrescine importer PuuP	GT48
SGBXF1_03631 Hexuronate transporter	GT48
SGBXF1_01167 Sugar efflux transporter A	GT48
SGBXF1_04035 Putative tartrate transporter	GT48
SGBXF1_03291 Bicyclomycin resistance protein	GT48
SGBXF1_02515 Putative tartrate transporter	GT48
SGBXF1_00063 Putative tartrate transporter	GT48
SGBXF1_01010 muropeptide transporter	GT48
SGBXF1_00497 Arginine/ornithine antiporter	GT48
SGBXF1_04670 Putative sialic acid transporter	GT48
SGBXF1_04533 Protein TsgA	GT48
SGBXF1_01292 D-serine/D-alanine/glycine transporter	GT48
SGBXF1_00233 putative sulfoacetate transporter SauU	GT48
SGBXF1_00581 L-fucose-proton symporter	GT48
SGBXF1_02867 Multidrug resistance protein stp	GT48
SGBXF1_02402 Purine ribonucleoside efflux pump NepI	GT48
SGBXF1_03908 Putative niacin/nicotinamide transporter NaiP	GT48
SGBXF1_04600 D-xylose-proton symporter	GT48
SGBXF1_02395 Multidrug resistance protein stp	GT48
SGBXF1_01756 D-serine/D-alanine/glycine transporter	GT48
SGBXF1_00555 putative amino acid permease YhdG	GT48
SGBXF1_00119 Reverse transcriptase (RNA-dependent DNA polymerase)	GT48
SGBXF1_02874 Aromatic amino acid transport protein AroP	GT48
SGBXF1_03215 Putative multidrug resistance protein MdtD	GT48
SGBXF1_02325 putative transport protein HsrA	GT48
SGBXF1_03623 Putative multidrug resistance protein MdtD	GT48
SGBXF1_00673 Sugar efflux transporter A	GT48
SGBXF1_02617 Putative arginine/ornithine antiporter	GT48
SGBXF1_00453 Putrescine-ornithine antiporter	GT48
SGBXF1_01873 Proline/betaine transporter	GT48
SGBXF1_04707 Multidrug resistance protein D	GT48
SGBXF1_00071 Hexuronate transporter	GT48

SGBXF1_03990 Multidrug resistance protein MdtL	GT48
SGBXF1_02502 Multidrug resistance protein 3	GT48
SGBXF1_02319 Multidrug resistance protein 3	GT48
SGBXF1_01430 Multidrug resistance protein stp	GT48
SGBXF1_01664 putative MFS-type transporter YcaD	GT48
SGBXF1_03420 putative transporter	GT48
SGBXF1_04068 putative sulfoacetate transporter SauU	GT48
SGBXF1_04411 Proline/betaine transporter	GT48
SGBXF1_03845 Inner membrane transport protein YnfM	GT48
SGBXF1_01275 putative multidrug resistance protein EmrY	GT48
SGBXF1_02106 Proline/betaine transporter	GT48
SGBXF1_03821 putative transport protein HsrA	GT48
SGBXF1_04111 Aromatic amino acid transport protein AroP	GT48
SGBXF1_00146 Regulatory protein UhpC	GT48
SGBXF1_03667 Hexuronate transporter	GT48
SGBXF1_01896 Tetracycline resistance protein, class B	GT48
SGBXF1_03851 Multidrug export protein EmrB	GT48
SGBXF1_02525 4-hydroxybenzoate transporter PcaK	GT48
SGBXF1_02218 Inner membrane transport protein YdhC	GT48
SGBXF1_01065 Fosmidomycin resistance protein	GT48
SGBXF1_03951 Inner membrane transport protein YdhP	GT48
SGBXF1_03647 Inner membrane protein YihN	GT48
SGBXF1_00095 Multidrug transporter MdfA	GT48
SGBXF1_03546 Amino-acid permease RocC	GT48
SGBXF1_02339 Putative tartrate transporter	GT48
SGBXF1_01414 putative multidrug-efflux transporter/MT1670	GT48
SGBXF1_03105 putative transport protein YifK	GT48
SGBXF1_03275 Sugar efflux transporter B	GT48
SGBXF1_01004 Inner membrane transport protein YajR	GT48
SGBXF1_03877 putative cadaverine/lysine antiporter	GT48
SGBXF1_03214 Multidrug resistance protein stp	GT48
SGBXF1_00774 putative MFS-type transporter YcaD	GT48
SGBXF1_00540 putative amino acid permease YhdG	GT48
SGBXF1_00184 putative transport protein YifK	GT48
SGBXF1_00280 Inner membrane transport protein YdhC	GT48
SGBXF1_02526 4-hydroxybenzoate transporter PcaK	GT48
SGBXF1_03218 Sugar efflux transporter B	GT48
SGBXF1_00569 Putative tartrate transporter	GT48
SGBXF1_01330 Purine ribonucleoside efflux pump NepI	GT48
SGBXF1_00830 Inner membrane transport protein YdhP	GT48
SGBXF1_00971 Proline-specific permease ProY	GT48
SGBXF1_03970 Hexuronate transporter	GT48
SGBXF1_02137 Multidrug resistance protein stp	GT48
SGBXF1_02343 Putative metabolite transport protein NicT	GT48
SGBXF1_02376 Tetracycline resistance protein, class C	GT48
SGBXF1_00214 Glycerol-3-phosphate transporter	GT48
SGBXF1_03265 Lysine-specific permease	GT48
SGBXF1_04234 Inner membrane protein YjeH	GT48
SGBXF1_01426 Multidrug resistance protein stp	GT48
SGBXF1_00629 Proline/betaine transporter	GT48
SGBXF1_01834 Inner membrane transport protein RhmT	GT48
SGBXF1_03475 putative MFS-type transporter YhjX	GT48
SGBXF1_01081 Galactose-proton symporter	GT48
SGBXF1_03217 Inner membrane metabolite transport protein YdjE	GT48
SGBXF1_00329 Proton glutamate symport protein	GT49
SGBXF1_04264 Serine/threonine transporter SstT	GT49
SGBXF1_02142 L-cystine uptake protein TcyP	GT49
SGBXF1_00164 Aerobic C4-dicarboxylate transport protein	GT49
SGBXF1_04580 Glycogen synthase	GT5
SGBXF1_02464 Formate hydrogenlyase transcriptional activator	GT5
SGBXF1_01019 ComE operon protein 1	GT5
SGBXF1_03937 Phosphoenolpyruvate-protein phosphotransferase	GT5
SGBXF1_04563 30S ribosomal protein S1	GT5
SGBXF1_04080 Penicillin-binding protein 1B	GT51
SGBXF1_02389 Stage V sporulation protein D	GT51
SGBXF1_02390 Peptidoglycan synthase FtsI precursor	GT51
SGBXF1_04551 Penicillin-binding protein 1A	GT51
SGBXF1_04285 Penicillin-binding protein 2D	GT51
SGBXF1_01128 Stage V sporulation protein D	GT51

SGBXF1_00688 Peptidoglycan synthase FtsI precursor	GT51
SGBXF1_03682 Penicillin-binding protein 1F	GT51
SGBXF1_02586 Zinc transport protein ZntB	GT55
SGBXF1_02654 Zinc transport protein ZntB	GT55
SGBXF1_01594 Magnesium transport protein CorA	GT55
SGBXF1_00203 Magnesium transport protein CorA	GT55
SGBXF1_00181 4-alpha-L-fucosyltransferase	GT56
SGBXF1_00312 Endonuclease V	GT57
SGBXF1_01291 hypothetical protein	GT60
SGBXF1_04529 Peptidyl-prolyl cis-trans isomerase A precursor	GT66
SGBXF1_01086 Peptidyl-prolyl cis-trans isomerase B	GT66
SGBXF1_00032 Phosphate-specific transport system accessory protein PhoU	GT80
SGBXF1_01684 3-deoxy-manno-octulosonate cytidylyltransferase	GT82
SGBXF1_01095 hypothetical protein	GT83
SGBXF1_00908 Major phosphate-irrepressible acid phosphatase precursor	GT83
SGBXF1_01524 Major phosphate-irrepressible acid phosphatase precursor	GT83
SGBXF1_00607 hypothetical protein	GT83
SGBXF1_02702 Phosphatidylglycerophosphatase B	GT83
SGBXF1_03349 N,N'-diacetylchitobiose phosphorylase	GT84/GH94
SGBXF1_04213 ADP-ribose pyrophosphatase	GT87
SGBXF1_02858 putative Nudix hydrolase NudL	GT87
SGBXF1_02227 Isopentenyl-diphosphate Delta-isomerase	GT87
SGBXF1_04552 ADP compounds hydrolase NudE	GT87
SGBXF1_02047 Phosphatase NudJ	GT87
SGBXF1_03938 RNA pyrophosphohydrolase	GT87
SGBXF1_00310 NADH pyrophosphatase	GT87
SGBXF1_03558 GDP-mannose pyrophosphatase NudK	GT87
SGBXF1_03383 putative Nudix hydrolase YfcD	GT87
SGBXF1_03272 RNA pyrophosphohydrolase	GT87
SGBXF1_01403 Nucleoside triphosphatase NudI	GT87
SGBXF1_02761 CTP pyrophosphohydrolase	GT87
SGBXF1_02829 Dihydroneopterin triphosphate pyrophosphatase	GT87
SGBXF1_00704 8-oxo-dGTP diphosphatase	GT87
SGBXF1_00510 Lipopolysaccharide export system permease protein LptF	GT9
SGBXF1_04740 Lipopolysaccharide core heptosyltransferase RfaQ	GT9
SGBXF1_04743 Lipopolysaccharide core heptosyltransferase RfaQ	GT9
SGBXF1_04734 Lipopolysaccharide heptosyltransferase 1	GT9
SGBXF1_01131 Nicotinate-nucleotide adenylyltransferase	GT9
SGBXF1_00511 Lipopolysaccharide export system permease protein LptG	GT9
SGBXF1_04738 ADP-heptose--LPS heptosyltransferase 2	GT9
SGBXF1_04746 Phosphopantetheine adenylyltransferase	GT9
SGBXF1_04733 ADP-heptose--LPS heptosyltransferase 2	GT9
SGBXF1_03181 Lipopolysaccharide core heptosyltransferase RfaQ	GT9
SGBXF1_00170 Thioredoxin-1	GT90
SGBXF1_03853 Thioredoxin-2	GT90
SGBXF1_01076 Thioredoxin	GT90
SGBXF1_03413 Sporulation thiol-disulfide oxidoreductase A precursor	GT90/GH18

Table S3- Protease families predicted in the Merops database

**Locus tag**	**MEROPS family name**	MEROPS Reference	E value
SGBXF1_00638	A08	MER001313	1.20E-61
SGBXF1_02943	A24A	MER000870	3.90E-20
SGBXF1_02450	A31	MER002085	3.10E-60
SGBXF1_01193	C15	MER001424	9.10E-60
SGBXF1_03027	C15	MER001424	9.70E-51
SGBXF1_00646	C26	MER115185	1.60E-78
SGBXF1_00646	C26	MER436691	3.50E-05
SGBXF1_00727	C26	MER065554	1.30E-141
SGBXF1_01585	C26	MER065588	1.20E-93
SGBXF1_02083	C26	MER031278	1.40E-117
SGBXF1_02593	C26	MER043537	2.70E-05
SGBXF1_02717	C26	MER043392	5.40E-79
SGBXF1_03663	C26	MER045886	9.90E-53
SGBXF1_04262	C26	MER043475	2.90E-14
SGBXF1_04371	C26	MER437468	1.20E-19
SGBXF1_04525	C26	MER043394	4.40E-72
SGBXF1_02182	C40	MER002443	2.30E-56
SGBXF1_02223	C40	MER004054	6.10E-61
SGBXF1_03284	C40	MER004035	8.80E-56
SGBXF1_00027	C44	MER003327	1.90E-102
SGBXF1_01156	C44	MER034539	6.00E-112
SGBXF1_03393	C44	MER011806	9.00E-42
SGBXF1_04289	C44	MER198917	1.80E-82
SGBXF1_01001	C56	MER010992	6.00E-14
SGBXF1_01280	C56	MER031431	5.10E-15
SGBXF1_02497	C56	MER014721	4.30E-51
SGBXF1_03719	C56	MER042827	3.70E-23
SGBXF1_04286	C56	MER160094	1.00E-18
SGBXF1_00532	C82	MER107825	8.70E-71
SGBXF1_00873	C82	MER152590	7.40E-51
SGBXF1_02207	C82	MER107808	8.60E-52
SGBXF1_01612	I11	MER018254	2.60E-57
SGBXF1_02216	I13	MER029359	9.00E-05
SGBXF1_00224	I38	MER018233	5.30E-43
SGBXF1_02364	I38	MER018231	2.50E-15
SGBXF1_03683	I39	MER034541	0.00E+00
SGBXF1_01247	I51	MER028934	2.10E-10
SGBXF1_02944	I51	MER028934	8.20E-08
SGBXF1_02116	I78	MER059865	7.90E-06
SGBXF1_00375	I87	MER192051	6.20E-169
SGBXF1_00376	I87	MER191412	2.80E-137
SGBXF1_01074	I87	MER192051	1.80E-12
SGBXF1_01699	M01	MER001001	1.10E-117
SGBXF1_01433	M03A	MER001161	8.80E-07
SGBXF1_01433	M03A	MER001149	2.70E-06
SGBXF1_02341	M03A	MER001158	1.30E-253
SGBXF1_04637	M03A	MER001161	0.00E+00
SGBXF1_03669	M04	MER115298	2.90E-175
SGBXF1_04053	M10A	MER004909	6.10E-05
SGBXF1_00223	M10B	MER001096	7.50E-106
SGBXF1_02114	M10B	MER029131	1.50E-38
SGBXF1_02115	M10B	MER029131	3.40E-05
SGBXF1_02407	M10B	MER001096	7.40E-51
SGBXF1_02661	M14B	MER030306	1.70E-71
SGBXF1_03576	M15B	MER014983	8.50E-05
SGBXF1_03550	M15C	MER030246	8.30E-12
SGBXF1_03770	M15C	MER004957	4.50E-05
SGBXF1_02381	M15D	MER027775	2.30E-20
SGBXF1_03928	M16A	MER001222	4.80E-91
SGBXF1_03928	M16A	MER078753	1.10E-14
SGBXF1_03928	M16A	MER046874	9.90E-08
SGBXF1_00165	M16B	MER001233	4.20E-14
SGBXF1_00509	M17	MER001236	4.50E-269
SGBXF1_02899	M17	MER001236	3.30E-49
SGBXF1_03688	M17	MER002497	1.00E-183

SGBXF1_03575	M20A	MER001272	9.80E-87
SGBXF1_03575	M20A	MER001272	6.30E-46
SGBXF1_04689	M20A	MER001273	4.90E-86
SGBXF1_04689	M20A	MER001273	2.70E-42
SGBXF1_02036	M20B	MER001421	2.80E-188
SGBXF1_03422	M20B	MER001421	8.70E-71
SGBXF1_00884	M20C	MER001283	3.90E-232
SGBXF1_01112	M20D	MER002655	9.40E-100
SGBXF1_03243	M20D	MER002014	1.70E-69
SGBXF1_04163	M20D	MER003581	1.30E-120
SGBXF1_04164	M20D	MER014418	7.00E-14
SGBXF1_04164	M20D	MER014418	1.70E-09
SGBXF1_04518	M20D	MER002655	1.10E-79
SGBXF1_00859	M20X	MER026469	4.80E-48
SGBXF1_02111	M20X	MER026469	2.20E-50
SGBXF1_02583	M20X	MER026469	1.90E-51
SGBXF1_00330	M23B	MER015415	2.70E-25
SGBXF1_00760	M23B	MER015415	1.40E-26
SGBXF1_02821	M23B	MER003380	1.90E-121
SGBXF1_04728	M23B	MER005300	1.50E-40
SGBXF1_01315	M24A	MER001243	5.90E-74
SGBXF1_03901	M24A	MER001243	1.40E-136
SGBXF1_00281	M24B	MER001250	9.20E-132
SGBXF1_03039	M24B	MER004931	2.20E-33
SGBXF1_04019	M24B	MER001244	3.80E-122
SGBXF1_00745	M28C	MER001290	1.20E-129
SGBXF1_02622	M32	MER069021	8.80E-145
SGBXF1_01160	M38	MER033184	1.90E-47
SGBXF1_01910	M38	MER061068	1.00E-143
SGBXF1_02095	M38	MER033186	1.30E-50
SGBXF1_02110	M38	MER015112	2.00E-16
SGBXF1_02335	M38	MER037714	9.20E-43
SGBXF1_00425	M41	MER001620	2.40E-151
SGBXF1_02136	M48B	MER002637	1.60E-99
SGBXF1_03594	M48C	MER031491	2.30E-94
SGBXF1_04053	M48C	MER002639	1.20E-80
SGBXF1_03893	M50B	MER004480	3.30E-87
SGBXF1_03893	M50B	MER004480	3.00E-54
SGBXF1_01367	M60	MER042489	1.00E-113
SGBXF1_03436	M74	MER001298	5.00E-105
SGBXF1_01232	M79	MER056027	1.40E-08
SGBXF1_02688	M79	MER059868	1.70E-09
SGBXF1_03139	M90	MER217243	4.50E-108
SGBXF1_03654	N04	MER187148	2.50E-10
SGBXF1_03011	N06	MER181656	3.70E-120
SGBXF1_00539	P01	MER013629	1.50E-38
SGBXF1_04591	P01	MER035593	1.50E-14
SGBXF1_02088	S01A	MER098939	3.80E-06
SGBXF1_02624	S01A	MER005249	5.50E-83
SGBXF1_00723	S01C	MER001372	1.90E-117
SGBXF1_03893	S01C	MER001372	1.30E-05
SGBXF1_04298	S01C	MER001372	3.40E-142
SGBXF1_04299	S01C	MER001373	1.50E-70
SGBXF1_04648	S08A	MER000329	6.20E-65
SGBXF1_04649	S08A	MER000329	7.60E-58
SGBXF1_04649	S08A	MER000329	6.20E-10
SGBXF1_01956	S09A	MER000410	1.70E-120
SGBXF1_02265	S09A	MER005694	5.60E-68
SGBXF1_03543	S09A	MER000410	4.90E-103
SGBXF1_02477	S09B	MER058228	2.30E-05
SGBXF1_02476	S09C	MER034615	8.10E-07
SGBXF1_02903	S09C	MER043146	2.80E-31
SGBXF1_04210	S09C	MER107796	9.60E-85
SGBXF1_00386	S09X	MER034550	1.50E-68
SGBXF1_01440	S09X	MER037863	3.80E-06
SGBXF1_01520	S09X	MER061081	5.00E-122
SGBXF1_02394	S09X	MER031563	2.80E-31
SGBXF1_02568	S09X	MER033237	5.00E-23
SGBXF1_02568	S09X	MER034961	1.30E-06

SGBXF1_03108	S09X	MER031563	4.30E-32
SGBXF1_03578	S09X	MER065576	7.00E-26
SGBXF1_01125	S11	MER000450	3.00E-147
SGBXF1_01300	S11	MER000455	5.30E-130
SGBXF1_01603	S11	MER000450	1.70E-158
SGBXF1_02589	S11	MER043199	2.80E-65
SGBXF1_02683	S11	MER000451	4.60E-131
SGBXF1_00573	S12	MER004154	1.80E-130
SGBXF1_02093	S12	MER000457	4.20E-12
SGBXF1_02302	S12	MER000463	3.30E-85
SGBXF1_02306	S12	MER006204	3.90E-92
SGBXF1_02342	S12	MER026262	6.40E-20
SGBXF1_00421	S13	MER000472	1.30E-226
SGBXF1_01014	S14	MER000474	9.10E-113
SGBXF1_02008	S14	MER000474	6.60E-71
SGBXF1_02387	S14	MER002299	2.40E-07
SGBXF1_00605	S16	MER014135	4.90E-139
SGBXF1_01016	S16	MER000485	6.20E-133
SGBXF1_01717	S16	MER003018	3.60E-99
SGBXF1_02650	S24	MER000576	6.70E-37
SGBXF1_03236	S24	MER000576	5.50E-28
SGBXF1_03319	S24	MER037220	2.50E-08
SGBXF1_04406	S24	MER000569	1.80E-62
SGBXF1_03735	S26A	MER000589	3.70E-120
SGBXF1_00133	S33	MER036050	1.30E-05
SGBXF1_00211	S33	MER036081	1.80E-09
SGBXF1_00549	S33	MER033247	6.10E-25
SGBXF1_00916	S33	MER004146	3.00E-13
SGBXF1_01173	S33	MER031610	3.50E-42
SGBXF1_01750	S33	MER004146	1.10E-10
SGBXF1_01962	S33	MER000431	1.10E-87
SGBXF1_02346	S33	MER031610	1.00E-10
SGBXF1_02367	S33	MER017177	1.70E-11
SGBXF1_02569	S33	MER031617	4.10E-17
SGBXF1_02782	S33	MER213595	5.80E-08
SGBXF1_03045	S33	MER210990	2.60E-08
SGBXF1_03346	S33	MER034563	4.60E-59
SGBXF1_04058	S33	MER037236	9.20E-10
SGBXF1_04515	S33	MER059846	2.00E-129
SGBXF1_04568	S33	MER017142	1.10E-106
SGBXF1_02134	S41A	MER001295	2.60E-91
SGBXF1_02767	S49A	MER001299	6.20E-241
SGBXF1_02709	S49B	MER001300	8.20E-82
SGBXF1_00275	S51	MER001335	2.30E-39
SGBXF1_04576	S54	MER015468	1.30E-56
SGBXF1_03779	S73	MER150756	6.60E-18
SGBXF1_04699	T01B	MER001627	1.50E-85
SGBXF1_01514	T02	MER003338	4.60E-59
SGBXF1_02902	T02	MER017326	3.20E-28
SGBXF1_00852	T03	MER223926	5.20E-94
SGBXF1_00975	T03	MER001978	3.90E-145
SGBXF1_02507	T03	MER001978	1.30E-105
SGBXF1_00448	U32	MER037246	1.00E-126
SGBXF1_00449	U32	MER013876	1.80E-18
SGBXF1_03627	U32	MER003855	1.60E-44
SGBXF1_03627	U32	MER003855	3.30E-15
SGBXF1_04334	U62	MER016222	4.20E-206
SGBXF1_04350	U62	MER016301	3.60E-222
SGBXF1_01901	U73	MER492485	5.00E-71

Table S4- Genes involved in secretion systems

Locus/operon tag	Gene/operon	Product/putative product	Function/Putative function
SGBXF1_00703	*secA*	Preprotein translocase subunit SecA	Sec secretion system
SGBXF1_00702	*secM*	Secretion monitor	
SGBXF1_04724	*secB*	Preprotein translocase subunit SecB	
SGBXF1_00982	*secD*	Preprotein translocase subunit SecD	
SGBXF1_00983	*secF*	Preprotein translocase subunit SecF	
SGBXF1_00428	*secG*	Protein-export membrane protein	
SGBXF1_04469	*secY*	Preprotein translocase subunit SecY	
SGBXF1_00269-72	*tatABCD*	Sec-independent protein translocase protein	Twin-arginine translocation (Tat) system
SGBXF1_01120	*tatE*	Sec-independent protein translocase protein TatE	
SGBXF1_01559-61	*prsDEF*	Protease secretion system proteins	Protease secretion system

Table S5- Genes involved in metal transport and resistance

Locus tag/operon	Gene/operon	Product/putative product	Function/Putative function
SGBXF1_01070	*copA*	Copper-exporting P-type ATPase A	Copper resistance
SGBXF1_01071	*cueR*	HTH-type transcriptional regulator	
SGBXF1_01965-66	*copCD*	Copper resistance protein	
SGBXF1_04098	*cueO*	Blue copper oxidase	
SGBXF1_03425-28	*cusABCF*	Cation efflux system proteins	Copper and silver resistance
SGBXF1_00222	*zntA*	Lead, cadmium, zinc and mercury-transporting ATPase	Zinc and other metals resistance
SGBXF1_02822-24	*znuCBA*	High-affinity zinc uptake system proteins	
SGBXF1_01223	*zitB*	Zinc transporter	
SGBXF1_02654	*zntB*	Zinc transport protein	
SGBXF1_04231	*zupT*	Zinc transporter	
SGBXF1_01040-42	*znuC, mntB*	Zinc/manganese transport system elements	Zinc and manganese resistance
SGBXF1_00203	*corA*	Magnesium transport protein	Magnesium resistance
SGBXF1_02866	*mntP*	Manganese efflux pump	Manganese resistance
SGBXF1_03699	*rcnA*	Nickel/cobalt efflux protein	Nickel and cobalt resistance
SGBXF1_02446	*hoxN*	High-affinity nickel transport protein	Nickel resistance
SGBXF1_01242-44	*modABC*	Molybdenum transport system	Molybdate resistance
SGBXF1_03053-55	*arsCBR*	Arsenate reductase	Arsenate resistance
SGBXF1_00039	*chrR*	Chromate reductase	Chromate resistance
SGBXF1_00912	*tehB*	Tellurite methyltransferase	Tellurium resistance

Table S6- Genes involved in siderophore production and iron transport

Locus tag/operon	Gene/operon	Product/putative product	Function/Putative function
SGBXF1_00842-46	*iutA, iucABCD*	Aerobactin production operon	Siderophore production
SGBXF1_03494-03506	*entABECF*	Enterobactin production operon	Siderophore production
SGBXF1_00277	*fre*	Ferrisiderophore reductase	Iron release from siderophores
SGBXF1_02729	*tonB*	Siderophore transmembrane transporter	Siderophore and other compounds transport
SGBXF1_02787	*foxA*	Ferrioxamine receptor	Siderophore and iron transport
SGBXF1_03569	*fpvA*	Ferripyoverdine receptor	Siderophore and iron transport
SGBXF1_02326	*fecA*	$Fe^{(3+)}$ dicitrate transport protein	$Fe^{(3+)}$ dicitrate transport
SGBXF1_04076-79	*fhuBCDA*	$Fe^{(3+)}$-hydroxamate import ABC transporter complex	Iron transport
SGBXF1_02561-63	*fbpABC*	$Fe^{(3+)}$ ABC transporter complex	Iron transport
SGBXF1_04564-66	*feoABC*	Ferrous iron transport proteins	Fe^{2+} ion uptake.
SGBXF1_02150-53	*yfeABCD*	Periplasmic chelated iron-binding proteins	Chelated iron transport, manganese transport
SGBXF1_02959-61	*efeBOU*	Iron uptake system	Fe^{2+} ion uptake
SGBXF1_02201-06	*sufABCDSE*	FeS cluster assembly proteins and cysteine desulfurase	May facilitate iron uptake from extracellular iron chelators under iron limitation

Table S7-Genes involved in nitrogen, sulfur and phosphorous metabolism

Locus tag/operon	Gene/operon name	Product/putative product	Function/Putative function
SGBXF1_02921-24	*narIJHG*	Respiratory nitrate reductase	Nitrate assimilation
SGBXF1_02939-41	*narKXL*	Nitrate/nitrite sensor and transporter	Nitrate assimilation, celular response to nitrate and nitrite
SGBXF1_03564	*narQP*	Nitrate/nitrite sensor	Nitrate assimilation
SGBXF1_03559-61	*napABC*	Periplasmic nitrate reductase	Nitrate assimilation
SGBXF1_04535-37	*nirBDC*	Nitrite reductase	Nitrate assimilation
SGBXF1_01028-29	*amtBR*	Ammonia channel	Ammonia uptake
SGBXF1_04774	*glnA*	Glutamine synthetase	Ammonia assimilation cycle
SGBXF1_04289-90	*gltBD*	Glutamate synthase [NADPH]	Ammonia assimilation cycle
SGBXF1_04775-76	*glnLG*	Nitrogen regulation sensor	Nitrogen regulation
SGBXF1_01371-72	*atzF, Dur1,2*	Allophanate hydrolase<br>Urea amidolyase	Hydrolysis of urea to ammonia and CO2
SGBXF1_00746-49	*cysGDN*	Sulfate adenylyltransferase	Sulfate reduction
SGBXF1_00742-44	*cysJIH*	Sulfite reductase	Sulfate reduction
SGBXF1_03530-33	*cysAWTP*	Sulfate/thiosulfate import ATP-binding proteins	Sulfate transport
SGBXF1_00984-88	*ttrRSBCA*	Tetrathionate reductase and other components	Tetrathionate reduction
SGBXF1_00553-54	*atsAB*	Arylsulfatase	Arylsulfate ester degradation
SGBXF1_01700-04	*ssuBCDAE*	Alkanesulfonate monooxygenase and other components	Alkanesulfonate degradation
SGBXF1_01363-65	*ssuD*	Alkanesulfonate monooxygenase	Alkanesulfonate degradation
SGBXF1_04509-12	*tauDCBA*	Taurine dioxygenase and other components	Taurine and alkanesulfonate degradation
SGBXF1_01033	*bds1*	Aryl/alkyl sulfatase	Enables the use of SDS and 4-nitrocatechol as sulfur source
SGBXF1_02968	*dcys*	D-cysteine desulfhydrase	Use of D-cysteine as sulfur source
SGBXF1_00028<br>SGBXF1_00969	*pstS*	Phosphate transport system substrate-binding protein	Phosphate transport
SGBXF1_00029-31<br>SGBXF1_03605-07	*pstCAB*		
SGBXF1_01358-61	*phnCDE*	phosphonate transport system substrate-binding protein	Phosphonate transport system
SGBXF1_00457-67	*phnFGHIJKLMNOP*	Phosphonates utilization proteins and other elements	Phosphonate degradation

Table S8- Genes involved in the degradation of aromatic compounds and other xenobiotics

Locus tag/operon	Gene/operon	Product/putative product	Function/Putative function
SGBXF1_03049-52	*cdbABCD*	2-halobenzoate 1,2-dioxygenase	Benzoate degradation
SGBXF1_03046-48	*catBCA*	Catechol 1,2-dioxygenase	Beta-ketoadipate pathway
SGBXF1_03042-45	*pcaIJFD*	3-oxoadipate CoA-transferase	
SGBXF1_02519-20	*pcaGH*	Protocatechuate 3,4-dioxygenase	
SGBXF1_02522-23	*pcaCB*	4-carboxymuconolactone decarboxylase, 3-carboxy-cis,cis-muconate cycloisomerase	
SGBXF1_02524	*praI*	Hydroxybenzoate (4-HBA)-3-monoxygenase	Degradation of 4-hydroxybenzoate (4HBA) via protocatechuate
SGBXF1_03089-103	*paaABCDEFGHIJK*	1,2-phenylacetyl-CoA epoxidase and other elements	Phenylacetate degradation
SGBXF1_00562-68	*hpcECBDGH*	3,4-dihydroxyphenylacetate 2,3-dioxygenase and other elements	4-hydroxyphenylacetate degradation
SGBXF1_00571-72	*hpaBC*	4-hydroxyphenylacetate 3-monooxygenase oxygenase	
SGBXF1_02237-38<br>SGBXF1_04338-39	*aaeAB*	p-hydroxybenzoic acid efflux pump	Transport and elimination of aromatic compounds

Table S9- Genes involved in antibiotic resistance and multidrug efflux systems

Locus tag/operon	Gene/operon	Product/putative product	Function/Putative function
SGBXF1_02302	*ampC*	Beta-lactamase	Cephalosporin resistance
SGBXF1_02257	*strA*	Streptomycin 3''-kinase	Streptomycin resistance
SGBXF1_01065	*fsr*	Fosmidomycin resistance protein	Fosmidomycin resistance
SGBXF1_03291	*bcr*	Bicyclomycin resistance protein	Bicyclomycin resistance
SGBXF1_02877	*fosA*	Fosfomycin resistance protein	Fosfomycin resistance
SGBXF1_04240	*uppP*	Undecaprenyl pyrophosphate phosphatase	Bacitracin resistance
SGBXF1_02175-81	*arnBCADTEF*	UDP-4-deoxy-4-formamido-beta-L-arabinose biosynthesis proteins	Resistance to polymyxin and cationic antimicrobial peptides
SGBXF1_00419-20	*basSR*	Two-component regulatory system BasS/BasR	Resistance to polymyxin and cationic antimicrobial peptides
SGBXF1_02230	*pmrC*	Phosphoethanolamine transferase	Resistance to polymyxin
SGBXF1_01645-46<br>SGBXF1_01875-76<br>SGBXF1_03527-28	*macAB*	Macrolide export proteins	Drug export
SGBXF1_01045-46	*acrAB*	Multidrug efflux pump proteins	Drug transmembrane transport
SGBXF1_03567	*acrB*	Multidrug efflux pump subunit	
SGBXF1_02499-501	*oprM-mexAB*	Multidrug resistance proteins	
SGBXF1_01945-47	*oqxABR*	Multidrug efflux proteins	
SGBXF1_03836-37	*emrAB*	Multidrug export proteins	
SGBXF1_03620-25	*mdtABCD*	Multidrug export proteins	Confers resistance against novobiocin and deoxycholate
SGBXF1_01026-27	*mdlAB*	Multidrug resistance-like ATP-binding proteins	Xenobiotic transport and efflux
SGBXF1_02376	*mdtG*	Multidrug resistance protein	Confers resistance against fosfomycin and deoxycholate
SGBXF1_02214	*mdtK*	Multidrug resistance protein	Drug transmembrane transport, antibiotic resistance
SGBXF1_03990	*mdtL*	Multidrug resistance protein	Confers resistance to chloramphenicol
SGBXF1_02845	*mdtH*	Multidrug resistance protein	Confers resistance to norfloxacin and enoxacin
SGBXF1_00095	*mdfA*	Multidrug transporter	Drug transmembrane transport, antibiotic resistance

Table S10- Genes involved in secondary metabolites and antagonistic activities

Locus tag/operon	Gene/operon	Product/putative product	Function/Putative function
SGBXF1_00226-36	-	Bacteriocin/lantibiotic production genes	Bacteriocin production
SGBXF1_00232	-	Bacteriocin/Lantibiotic dehydratase	
SGBXF1_00897-901	*nrps*	Unknown	Siderophore production?
SGBXF1_04372	*srwW*	Serrawetin W1 synthase	Serrawetin W1 production
SGBXF1_02064	*t1pks*	Type I polyketide synthase	Unknown
SGBXF1_00148<br>SGBXF1_02770	*chiA*	Chitinase A	Hydrolysis of N-acetyl-beta-D-glucosaminide (1->4)-beta-linkages in chitin and chitodextrins
SGBXF1_03552	*chiB*	Chitinase B	
SGBXF1_01157	*chiD*	Chitinase D	
SGBXF1_01164	*chiP*	Chitoporin	Involved in the uptake of chitosugars
SGBXF1_01166	*chB*	Chitobiase	Digests the beta-1,4-glycosidic bonds in N-acetylglucosamine (GlcNAc) oligomers
SGBXF1_03554	*gbpA*	Chitin and GlNac-binding protein	Promotes bacterial attachment to GlNac residues and chitin
SGBXF1_00333-36	*prnABCD*	Tryptophan halogenase and other elements	Pyrrolnitrin production
SGBXF1_01846-48	*hcnABC*	Hydrogen cyanide synthase and others	Degradation of amino acids and production of HCN

Table S11- Genes involved in motility, chemotaxis, attachment and quorum-sensing

Locus tag/operon	Gene/operon	Product/putative product	Function/Putative function
SGBXF1_02993-03006	*flgLKJIHGFEDCBAMN*	Flagellar basal-body rod proteins	Flagella biosynthesis
SGBXF1_03009-11	*flhEAB*	Flagellar biosynthesis proteins	
SGBXF1_02969-74	*fliZACDST*	Flagellin and other flagellar proteins	Flagellum-dependent cell motility
SGBXF1_02979-92	*fliEFGHIJKLMONPQR*	Flagellar proteins	
SGBXF1_03022-23	*flhCD*	Flagellar transcriptional regulators	Flagella biosynthesis and flagellum-dependent cell motility
SGBXF1_03020-21	*motBA*	Motility proteins	Motility
SGBXF1_04010-11	*rssBA*	Regulation of swarming motility proteins	Regulation of swarming motility
SGBXF1_04372	*srwW*	Serrawetin W1 synthase	Serrawetin W1 production, motility
SGBXF1_03012-15	*cheZYBR*	Chemotaxis proteins	Chemotaxis
SGBXF1_03018-19	*cheWA*	Chemotaxis proteins	
SGBXF1_03016	*tap*	Methyl-accepting chemotaxis protein	
SGBXF1_03017	*tsr*	Methyl-accepting chemotaxis protein	
SGBXF1_00405	*tar*	Methyl-accepting chemotaxis protein	
SGBXF1_00451 SGBXF1_00083	*tsr*	Methyl-accepting chemotaxis protein	
SGBXF1_1467-72	*ecpRABCDE*	Fimbria proteins and other elements	Fimbria biogenesis
SGBXF1_01487-90	*fimA, others*	Fimbria A protein and other elements	
SGBXF1_03119-26	*smfA, others*	Fimbria A protein and other elements	
SGBXF1_04607-15	*smfA, others*	Fimbria A protein and other elements	
SGBXF1_00172-84	*wec operon*	Lipopolysaccharide biosynthesis proteins	Lipopolysaccharide (LPS) biosynthesis
SGBXF1_01562-81	*OA cluster*	dTDP-4-dehydrorhamnose reductase and other elements	O-antigen biosynthesis, LPS biosynthesis
SGBXF1_00510-11	*lptFG*	Lipopolysaccharide export system permease protein	LPS export system
SGBXF1_04308-9	*kdsCD*	3-deoxy-D-manno-octulosonate 8-phosphate phosphatase, Arabinose 5-phosphate isomerase	LPS biosynthesis
SGBXF1_04310-12	*lptCAB*	Lipopolysaccharide export system ATP-binding protein	LPS export system
SGBXF1_01547-56	*wza wzb wzc*	Tyrosine-protein kinase and glycosyl transferases	Exopolysaccharide biosynthesis
SGBXF1_02925-37	*eps, wzc*	Tyrosine-protein kinase and glycosyl transferases	Exopolysaccharide biosynthesis
SGBXF1_03349	*ndvB*	Protein NdvB	Involved in the production of beta-(1,2)-glucan
SGBXF1_00159- 64	*bcsGFEQABZC*	Cellulose synthase and other elements	Cellulose biosynthesis
SGBXF1_04088-89	*qseCB*	Sensor proteins	Quorum-sensing

SGBXF1_00787	*luxS*	S-ribosylhomocysteine lyase	Synthesis of autoinducer 2 (AI-2), quorum-sensing
SGBXF1_00210	*rhtB*	Homoserine/homoserine lactone efflux protein	Quorum-sensing
SGBXF1_00292<br>SGBXF1_04493	*tufA*	Elongation factor Tu	MAMP
SGBXF1_00688<br>SGBXF1_02390	*ftsI*	Peptidoglycan synthase	Peptidoglycan biosynthesis

Table S12- Genes involved in major carbohydrate degradation pathways

Locus tag/operon	Gene/operon	Product/putative product	Function/Putative function
SGBXF1_03118	*gcd*	Glucose dehydrogenase	Glucose degradation
SGBXF1_03269-71	*fruAKB*	1-phosphofructokinase and others	Fructose degradation
SGBXF1_00100-101	*xylAB*	Xylulose kinase, Xylose isomerase	Xylose degradation
SGBXF1_02276-77	*araAB*	L-arabinose isomerase, Ribulokinase	Arabinose degradation
SGBXF1_00003-08	*rbsRKBCAD*	Ribokinase and others	Ribose degradation
SGBXF1_04571-72	*malQP*	4-alpha-glucanotransferase, Maltodextrin phosphorylase	Maltose degradation
SGBXF1_02308	*manA*	Mannose-6-phosphate isomerase	Mannose degradation
SGBXF1_00474-5	*treAB*	Trehalose-6-phosphate hydrolase and transporter	Trehalose degradation
SGBXF1_01229-31	*galMKT*	Galactokinase and others	Galactose degradation
SGBXF1_02100	*sacA*	Sucrose-6-phosphate hydrolase	Sucrose degradation
SGBXF1_04175	*scrB*		
SGBXF1_03205	*lacZ*	Beta-galactosidase	Lactose degradation
SGBXF1_01999-2000	*ebgAC*		
SGBXF1_00960	*gatY*	D-tagatose-1,6-bisphosphate aldolase	Galactitol catabolism
SGBXF1_03961	*gatZ*		
SGBXF1_00075-77	*mtlADR*	Mannitol-1-phosphate 5-dehydrogenase and others	Mannitol degradation
SGBXF1_03635-38	*slrAEBD*	Sorbitol-6-phosphate 2-dehydrogenase and others	Sorbitol degradation
SGBXF1_01158-61	*nagBACD*	N-acetylglucosamine-6-phosphate deacetylase and others	N-acetylglucosamine degradation
SGBXF1_01166	*chb*	Chitobiase	Digests the beta-1,4-glycosidic bonds in N-acetylglucosamine (GlcNAc)
SGBXF1_03957-62	*PTS, kbaZ*	N-acetylgalactosamine permeasse, D-tagatose-1,6-bisphosphate aldolase	D-galactosamine degradation
SGBXF1_00773	*chbG*	Chitooligosaccharide deacetylase	Involved in the degradation of acetylated chitooligosaccharides chitobiose and chitotriose
SGBXF1_02604-07	*nagK*	N-acetyl-D-glucosamine kinase and others	N-acetyl-D-galactosamine degradation
SGBXF1_00061	*malS*	Amylase	Degradation of amylose, starch, amylopectin, and maltodextrins
SGBXF1_01337	*amyA*	Amylase	
SGBXF1_02540	*palH*	Alpha-glucosidase	Degrades maltose, palatinose, maltulose, trehalose, trehalulose, turanose, leucrose, sucrose and maltitol
SGBXF1_03131-32	*melBA*	Alpha-glucosidase	Melibiose degradation
SGBXF1_02538	*malL*	Maltase, Isomaltase	Maltose degradation
SGBXF1_04417	*malL*	Oligo-1,6-glucosidase, Maltase	
SGBXF1_00974	*malZ*	Maltodextrin glucosidase	Degrades maltotriose and longer maltodextrins with a chain length of up to 7 glucose units
SGBXF1_01996	*ygjK*	Glucosidase	Nigerose degradation
SGBXF1_02399	*bglB*	Cellobiase	Cellobiose, Gentibiose degradation
SGBXF1_01321	*bglX*	Beta-glucosidase	Hydrolysis of terminal, non-reducing beta-D-glucosyl

			residues with release of beta-D-glucose
SGBXF1_00138	*blgB*	6-phospho-beta-glucosidase	Hydrolysis of phosphorylated beta-glucosides into glucose-6-phosphate (G-6-P) and aglycone
SGBXF1_00771	*gmuD*	6-phospho-beta-glucosidase	Glucomannan degradation
SGBXF1_01870<br>SGBXF1_04531	*chbF*	6-phospho-beta-glucosidase	Hydrolyzes a wide variety of P-beta-glucosides including cellobiose-6P, salicin-6P, arbutin-6P and others
SGBXF1_02517	*bglA*	6-phospho-beta-glucosidase	Hydrolysis of phosphorylated beta-glucosides into glucose-6-phosphate (G-6-P) and aglycone
SGBXF1_02359<br>SGBXF1_04187	*bglC*	Aryl-phospho-beta-D-glucosidase	Hydrolysis of aryl-phospho-beta-D-glucosides

Table S13- Genes involved in sugars transport

Locus tag/operon	Gene/operon	Product/putative product	Function/Putative function
SGBXF1_04418-20	*malEFG*	Maltose/maltodextrin transport system	Maltose/maltodextrin transport system
SGBXF1_02539<br>SGBXF1_04416	*malK*	Multiple sugar transport system ATP-binding protein	Trehalose<br>Maltose/maltodextrin transport system
SGBXF1_02329<br>SGBXF1_02900	*msmX*	Multiple sugar transport system ATP-binding protein	Maltose/maltodextrin transport system, alpha-Glucoside transport system
SGBXF1_02541	*thuGFE*	Trehalose/maltose transport system proteins	Trehalose/maltose transport system
SGBXF1_02278-80	*araFGH*	L-arabinose transport system proteins	L-Arabinose transport system
SGBXF1_01529-31	*mglBAC*	Methyl-galactoside transport system substrate-binding protein	Methyl-galactoside transport system
SGBXF1_00005<br>SGBXF1_00958<br>SGBXF1_04031	*rbsB*	Ribose transport system components	Ribose transport system
SGBXF1_00006<br>SGBXF1_00957<br>SGBXF1_04030	*rbsC*		
SGBXF1_00007<br>SGBXF1_00956<br>SGBXF1_04029	*rbsA*		
SGBXF1_01928	*ptsG*	PTS system, glucose-specific components	PTS system, glucose
SGBXF1_03524	*crr*		
SGBXF1_01162	*nagE*	PTS system, N-acetylglucosamine-specific IIA component	PTS system, N-acetylglucosamine
SGBXF1_02299	*malX*	PTS system, maltose/glucose-specific IIB component	PTS system, maltose and glucose
SGBXF1_00475	*treB*	PTS system, trehalose-specific IIB component	PTS system, trehalose
SGBXF1_03269	*fruA*	PTS system, fructose-specific components	PTS system, fructose
SGBXF1_03271	*fruB*		
SGBXF1_00075	*mtlA*	PTS system, mannitol-specific IIA component	PTS system, mannitol
SGBXF1_01869<br>SGBXF1_04188<br>SGBXF1_04530	*chbA*	PTS system N,N'-diacetylchitobiose-specific components	PTS system N,N'-diacetylchitobiose
SGBXF1_00769	*chbB*		
SGBXF1_00770	*chbC*		
SGBXF1_01868<br>SGBXF1_04190	*celA or licB*	PTS system, cellobiose-specific IIB component,<br>Lichenan permease	PTS system, cellobiose, lichenan
SGBXF1_01871<br>SGBXF1_04189	*celB or licC*	PTS system, cellobiose-specific IIC component,<br>Lichenan permease IIC component	
SGBXF1_03637	*srlB*	PTS system, glucitol/sorbitol-specific IIA component	PTS system, glucitol/sorbitol
SGBXF1_02862<br>SGBXF1_04227<br>SGBXF1_04226	*manX*	PTS system, mannose-specific components	PTS system, mannose
SGBXF1_02863<br>SGBXF1_04225	*manY*		
SGBXF1_02864	*manZ*		
SGBXF1_03957	*yadI*	PTS system	PTS system
SGBXF1_03960	*agaV*		

SGBXF1_03959	*agaC*	PTS system, N-acetylgalactosamine-specific components	PTS system, N-acetylgalactosamine
SGBXF1_03958	*agaD*		
SGBXF1_02289	*ulaC*	PTS system, ascorbate-specific component	PTS system, ascorbate
SGBXF1_02293	*ulaB*		
SGBXF1_02292	*ulaA*		
SGBXF1_02333<br>SGBXF1_02599<br>SGBXF1_02896	*ABC.MS.S*	Multiple sugar transport system substrate-binding protein	Putative multiple sugar transport system
SGBXF1_02332<br>SGBXF1_02600<br>SGBXF1_02897	*ABC.MS.P*	Multiple sugar transport system permease protein	
SGBXF1_02331<br>SGBXF1_02601<br>SGBXF1_02898	*ABC.MS.P1*	Multiple sugar transport system permease protein	
SGBXF1_01538<br>SGBXF1_04629	*ABC.SS.S*	Simple sugar transport system substrate-binding protein	Putative simple sugar transport system
SGBXF1_03259<br>SGBXF1_04631<br>SGBXF1_04632	*ABC.SS.P*	Simple sugar transport system permease protein	
SGBXF1_03258<br>SGBXF1_04630	*ABC.SS.A*	Simple sugar transport system ATP-binding protein	

Table S14- Genes involved in rhizopine transport and rhizopine and myo-inositol degradation

Locus tag/operon	Gene/operon	Product/putative product	Function/Putative function
SGBXF1_01983<br>SGBXF1_01986<br>SGBXF1_02411	*mocB*	Rhizopine-binding protein	Involved in rhizopine (L-3-O-methyl-scyllo-inosamine) catabolism
SGBXF1_02068	*iolE*,<br>*mocC*	Inosose dehydratase<br>Rhizopine catabolism protein	Dehydration of inosose, Involved in rhizopine catabolism
SGBXF1_02409	*iolX*	Scyllo-inositol 2-dehydrogenase	Oxidation of scyllo-inositol to 2,4,6/3,5-pentahydroxycyclohexanone (scyllo-inosose)
SGBXF1_04592	*iolE*	Inosose dehydratase	Myo-inositol degradation
SGBXF1_04593	*iolH*	Protein IOHL	
SGBXF1_04594	*iolG*	Inositol 2-dehydrogenase	
SGBXF1_04595	*iolD*	3D-(3,5/4)-trihydroxycyclohexane-1,2-dione hydrolase	
SGBXF1_04596	*iolC*	5-dehydro-2-deoxygluconokinase	
SGBXF1_04598	*iolB*	5-deoxy-glucuronate isomerase	
SGBXF1_04599	*iolA*	Methylmalonate semialdehyde dehydrogenase	

Table S15- Genes involved in organic and sugar acids degradation

Locus tag/operon	Gene/operon	Product/putative product	Function/Putative function
SGBXF1_01196-99	*sdhCBAD*	Succinate dehydrogenase and others	Succinate degradation
SGBXF1_00411	*mdh*	Malate dehydrogenase	Malate degradation
SGBXF1_04442-44	*aceKAB*	Isocitrate lyase	Isocitrate degradation
SGBXF1_00328	*acs*	Acetyl-coenzyme A synthetase	Acetate degradation
SGBXF1_04438<br>SGBXF1_04586	*gntK*	D-gluconate kinase	Gluconate degradation
SGBXF1_02156-8	*gadH*	Gluconate dehydrogenase	
SGBXF1_00355-58	*frdABCD*	Fumarate reductase and others	Fumarate degradation<br>Oxaloacetate degradation
SGBXF1_02357	*odc*	Oxalate decarboxylase	Oxalate degradation
SGBXF1_02704	*acnA*	Aconitate hydratase	Citrate degradation
SGBXF1_04105	*acnB*	Aconitate hydratase	
SGBXF1_03197-3203	*citTGXFEDC*	Citrate lyase and transporters	
SGBXF1_02050	*icd*	Isocitrate dehydrogenase [NADP]	Oxaloacetate degradation
SGBXF1_04118	*fumA*	Fumarate hydratase class I, aerobic	Malate degradation and interconversion
SGBXF1_02310	*fumC*	Fumarate hydratase class II	
SGBXF1_02447-58	*fdhF*, *hyc* operon	Formate hydrogenlyase complex	Formate degradation
SGBXF1_04265	*uxaA*	Altronate dehydratase	D-glucuronate, D-altronate, D-fructuronate degradation
SGBXF1_04266	*uxaC*	Uronate isomerase	
SGBXF1_03279	*uxuA*	Mannonate dehydratase	
SGBXF1_03280<br>SGBXF1_03632	*uxuB*	D-mannonate oxidoreductase	
SGBXF1_03629	*rspA*	D-galactonate dehydratase family member RspA	
SGBXF1_00064<br>SGBXF1_04661	*kdgK*	2-dehydro-3-deoxygluconokinase	
SGBXF1_02815<br>SGBXF1_04441	*eda*	KHG/KDPG aldolase	

Table S16- Genes involved in amino acid metabolism

Locus tag/operon	Gene/operon	Product/putative product	Function/Putative function
SGBXF1_04023	*serA*	D-3-phosphoglycerate dehydrogenase	Serine biosynthesis, glycerate-3P
SGBXF1_01671	*serC*	Phosphoserine aminotransferase	
SGBXF1_00604	*serB*	Phosphoserine phosphatase	
SGBXF1_04425	*lysC*	Aspartate kinase	Threonine biosynthesis, aspartate => homoserine => threonine
SGBXF1_00622	*thrA*	Bifunctional aspartokinase / homoserine dehydrogenase	
SGBXF1_04692	*metL*	Bifunctional aspartokinase / homoserine dehydrogenase 2	
SGBXF1_04584	*asd*	Aspartate-semialdehyde dehydrogenase	
SGBXF1_00623	*thrB1*	Homoserine kinase	
SGBXF1_00624	*thrC*	Threonine synthase	
SGBXF1_00504<br>SGBXF1_01482<br>SGBXF1_04153	*betA*	Choline dehydrogenase	Betaine biosynthesis
SGBXF1_01481<br>SGBXF1_04055	*betB*	Betaine-aldehyde dehydrogenase	
SGBXF1_04722	*cysE*	Serine O-acetyltransferase	Cysteine biosynthesis, serine => cysteine
SGBXF1_01024<br>SGBXF1_03521	*cysK*	Cysteine synthase A	
SGBXF1_03529	*cysM*	Cysteine synthase B	
SGBXF1_02968	*dcys*	D-cysteine desulfhydrase	D-cysteine degradation
SGBXF1_01602	*cbs*	Cystathionine beta-synthase	Cysteine biosynthesis, homocysteine + serine => cysteine
SGBXF1_01601	*CTH*	Cystathionine gamma-lyase [	
SGBXF1_04425	*lysC*	Aspartate kinase	Methionine biosynthesis, apartate => homoserine => methionine
SGBXF1_00622	*thrA*	Bifunctional aspartokinase / homoserine dehydrogenase	
SGBXF1_04692	*metL*	Bifunctional aspartokinase / homoserine dehydrogenase 2	
SGBXF1_04584	*asd*	Aspartate-semialdehyde dehydrogenase	
SGBXF1_04445	*metA*	Homoserine O-succinyltransferase	
SGBXF1_04693	*metB*	Cystathionine gamma-synthase	
SGBXF1_04180	*metC*	Cystathionine beta-lyase	
SGBXF1_04436	*metH*	5-methyltetrahydrofolate--homocysteine methyltransferase	
SGBXF1_00258<br>SGBXF1_03163	*metE*	5-methyltetrahydropteroyltriglutamate--homocysteine methyltransferase	
SGBXF1_04075	*metK*	S-adenosylmethionine synthetase	Methionine salvage pathway
SGBXF1_04101	*speD*	S-adenosylmethionine decarboxylase	
SGBXF1_04100	*speE*	Spermidine synthase	
SGBXF1_00721<br>SGBXF1_01611	*mtnN*	Adenosylhomocysteine nucleosidase	
SGBXF1_00869	*mtnK*	5-methylthioribose kinase	
SGBXF1_00868	*mtnA*	Methylthioribose-1-phosphate isomerase	
SGBXF1_00865	*mtnB*	Methylthioribulose-1-phosphate dehydratase	
SGBXF1_00866	*mtnC*	Enolase-phosphatase E1	
SGBXF1_00867	*mtnD*	1,2-dihydroxy-3-keto-5-methylthiopentene dioxygenase	
SGBXF1_03106<br>SGBXF1_04392	*tyrB*	Aromatic-amino-acid transaminase	

SGBXF1_00667 SGBXF1_00681 SGBXF1_02340 SGBXF1_03358 SGBXF1_03508 SGBXF1_04054 SGBXF1_04669	*ilvL*	Acetolactate synthase I/II/III large subunit	Valine/isoleucine biosynthesis, pyruvate => valine / 2-oxobutanoate => isoleucine
SGBXF1_00666 SGBXF1_00682	*ilvH*	Acetolactate synthase I/III small subunit	
SGBXF1_04668	*ilvM*	Acetolactate synthase II small subunit	
SGBXF1_04662	*ilvC*	Ketol-acid reductoisomerase	
SGBXF1_04666	*ilvD*	Dihydroxy-acid dehydratase	
SGBXF1_04667	*ilvE*	Branched-chain amino acid aminotransferase	
SGBXF1_00678 SGBXF1_01889	*leuA*	2-isopropylmalate synthase	Leucine biosynthesis, 2-oxoisovalerate => 2-oxoisocaproate
SGBXF1_00676	*leuC*	3-isopropylmalate/(R)-2-methylmalate dehydratase large subunit	
SGBXF1_00675	*leuD*	3-isopropylmalate/(R)-2-methylmalate dehydratase small subunit	
SGBXF1_00677	*leuB*	3-isopropylmalate dehydrogenase	
SGBXF1_04425	*lysC*	Aspartate kinase	Lysine biosynthesis, succinyl-DAP pathway, aspartate => lysine
SGBXF1_00622	*thrA*	Bifunctional aspartokinase / homoserine dehydrogenase 1	
SGBXF1_04692	*metL*	Bifunctional aspartokinase / homoserine dehydrogenase 2	
SGBXF1_04584	*asd*	Aspartate-semialdehyde dehydrogenase	
SGBXF1_02247 SGBXF1_03583	*dapA*	4-hydroxy-tetrahydrodipicolinate synthase	
SGBXF1_00645	*dapB*	4-hydroxy-tetrahydrodipicolinate reductase	
SGBXF1_03903	*dapD*	2,3,4,5-tetrahydropyridine-2-carboxylate N-succinyltransferase	
SGBXF1_04524	*argD*	Acetylornithine/N-succinyldiaminopimelate aminotransferase	
SGBXF1_03575	*dapE*	Succinyl-diaminopimelate desuccinylase	
SGBXF1_00197	*dapF*	Diaminopimelate epimerase	
SGBXF1_03949	*lysA*	Diaminopimelate decarboxylase	
SGBXF1_00888	*proB*	Glutamate 5-kinase	Proline biosynthesis, glutamate => proline
SGBXF1_00889	*proA*	Glutamate-5-semialdehyde dehydrogenase	
SGBXF1_04127	*proC*	Pyrroline-5-carboxylate reductas	
SGBXF1_03925	*argAB*	Amino-acid N-acetyltransferase	Ornithine biosynthesis, glutamate => ornithine
SGBXF1_04687	*argB*	Acetylglutamate kinase	
SGBXF1_04688	*argC*	N-acetyl-gamma-glutamyl-phosphate reductase	
SGBXF1_04524	*argD*	Acetylornithine/N-succinyldiaminopimelate aminotransferase	
SGBXF1_04689	*argE*	Acetylornithine deacetylase	
SGBXF1_01589	*hisG*	ATP phosphoribosyltransferase	Histidine biosynthesis, PRPP => histidine
SGBXF1_01582	*hisIE*	Phosphoribosyl-ATP pyrophosphohydrolase / phosphoribosyl-AMP cyclohydrolase	
SGBXF1_01584	*hisA*	Phosphoribosylformimino-5-aminoimidazole carboxamide ribotide isomerase	

SGBXF1_01585	*hisH*	Glutamine amidotransferase	
SGBXF1_01583	*hisF*	Cyclase	
SGBXF1_01587	*hisC*	Histidinol-phosphate aminotransferase	
SGBXF1_01586	*hisB*	Imidazoleglycerol-phosphate dehydratase / histidinol-phosphatase	
SGBXF1_01588	*hisD*	Histidinol dehydrogenase	
SGBXF1_00732<br>SGBXF1_00737<br>SGBXF1_02105	*hutH*	Histidine ammonia-lyase	Histidine degradation, histidine => N-formiminoglutamate => glutamate
SGBXF1_00738<br>SGBXF1_02107	*hutU*	Urocanate hydratase	
SGBXF1_02095	*hutI*	Imidazolonepropionase	
SGBXF1_02094	*hutG*	Formiminoglutamase	
SGBXF1_00798<br>SGBXF1_01226<br>SGBXF1_02194	*aroG*	3-deoxy-7-phosphoheptulonate synthase	Shikimate pathway, phosphoenolpyruvate + erythrose-4P => chorismate
SGBXF1_04544	*aroB*	3-dehydroquinate synthase	
SGBXF1_04362	*aroQ*	3-dehydroquinate dehydratase II	
SGBXF1_04177<br>SGBXF1_04450	*aroE*	Shikimate dehydrogenase	
SGBXF1_00945<br>SGBXF1_04545	*aroK*	Shikimate kinase	
SGBXF1_01672	*aroA*	3-phosphoshikimate 1-carboxyvinyltransferase	
SGBXF1_03437	*aroC*	Chorismate synthase	
SGBXF1_02716	*trpE*	Anthranilate synthase component I	Tryptophan biosynthesis, chorismate => tryptophan
SGBXF1_02717	*trpG*	Anthranilate synthase component II	
SGBXF1_02718	*trpD*	Anthranilate phosphoribosyltransferase	
SGBXF1_02719	*trpCF*	Indole-3-glycerol phosphate synthase / phosphoribosylanthranilate isomerase	
SGBXF1_02721	*trpA*	Tryptophan synthase alpha chain	
SGBXF1_02720	*trpB*	Tryptophan synthase beta chain	
SGBXF1_00799	*tyrA*	Chorismate mutase / prephenate dehydrogenase	Phenylalanine biosynthesis, chorismate => phenylalanine
SGBXF1_02092	*pheA1*	Chorismate mutase	
SGBXF1_04394	*pheC*	Cyclohexadienyl dehydratase	
SGBXF1_00800	*pheA*	Chorismate mutase / prephenate dehydratase	
SGBXF1_03106<br>SGBXF1_04392	*tyrB*	Aromatic-amino-acid transaminase	
SGBXF1_00800	*pheA*	Chorismate mutase / prephenate dehydratase	Tyrosine biosynthesis, chorismate => tyrosine
SGBXF1_02092	*pheA1*	Chorismate mutase	
SGBXF1_00799	*tyrA*	Chorismate mutase / prephenate dehydrogenase	
SGBXF1_03106<br>SGBXF1_04392	*tyrB*	Aromatic-amino-acid transaminase	
SGBXF1_3113	*davD*	Glutarate-semialdehyde dehydrogenase	Lysine catabolism

Table S17- Genes involved in amino acid transport

Locus tag/operon	Gene/operon	Product/putative product	Function/Putative function
SGBXF1_01318	*argT*	Lysine/arginine/ornithine transport system substrate-binding protein	Lysine/arginine/ornithine transport system
SGBXF1_02420<br>SGBXF1_03389	*hisM*	Histidine transport system permease protein	
SGBXF1_03390	*hisQ*	Histidine transport system permease protein	
SGBXF1_03388	*hisP*	Histidine transport system ATP-binding protein	
SGBXF1_02418<br>SGBXF1_03391	*hisJ*	Histidine transport system substrate-binding protein	Histidine transport system
SGBXF1_02420<br>SGBXF1_03389	*hisM*	Histidine transport system permease protein	
SGBXF1_03390	*hisQ*	Histidine transport system permease protein	
SGBXF1_03388	*hisP*	Histidine transport system ATP-binding protein	
SGBXF1_01442-44	*glnOPH*	Glutamine transport system proteins	Glutamine transport system
SGBXF1_01626-30	*artJMQIP*	Arginine transport system proteins	Arginine transport system
SGBXF1_01138-41	*gltLKJI*	Glutamate/aspartate transport system protein	Glutamate/aspartate transport system
SGBXF1_02965-67	*yecCS, fliY*	Cystine transport system proteins	Cystine transport system
SGBXF1_00244<br>SGBXF1_02581<br>SGBXF1_03227	*livK*	Branched-chain amino acid transport system substrate-binding protein	Branched-chain amino acid transport system
SGBXF1_00245<br>SGBXF1_02577<br>SGBXF1_03226	*livH*	Branched-chain amino acid transport system permease protein	
SGBXF1_00246<br>SGBXF1_02578<br>SGBXF1_03225	*livM*	Branched-chain amino acid transport system permease protein	
SGBXF1_00247<br>SGBXF1_02579<br>SGBXF1_03224	*livG*	Branched-chain amino acid transport system ATP-binding protein	
SGBXF1_00248<br>SGBXF1_02580<br>SGBXF1_03223	*livF*	Branched-chain amino acid transport system ATP-binding protein	
SGBXF1_01308<br>SGBXF1_01345<br>SGBXF1_01991<br>SGBXF1_03864	*metQ*	D-methionine transport system substrate-binding protein	D-Methionine transport system
SGBXF1_01346<br>SGBXF1_03863	*metI*	D-methionine transport system permease protein	
SGBXF1_01347<br>SGBXF1_03862	*metN*	D-methionine transport system ATP-binding protein	
SGBXF1_00033<br>SGBXF1_00753<br>SGBXF1_00854<br>SGBXF1_01936<br>SGBXF1_02433	*ABC.PA.S*	Polar amino acid transport system substrate-binding protein	Putative polar amino acid transport system
SGBXF1_00034<br>SGBXF1_00035<br>SGBXF1_00752<br>SGBXF1_00855<br>SGBXF1_00856<br>SGBXF1_01109	*ABC.PA.P*	Polar amino acid transport system permease protein	

SGBXF1_00751<br>SGBXF1_00857<br>SGBXF1_01108	*ABC.PA.A*	Polar amino acid transport system ATP-binding protein	
SGBXF1_02556<br>SGBXF1_02658<br>SGBXF1_02742<br>SGBXF1_02743	*oppA*	Oligopeptide transport system substrate-binding protein	Oligopeptide transport system
SGBXF1_02741	*oppB*	Oligopeptide transport system permease protein	
SGBXF1_02740	*oppC*	Oligopeptide transport system permease protein	
SGBXF1_02739	*oppD*	Oligopeptide transport system ATP-binding protein	
SGBXF1_02738	*oppF*	Oligopeptide transport system ATP-binding protein	
SGBXF1_00149<br>SGBXF1_03507	*dppA*	Dipeptide transport system substrate-binding protein	Dipeptide transport system
SGBXF1_00150	*dppB*	Dipeptide transport system permease protein	
SGBXF1_00151	*dppC*	Dipeptide transport system permease protein	
SGBXF1_00152	*dppD*	Dipeptide transport system ATP-binding protein	
SGBXF1_00153	*dppF*	Dipeptide transport system ATP-binding protein	
SGBXF1_02675	*sapA*	Cationic peptide transport system substrate-binding protein	Cationic peptide transport system
SGBXF1_02676	*sapB*	Cationic peptide transport system permease protein	
SGBXF1_02677	*sapC*	Cationic peptide transport system permease protein	
SGBXF1_02678	*sapD*	Cationic peptide transport system ATP-binding protein	
SGBXF1_02679	*sapF*	Cationic peptide transport system ATP-binding protein	

Table S18- Genes involved in flavonoids, phenylpropanoids and other phenolics metabolism

Locus tag/operon	Gene/operon	Product/putative product	Function/Putative function
SGBXF1_00927<br>SGBXF1_02885<br>SGBXF1_04589	*yhhW*	Quercetin dioxygenase	Is involved quercetin degradation, which is part of Flavonoid metabolism
SGBXF1_02439<br>SGBXF1_03028	*nodD*	Nodulation protein D	Regulator that binds flavonoids as inducers
SGBXF1_00893	*fdc*	Ferulate decarboxylase	Catalyzes the reversible decarboxylation of aromatic carboxylic acids like ferulic acid, p-coumaric acid or cinnamic acid
SGBXF1_00562-572	*hpaBC*	HPA monoxygenase	Hydroxylation of tyrosol and various cinnamic acid derivatives;<br>Phenol oxidation to catechol
SGBXF1_03026	*curA*	NADPH-dependent curcumin reductase	Reduction of curcumin and other phenolics

Table S19- Genes encoding for lipolytic enzymes

Locus tag/operon	Gene/operon	Product/putative product	Function/Putative function
SGBXF1_00070	*lip1*	Lipase*	Triglyceride lipase activity
SGBXF1_00549	*ytpA*	Monoacylglycerol lipase	Hydrolyzes glycerol monoesters of long-chain fatty acids
SGBXF1_02235	*lipA*	Triacylglycerol lipase	Triglyceride lipase activity
SGBXF1_03745-46	*phlAB*	Phospholipase A	Lipid degradation
SGBXF1_04161	*plcN*	Non-hemolytic phospholipase C	Hydrolyzes phosphatidylserine as well as phosphatidylcholine
SGBXF1_02568	*are*	Arylesterase	Degradation of various p-nitrophenyl phosphates, aromatic esters and p-nitrophenyl fatty acids in vitro
SGBXF1_00386	*yjfP*	Esterase	Activity toward palmitoyl-CoA and pNP-butyrate
SGBXF1_01173	*ybfF*	Esterase	Activity toward palmitoyl-CoA, malonyl-CoA and pNP-butyrate
SGBXF1_02199	*menI*	1,4-dihydroxy-2-naphthoyl-CoA hydrolase	Hydrolysis of 1,4-dihydroxy-2-naphthoyl-CoA and other acyl-CoA thioesters
SGBXF1_02306	*estB*	Esterase	Acts on short-chain (C4-C6) fatty acid esters and triglycerides, including tertiary alcohol esters
SGBXF1_04210	*yqiA*	Esterase	Activity toward palmitoyl-CoA, malonyl-CoA and pNP-butyrate.
SGBXF1_00207	*pldA*	Phosphatidylcholine 1-acylhydrolase	Lipid degradation
SGBXF1_00211	*pldB*	Lysophospholipase L2, Lecithinase B	Lipid degradation

*42% identity to *Xenorhabdus luminescens* lipase (Wang and Dowds, 1993).

Reference

Wang H, Dowds BCA. Phase variation in *Xenorhabdus luminescens*: Cloning and sequencing of the lipase gene and analysis of its expression in primary and secondary phases of the bacterium. *J Bacteriol* 1993; 175:1665–73.

Table S20- Genes encoding for extracellular proteases

Locus tag/operon	Gene/operon	Product/putative product	Function/Putative function
SGBXF1_00223	*przN*	Serralysin*	Several proteolytic activities
SGBXF1_02114	*prtA*	Serralysin§	Inhibition of antibacterial peptides
SGBXF1_02115	*prtA*	Serralysin#	Inhibition of antibacterial peptides
SGBXF1_02407	*przN*	Serralysin**	Several proteolytic activities
SGBXF1_03669	*prtS*	Grimelysin	Actin degradation and possibly several proteolytic activities
SGBXF1_04648	*prtS*	Extracellular serine protease	Several proteolytic activities
SGBXF1_04649	*prtS*	Extracellular serine protease	Several proteolytic activities

*aprox. 92% identity
**aprox. 60% identity
to *S. marcescens* HR-3 serralysin with insecticidal activity (high doses) (Tao et al., 2006)

*aprox. 92% identity
**aprox. 59% identiity
to *S. marcescens* ATCC 21074 serralysin, UniProtKB - P07268 (PRZN_SERME), which allows an emerging moth to dissolve its cocoon.

*aprox. 62% identity
**aprox. 54% identity
to *Erwinia chrysanthemi* EC16 serralysin homolog which presents gelatinase activity, but is not involved in the virulence of this strain. Interestingly, high levels of serralysin decreased pectate lyase activity (Dhaler et al., 1990).

§aprox. 55% identity
#aprox. 68% identity
to *Photorhabdus* sp. Az29 proteases involved in the inhibition of insect antibacterial peptides. Reduces the antibacterial activity of *G. mellonella* hemolymph by 50%. Reduces the antibacterial activity of cecropin A by 80% and cecropin B by 75% (Cabral et al., 2004).

References

Tao K, Long Z, Liu K, Tao Y, Liu S. Purification and properties of a novel insecticidal protein from the locust pathogen *Serratia marcescens* HR-3. *Curr Microbiol* 2006; 52, 45–49.

Dahler GS, Barras F, Keen NT. Cloning of genes encoding extracellular metalloproteases from *Erwinia chrysanthemi* Ec16. *Phytopathology* 1990; 80:983–4.

Cabral CM, Cherqui A, Pereira A, Simões N. Purification and characterization of two distinct metalloproteases secreted by the entomopathogenic bacterium *Photorhabdus* sp. strain Az29.

Table S21- Genes involved in ROS stress response and protection

Locus tag/operon	Gene/operon	Product/putative product	Function/Putative function
SGBXF1_00084	*sodA*	Superoxide dismutase [Mn]	Destroys superoxide anion radicals
SGBXF1_02210	*sodB*	Superoxide dismutase [Fe]	
SGBXF1_02236	*sodC*	Superoxide dismutase [Cu-Zn]	
SGBXF1_03342	*katA*	Catalase	Protect cells from the toxic effects of hydrogen peroxide
SGBXF1_03212	*katG*	Catalase-peroxidase	
SGBXF1_03828	*ahpD*	Alkyl peroxidase	Peroxidase active against hydrogen and alkyl peroxides serves as peroxynitrite reductase
SGBXF1_02663	*tpx*	Thyol peroxidase	Removes peroxides or H2O2
SGBXF1_00141-42	*ohrBR*	Organic hydroperoxide resistance protein OhrB	Involved in organic hydroperoxide resistance
SGBXF1_04682	*hyPrx5*	Hybrid peroxiredoxin	Peroxidase and peroxiredoxin activity
SGBXF1_02246<br>SGBXF1_03110	*gstA*	Glutathione S-transferase	Defense against oxidative stress
SGBXF1_01597<br>SGBXF1_01654	*gstB*	Glutathione S-transferase	
SGBXF1_04252	*yfcF*	Glutathione S-transferase	
SGBXF1_01515-18	*gsiABCD*	Glutathione import system proteins	Glutathione import system
SGBXF1_02193	*gpx*	Glutathione peroxidase	Detoxification of Reactive Oxygen Species
SGBXF1_04122	*gshB*	Glutathione synthetase	Glutathione biosynthesis
SGBXF1_04644	*gor*	Glutathione reductase	Glutathione metabolism
SGBXF1_01616<br>SGBXF1_02224<br>SGBXF1_02847<br>SGBXF1_04725	*grxA*	Glutaredoxin	Cell redox homeostasis
SGBXF1_03829	*nrdH*	Glutaredoxin-like protein	
SGBXF1_00812-28	*arpe*	Arylpolyene	Involved in oxidative stress resistance
SGBXF1_03704	*hmp*	Nitric oxide dioxygenase	Nitrosative stress response
SGBXF1_02201-06	*sufABCDSE*	FeS cluster assembly proteins and cysteine desulfurase	Involved in oxidative stress resistance

Table S22- Phytohormone, polyamines and monoamine modulation genes

Locus tag/operon	Gene/operon	Product/putative product	Function/Putative function
SGBXF1_03481	*ipdC*	Indole pyruvate decarboxylase	IAA biosynthesis
SGBXF1_04163	*iaaasp*	IAA-aspartate hydrolase	Degradation of IAA-aspartate
SGBXF1_03089-03103	*paaABCDEFGHIJK*	1,2-phenylacetyl-CoA epoxidase and other elements	PAA degradation
SGBXF1_02759	*yvdD*	LOG family protein	Cytokinin production
SGBXF1_03911	*ygdH*	LOG family protein	Cytokinin production
SGBXF1_02336	*xdhA*	Xanthine dehydrogenase	Cytokinin modification
SGBXF1_02337	*xdhB*	Xanthine dehydrogenase	Cytokinin modification
SGBXF1_03348	*menF*	Isochorismate synthase	Salicylate biosynthesis
SGBXF1_03497	*entC*	Isochorismate synthase	
SGBXF1_03572	*pchB*	Isochorismate pyruvate lyase	
SGBXF1_04255	*puuE*	4-aminobutyrate aminotransferase	GABA degradation
SGBXF1_04254	*gabD*	Succinate-semialdehyde dehydrogenase	
SGBXF1_04074	*speA*	Arginine decarboxylase	Putrescine production via L-arginine
SGBXF1_04070	*speB*	Agmatinase	
SGBXF1_00454	*speC*	Ornithine decarboxylase	Putrescine production via L-ornithine
SGBXF1_04140	*speF*	Ornithine decarboxylase	
SGBXF1_04100-101	*speDE*	S-adenosylmethionine decarboxylase, Polyamine aminopropyltransferase	Spermidine biosynthesis
SGBXF1_03876-77	*cadAB*	Lysine decarboxylase Cadaverine/lysine antiporter	Cadaverine biosynthesis
SGBXF1_02442	*ddc*	L-2,4-diaminobutyrate decarboxylase	1,3-diaminopropane biosynthesis
SGBXF1_02443	*dat*	Diaminobutyrate--2-oxoglutarate aminotransferase	
SGBXF1_03604 SGBXF1_03660	*speG*	Spermidine N(1)-acetyltransferase	Protection against polyamine toxicity
SGBXF1_02813 SGBXF1_02814 SGBXF1_04618	*mdtJ*	Spermidine export protein	Protection against polyamine toxicity
SGBXF1_2080-84	*puuABCD*	Gamma-glutamyl-gamma-aminobutyrate hydrolase and others	Putrescine degradation, GABA formation
SGBXF1_03712	*patD*	Gamma-aminobutyraldehyde dehydrogenase	Putrescine degradation, GABA formation
SGBXF1_00557	*moaEF*	Monoamine oxidoreductase	Conversion of monoamine compounds or their metabolites

Table S23- Genes involved in mixed acid fermentation and VOC production

Locus tag/operon	Gene/operon	Product/putative product	Function/Putative function
SGBXF1_03381	*ackA*	Acetate kinase	Acetate formation
SGBXF1_02050	*icd*	Isocitrate dehydrogenase [NADP]	2-oxoglutarate formation
SGBXF1_04118	*fumA*	Fumarate hydratase class I, aerobic	Succinate formation
SGBXF1_02310	*fumC*	Fumarate hydratase class II	Succinate formation
SGBXF1_0274	*adhE*	Aldehyde-alcohol dehydrogenase	Ethanol formation
SGBXF1_01314	*dld*	D-lactate dehydrogenase	(*R*)-lactate formation
SGBXF1_02633	*ldhA*	D-lactate dehydrogenase	
SGBXF1_03968	*lldD*	L-lactate dehydrogenase	(*S*)-lactate formation
SGBXF1_03509	*budA*	Acetolactate synthase	Acetoin production
SGBXF1_03508	*budB*	α-acetolactate decarboxylase	
SGBXF1_04219	*dhaD*	Glycerol dehydrogenase	Glycerol fermentation Can also oxidize 1,2-propanediol and 2,3-butanediol
SGBXF1_03274 SGBXF1_03945	*dmsA*	Dimethyl sulfoxide/trimethylamine N-oxide reductase	DMS production
SGBXF1_04360-61	*mrsPQ*	Methionine-sulfoxide reductase	
SGBXF1_04667	*ilvE*	Branched-chain-amino-acid aminotransferase	Amino acid degradation
SGBXF1_00918	*ipdC*	Indole-pyruvate decarboxylase	Transforms pyruvate to acetaldehyde
SGBXF1_03481	*ipdC2*	Indole-pyruvate decarboxylase	
SGBXF1_02428	*adhP*	Alcohol dehydrogenase 1	Involved in the production of alcohols, aldehyde or ketone
SGBXF1_03723	*adhB*	Alcohol dehydrogenase 2	
SGBXF1_04185	*adh2* *ykhD*	Long-chain-alcohol dehydrogenase 2 Alcohol dehydrogenase	Involved in the production of long chain alcohols, aldehyde or ketone
SGBXF1_02745	*adhE*	Aldehyde-alcohol dehydrogenase	Involved in the production of alcohols, aldehyde or ketone
SGBXF1_01978	*xylB*	Benzyl alcohol dehydrogenase	Involved in the production of benzyl alcohols, benzyl aldehyde benzyl ketone

Table S24- Genes involved in fatty acid metabolism

Locus tag/operon	*Gene/operon*	Product/putative product	Function/Putative function
SGBXF1_04363-64	*accBC*	Acetyl-coa carboxylaseand carrier	Fatty acid biosynthesis
SGBXF1_03884	*accA*	Acetyl-coa carboxylase carboxyl transferase subunit alpha	
SGBXF1_03397	*accD*	Acetyl-coa carboxylase carboxyl transferase subunit beta	
SGBXF1_01919	*fabD*	Malonyl coa-acyl carrier protein transacylase	
SGBXF1_01918<br>SGBXF1_02920	*fabH*	3-oxoacyl-[acyl-carrier-protein] synthase III	
SGBXF1_00824<br>SGBXF1_03431	*fabB*	3-oxoacyl-[acyl-carrier-protein] synthase I	
SGBXF1_00827<br>SGBXF1_01714<br>SGBXF1_01922	*fabF*	3-oxoacyl-[acyl-carrier-protein] synthase II	
SGBXF1_00130<br>SGBXF1_00136<br>SGBXF1_00559<br>SGBXF1_00826<br>SGBXF1_01355<br>SGBXF1_01920<br>SGBXF1_03834<br>SGBXF1_04382	*fabG*	3-oxoacyl-[acyl-carrier protein] reductase	
SGBXF1_02405<br>SGBXF1_03889	*fabZ*	3-hydroxyacyl-[acyl-carrier-protein] dehydratase [EC:4.2.1.59]	
SGBXF1_01716	*fabA*	3-hydroxyacyl-[acyl-carrier protein] dehydratase / trans-2-decenoyl-[acyl-carrier protein] isomerase	
SGBXF1_01972	*fabV*	Enoyl-[acyl-carrier protein] reductase / trans-2-enoyl-coa reductase (NAD+)	
SGBXF1_00680<br>SGBXF1_02496<br>SGBXF1_02808	*lcfH*	Long-chain acyl-coa synthetase<br>Long-chain-fatty-acid--coa ligase	
SGBXF1_00870	*fadE*	Acyl-coa dehydrogenase	
SGBXF1_00279	*fadB*	Fatty acid oxidation complex subunit alpha	
SGBXF1_03441	fadJ	Fatty acid oxidation complex subunit alpha	
SGBXF1_00278<br>SGBXF1_03442	*fadA*	Acetyl-coa acyltransferase	
SGBXF1_03444	*fadL*	Long-chain fatty acid transport protein	
